# Ethnobotanical Review and Dataset Compiling on Wild and Cultivated Plants Traditionally Used as Medicinal Remedies in Italy

**DOI:** 10.3390/plants11152041

**Published:** 2022-08-04

**Authors:** Stefania Monari, Maura Ferri, Mirko Salinitro, Annalisa Tassoni

**Affiliations:** Department of Biological, Geological and Environmental Sciences, University of Bologna, 40126 Bologna, Italy

**Keywords:** medicinal plants, human health, traditional knowledge, Italian folk remedies, ethnobotany

## Abstract

Over the centuries, wild plants have constituted the main food ingredients and traditional medicine in rural communities. In the last decades, thousands of ethnobotanical studies have been conducted, with the aim of documenting the traditional knowledge on wild and cultivated plants both for food and therapeutic purposes. In the present work, 75 published papers related to Italian ethnobotanical knowledge on wild and cultivated plants traditionally used for medical purposes were analyzed and data on 1117 different species organized in the first dataset to target medicinal applications only. For each plant species, the Italian region of use, plant organs, mode of preparation, specific pathological group of application, citation index, and use index were listed. The different therapeutic applications were subdivided into nine main pathological groups according to the targeted human apparatus. Overall, the cited species with highest number of uses were related to the treatment of the digestive system and skin-ears-eyes-hair diseases, followed by diseases of the genito-urinary and respiratory systems. The 13 most relevant species were identified on the basis of their citation and use indexes. The present review on Italian medicinal flora aims to provide valuable information on wild and cultivated species, which are potential sources of plant-based therapeutic remedies, to preserve and reevaluate endangered traditional folk knowledge.

## 1. Introduction

Plants play an important role in human nutrition and health, due to the high nutritional value given by their well-known bioactive compounds (proteins, fibers, polyphenols, vitamins, etc.). Over the centuries in rural communities worldwide, wild edible plants, growing spontaneously in their natural habitat without being cultivated, have constituted the main food and medicine ingredients.

In the last decades, thousands of ethnobotanical studies have been conducted to document the traditional knowledge on wild and cultivated healthy plants, in cultural, social, and economic contexts [1,2,3,4]. Ethnobotany has applications in many fields, and studies the relationships between people and plants and conservation of biodiversity (with special regard to the documentation and maintenance of indigenous and local botanical knowledge), and records information on locally available species, which are potential sources of new natural drugs as alternatives to synthetic ones [5,6,7].

In general, nowadays, such traditional local knowledge is mainly preserved by elderly people, even though in the last years, the interest in these plant species has again been increasing [8,9]. Traditional medicine is still practiced in many territories and in developed countries, both by people who are involved and not involved in agricultural practices [10,11]. In addition, wild plants were used in the past and are still used today for many different practices, such as feed and veterinary, agricultural, cosmetic, domestic, and magic and ritual uses [3,12,13,14,15].

The Mediterranean Basin is a territory with wide plant diversification due to its mild climate. This area has been populated since millennia and much traditional knowledge of the use of plants has thus been passed through the generations [16,17,18]. In particular, the common daily Mediterranean diet is characterized by significant intake of vegetables, fruits, and spices [19] and both cultivated and wild food species may represent an essential part of this diet, not only for their nutritional value but also for their medicinal properties [4,11,20].

Italy is located in the middle of the Mediterranean Sea in southern Europe. Because of the north–south extension of the Italian Peninsula and the mountainous hinterland, the climate of Italy shows different patterns. In the Northern Alps, the climate is strongly dependent on the elevation, with mild to cool summers and mostly cold winters. Northern and central plain regions mostly show a humid subtropical climate with cool winters and hot summers while coastal areas and a large part of the south are characterized by mild winters, rainy autumns and springs, and hot and dry summers. The heterogeneous climate has led to the diversification of endemic flora and to the diversification of its use among regions. Up to now, ethnobotanical reviews conducted on Italian species have focused on the use of wild plants as food [3,9] or on medicinal species from specific geographic areas [13,21].

In this panorama, it seemed of primary importance to compile a complete checklist of Italian wild and cultivated plants with medicinal effects to interconnect this knowledge with their known benefits on human health and to complement previous studies targeting mostly food applications (e.g., [3,9]) or specific geographic areas (e.g., [13,21]). The present study represents a wide and detailed bibliographic search of all Italian wild and cultivated plant species already studied from ethnobotanical and phytochemical points of view. The aim of this review was the creation of the first dataset in which all the information on wild and cultivated medicinal plants traditionally used in Italy was collected and standardized. This study focused only on medicinal uses while culinary and veterinary uses were not considered.

## 2. Methods

Data collection was carried out by means of an extensive bibliographic search of all the published papers related to Italian ethnobotanical studies. The search was conducted through the Scopus (https://www.scopus.com, last access on 24 March 2022) and Google Scholar (https://scholar.google.com, last access on 24 March 2022) websites. The combination of exact keywords “ethnobotanical study” AND “medicinal plant” AND “Italy” was used. The search produced an output of 489 papers, published from 1980, related to Italian alimurgic plants. After reading all 489 papers, only 75 (2 of which were in the Italian language), which dealt with Italian species with therapeutic uses, were finally selected and analyzed in detail (last update March 2022). All papers or information referring only to the use of plants as food and those related to veterinary or agronomic uses were excluded. The botanical names of plant species were checked and updated according to the website The World Flora Online (http://www.worldfloraonline.org, last access on 26 July 2022).

A complete dataset was created in an Excel worksheet (Table S1) including the following information for each plant species (listed by genus alphabetic order): botanical name of the species/subspecies, botanical family, wild or cultivated status, number of citations, references, pathological groups, citation index (CI), and use index (UI).

The therapeutic uses were classified into 9 different pathological groups: (1) digestive system diseases, (2) skin-ears-eyes-hair diseases and wounds, (3) systemic disorders, (4) genito-urinary system diseases, (5) respiratory system diseases, (6) nervous system diseases, (7) cardio-circulatory system diseases, (8) muscular-skeletal diseases, and (9) metabolic diseases.

The ethnobotanical information collected was evaluated and compared by calculating two different indexes: citation index (CI) and use index (UI). CI was calculated by dividing the number of citations of a given species by the total number of analyzed papers (75). UI was obtained by dividing the number of pathological groups for which a species was used by the total number of pathological groups (9).

Graphical elaborations were carried out using R 4.0.2 software (https://cran.r-project.org, last access on 14 April 2022), Microsoft Excel©, and Paint© 11.2201.22.0 for Microsoft 365. To avoid overlapping, points were randomly scattered on the y-axis (±0.1) using the R function *geometry jitter*.

## 3. Results and Discussion

### 3.1. Italian Literature Data

A total of 75 papers were selected and analyzed (Table 1), of which 72 were original research papers and 3 were reviews, describing ethnobotanical surveys on the traditional uses of plants for human diseases in specific Italian regions. A complete dataset about the wild and cultivated plant species traditionally used in Italy for medicinal purposes was produced (Table S1).

All studies were carried out by interviewing people (mostly elderly) born or living in the studied area for a long time.

**Figure 1:**
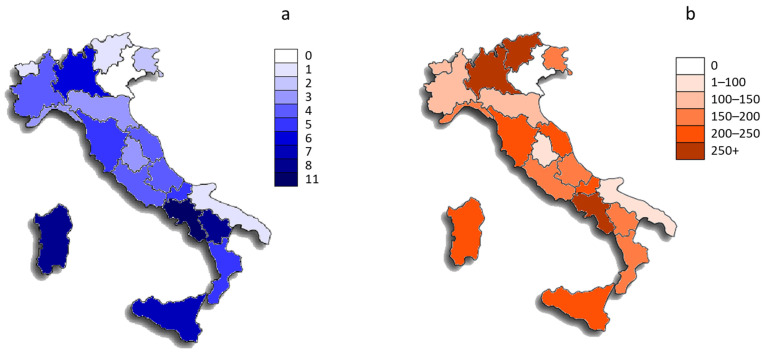
Maps showing the number of ethnobotanical studies that included medicinal applications (**a**) and the number of wild and cultivated plant species used for medicinal purposes (**b**) at the Italian regional level.

Figure 1a shows the number of ethnobotanical studies that included medicinal applications for each region. The data showed that a total of 19 regions (out of 20) were analyzed in these studies, with only the Veneto region missing from any paper. Campania was the most quoted (in 11 papers), followed by Basilicata and Sardegna (8 papers) and Sicilia (7 papers). On the other hand, considering the total number of different medicinal species recorded in each region (Figure 1b), Italian regions with the highest number of analyzed plants were Campania (520 species), Trentino Alto-Adige (269 species), and Lombardia (251 species).

### 3.2. Medicinal Plants

Data were collected on 1117 species, including 42 subspecies, belonging to 125 botanical families. In 69 cases, only the genus was indicated while the species was unspecified (sp.pl., species plures). The families mostly quoted were Compositae with 145 species (12.97%), followed by Lamiaceae (86, 7.69%), Rosaceae (66, 5.90%), and Fabaceae (64, 5.72%) (detailed data in Table S1). Conversely, by analyzing only the food uses, Paura et al. [9] found that the most quoted families of wild plants were Asteraceae and Brassicaceae (20.22% and 6.98%, respectively).

**Figure 2:**
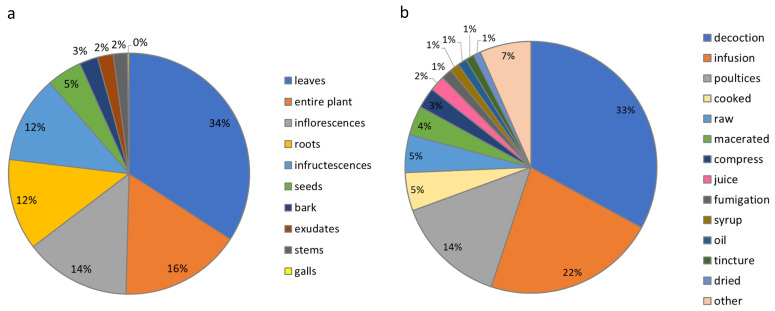
Plant parts used for medicinal preparations. (**a**) Type of remedy preparations and applications. (**b**) Other preparations include liqueur, ointment, crushed, milled, chewed, jam, eaten, cooking water, powdered, oleolite, washes, rubbed, extract, pulped, distilled, smoked, sniffed, and kept in pockets. The classifications used were based on standardized descriptors as reported by Cook [91].

The plant organs that were used most for the medicinal preparations (Figure 2a) were the leaves (34%); entire plant (16%), generally referring only to the aerial parts, including buds and branches; inflorescences (14%) (whole or specific parts, such as petals, stamen, etc.); infructescences (12%); and roots (including tubers, rhizomes, bulbs) (12%). Seeds and their processed parts, such as flour, were used for 5% of the preparations while the remaining 7% of medicinal remedies were based on bark, bulbs, stems, latex, or other plant parts (Figure 2a).

The spectrum of preparation methods was more diversified (Figure 2b). One-third of the medicinal remedies were prepared as a decoction (33%) (the plant is added to cold water, heated and boiled for a few minutes, and the mixture is left to stand at room temperature and filtered) while 22% as an infusion (the plant is added to boiling water and left to stand at room temperature for a few minutes). These data are in agreement with Paura et al. [9], who claimed that infusion and decoction methodologies are mainly associated with medicinal properties and excluded them from their study based on the food use of wild plants. In addition, the plant was directly spread on the part to be treated (as poultices, including bandage, cataplasm, and cream) (14%). A wide range of preparation methods have been indicated in the literature among which raw (5%), maceration (4%), compress made with plant (3%), juice extraction (2%), syrup, oil, tincture, dried fumigation (all 1%), and others (7%) were identified (Figure 2b).

### 3.3. Pathological Groups

Given the very large spectrum of disorders and diseases addressed by medicinal remedies made with wild and cultivated plants, the therapeutic uses quoted in the reviewed literature were classified into nine different pathological groups (Table 2, Table S1), which fit best with the data collected in the present study and also partially taking into consideration previous standardized categorizations [91]. Table 2 shows the resulting nine pathological groups and resumes for each of the uses and treated diseases quoted by the informants.

Overall, the detected plants were mostly used for the treatment of the digestive system and skin-ears-eyes-hair diseases and wounds groups (with more than 600 plant species each), which also showed the highest number of targeted diseases. This observation may be explained by the fact that most of these ailments are generally mild and easy to cure with plants [47]. About 500 species were used for diseases of the genito-urinary and respiratory systems. A similar number of species were utilized for the more general systemic disorders category. These results are in agreement with the findings of several other ethnobotanical studies [45,54,63].

Brief descriptions of the pathological groups with a few examples of plant species applications are presented in the following sections.

#### 3.3.1. Digestive System Diseases

In total, 74 papers listing 705 species described different ways to treat digestive system ailments (Table 2, Table S1). This pathological group is composed of digestive system diseases, parasitic and helminthic infections, and teeth and mouth problems.

A total of 190 species were cited for their digestive properties, principally after drinking an infusion or decoction or after alcoholic maceration. The seeds of *Foeniculum vulgare* Mill., leaves of *Laurus nobilis* L., flowers and leaves of *Rosmarinus officinalis* L., and leaves of *Salvia officinalis* L. were the most utilized plant organs [14,60,73,78].

Gastric ailments (total of 62 species), such as gastritis and nausea, were treated mostly by drinking a decoction or infusion of specific plant organs, for instance, a decoction of *Hordeum vulgare* L. fruits [45] or *Cynara cardunculus* L. leaves [81] and an infusion of *Citrus limon* (L.) Osbeck fruits or *Ocimum basilicum* L. aerial parts [23]. However, the consumption of raw or cooked seeds of *Vicia faba* L. [44], fruits of *Capsicum annuum* L. [43], or leaves of *Oxalis acetosella* L. [59] was also used against heartburn.

Regarding intestinal disorders (219 species), the most common symptoms treated with plants were the simplest ones, such as diarrhea, belly pains, and constipation, which are easy to solve with immediate administration of the treatment. The leaf decoction or raw fruits of *Arbutus unedo* L. [43,44] and raw fruits of *Sorbus domestica* L. [28,52] were the most cited remedies to solve diarrhea. Moreover, several plants were used against constipation and for their carminative properties such as *Mercurialis annua* L., *Prunus* sp.pl., *Daucus carota* L., *Petroselinum crispum* (Mill.) Fuss, and *Carum carvi* L. [37,46]. Other plants were used in the case of anorexia and inappetence. *Gentiana* sp.pl. was the most representative genus utilized for this remedy (*G. acaulis* L., *G. dinarica* Beck, *G. lutea* L., *G. punctata* L., *G. verna* L.) [14,23,65].

Several liver protectors (90 species) were also mentioned by the interviewed people. A decoction of the flowers and leaves and an infusion of the roots of *Cichorium intybus* L. showed purifying and hepato-protective effects [59], and cooking water and cooked leaves of the same plant were eaten for liver wellbeing [26]. A leaf and root decoction of *Taraxacum campylodes* G.E.Haglund was used as a depurative for the liver and the boiled leaves were eaten as a depurative [14,54] in addition to the boiled leaves or decoction of *Silybum marianum* (L.) Gaertn. [67]. The liver-protecting characteristic of these species is essentially due to their cholagogue, choleretic, and depurative activities [90].

Here, parasitic and helminthic infections (73 species) were considered part of the intestinal tract pathological group because the plant treatments cited were mostly against enteric parasites. *Allium sativum* L. was the most widely used plant for vermifuge and anti-helminthic action, with many different uses and preparations for this purpose, for example, drinking bulb juice [50] or crushed bulb decoction [37,39], eating raw crushed bulbs [65,84], bulb necklace, or vapor inhalation [35]. Other species used to treat parasitic diseases were *Artemisia absinthium* L. (aerial part infusion or maceration in alcohol) [14,22,67], *Ruta chalepensis* L. (leaf infusion or aerial part maceration in oil) [33,90], and *Ricinus communis* L. (seed oil) [40].

Several species (179) were reported for their anti-inflammatory properties for the mouth (72), teeth (137), and gums (18). The leaves of *Lactuca sativa* L. were widely reported in ethnobotanical studies as anti-odontalgic and for the treatment of toothache: a decoction of the leaves was used for washes against mouth inflammations and the boiled leaves were used on inflamed teeth [39,43]. The whole plant of *Malva sylvestris* L. was the most reported species for the treatment of oral cavity ailments (50 citations), with a decoction of the aerial parts used as a mouthwash for toothache [52] or the flowering parts as an antiseptic against caries [65], pills obtained after boiling and pressing the plant used to treat gum inflammation [61], and chewing of leaves [63] and washes with a decoction of the roots [39] used in toothache.

#### 3.3.2. Skin-Ears-Eyes-Hair Diseases and Wounds

In total, 72 papers quoted 661 different species (Table 2, Table S1) as cicatrizing of skin injuries (wounds, cuts), emollients (rash, erythema, sunburns, dermatitis), lenitives (bruises, ecchymosis, insect stings, chilblains), and for cosmetic issues such as furunculosis, corns, hair loss, varicose veins, chapped hands, or wound healing. Treatments for ear and eye inflammation were mainly topic preparations.

Many species (144) were used as emollients in the case of dermatitis, rash, and other skin irritations. Tubers of *Solanum tuberosum* L. were used in various ways, for example, raw slices were used on skin burns [39,44], boiled potato slices were used on the affected area [37], fresh pulp was externally applied to minor burns and reddened skin [89], and cataplasm made with raw tuber was applied as a paste [72]. Decoctions with *Malva sylvestris* L., both with the roots [74] and the flowering aerial parts [30], were used to relieve dermatitis, in addition to the peeled stems of *Opuntia ficus-indica* (L.) Mill [39,76] and the whole flowering plant of *Viola odorata* L. [40]. Seed oil of *Prunus dulcis* (Mill.) D.A.Webb [72] and a decoction of the flowers or leaves of *Malva neglecta* Wallr. and *Urtica* sp.pl. leaves [90] were used against eczema and sunburns.

Folk medicine remedies against bruises, chilblains, and similar skin problems were also widespread (118 species). The crushed aerial parts of *Parietaria judaica* L. (together with the seeds of *Linum usitatissimum* L. and egg albumen) [37,44], aerial part infusion of *Artemisia absinthium* L. [68], and flower oil of *Hypericum perforatum* L. and *H. perfoliatum* L. [81] were utilized in poultice recipes that were topically applied with lenitive properties against bruises. To relieve itching and swelling due to chilblains, known remedies were directly applied leaves or fruits of *Citrus sinensis* (L.) Osbeck [43], resin of *Larix decidua* Mill. (sometimes cooked in oil or butter) spread on the affected area [14], and grated and boiled rhizome of *Iris germanica* L. directly placed on the skin [36].

Plants (81) with skin lenitive properties were also used to heal insect bites and stings. Among them, the most cited were fresh bulbs of *Allium cepa* L. and *A. sativum* L. rubbed on the skin [45,63], fresh leaves of *Plantago lanceolata* L. and *Plantago major* L. applied to bee and mosquito bites [52,54], cataplasm made with the aerial parts of *Clinopodium nepeta* (L.) Kuntze [81,88], and directly applied fruit latex of *Ficus carica* L. [30,34].

Concerning plants involved in dermatological problems, a considerable set of species (108) were used for several cosmetic issues. Furunculosis benefitted from the uses of several plants such as topically applied fresh leaves and fruits of *Rubus ulmifolius* Schott [81], fresh leaves of *Ocimum basilicum* L. [37], flower infusion of *Borago officinalis* L. [68], compress with the aerial parts of *Malva sylvestris* L. [61], and root decoction of *Arctium lappa* L. [14]. Poultice with the aerial parts of *Sedum maximum* (L.) Suter. [57], latex from the stems of *Chelidonium majus* L. [55], crushed leaves of *Rumex* sp.pl. [54], and cataplasm with the tubers of *Asphodelus microcarpus* Saltzm. & Viv. [90] were applied to treat corns and warts.

Inflammation of the eyes and ears was treated with topical applications of several preparations (90 species). Compress made with a flower infusion or decoction of *Matricaria chamomilla* L. [49,65], leaf decoction [57] or flower infusion [14] of *Sambucus nigra* L., and shoot sap of *Vitis vinifera* L. used as a collyrium [40,44] are examples of good remedies in the treatment of conjunctivitis and inflamed eyes. Topically applied oil from the fruits of *Olea europaea* L. [40,44] and fumigations with the leaves of *Arundo donax* L. [90] were considered effective in the case of otitis.

Hair loss and dandruff were also treated with medicinal plants (16): a leaf decoction of *Urtica dioica* L. was widely drunk or more often externally applied in washes to strengthen hair and prevent hair loss [48,61,69] and a decoction of the aerial parts of *Parietaria officinalis* L., leaves of *Origanum vulgare* L., or *Urtica* sp.pl. was considered effective against dandruff [23,45,48]. A bandage and poultice made with some plant organs were used as a moisturizing cream for chapped skin (e.g., flowering tops of *Achillea millefolium* L. [61] or inflorescences of *Calendula officinalis* L. [55]).

Several plants (196) were used for healing wounds: a poultice made with the leaves of *Plantago major* L. [53,57], warmed leaves of *Malva sylvestris* L. [32,79], cataplasm with the crushed seeds of *Linum usitatissimum* L. [41,57], direct application of the resin of *Pinus* sp.pl. [68,90], and rubbing with the flower oil of *Hypericum perforatum* L. [69,82] were some of the most popular vulnerary remedies.

#### 3.3.3. Systemic Diseases

In total, 531 plant species were reported in 70 papers (Table 2, Table S1) as useful in the case of systemic disorders to improve the general state of health. These plants were not effective against a specific affection but in curing and strengthening the whole organism.

Some of these plants (166) were defined as a reconstituent tonic, depurative, or detoxicant to help in the recovery from different diseases by purifying the blood and inducing perspiration. A leaf decoction of *Cichorium intybus* L. was drunk before meals as a depurative of the intestine and blood [19,54,58] while a leaf or flower decoction of *Taraxacum campylodes* G.E. Haglund was used as a kidney depurative [14,56]. Infusions or decoctions drunk as detoxifiers were made with the roots of *Cynodon dactylon* (L.) Pers, aerial parts of *Melissa officinalis* L., aerial parts of *Urtica dioica* L., and seeds of *Foeniculum vulgare* Mill. [49,83].

Other plants (48) were identified as being analgesic and lenitive and effective in relieving different types of pain due to their general soothing action. The aerial parts of *Parietaria judaica* L. were topically applied for their analgesic effects [43], a poultice made with the macerated petals of *Rosa canina* L. was a lenitive for the skin [90], and flowers and other aerial parts of *Hypericum perforatum* L. macerated in olive oil were locally applied as a lenitive in a wide range of inflammation [77].

Other species were considered as fortifiers due to their corroborant properties and their capacity of contrasting weakness. The fresh aerial parts of *Artemisia genipi* Weber ex Stechm. and *Artemisia umbelliformis* Lam. were chewed during exercise as a tonic and energizer [59], fresh fruits of *Juglans regia* L. were eaten as mineralizing and energizing snacks [57], and raw seeds of *Prunus dulcis* (Mill.) D.A. Webb were eaten in the case of weakness [22].

Some species were also used for their antipyretic and immunostimulant action. *Centaurium erythraea* Rafn is well known for the febrifuge activity of a decoction made with the aerial parts [31] or whole plant [55] or a flower infusion [90]. Many species have shown generic antipyretic properties (e.g., *Ajuga iva* (L.) Schreb., *Marrubium vulgare* L., *Juniperus communis* L.) while others have been used specifically for malarial or typhoid fevers, such as *Gentiana lutea* L. (root infusion) [23], *Centaurea calcitrapa* L. (decoction with the aerial parts) [75], *Rhamnus alpina* L. (decoction with fruits) [65], and *Apium nodiflorum* (L.) Lag. (leaf infusion) [74]. A small number of species had immunostimulant effects, including the flowers of *Rosa canina* L. [68] and bulbs of *Allium sativum* L. [44].

#### 3.3.4. Genito-Urinary System Diseases

All 75 papers that were examined cited plant species (in total 522) (Table 2, Table S1) with properties to treat diseases of the urinary tract and genital-reproductive system.

Treatment of urinary tract diseases included remedies for cystitis, enuresis, gall stones, and various renal troubles from 343 species. A widespread remedy for kidney and urinary issues was a decoction of *Cynodon dactylon* (L.) Pers. Rhizomes, which was drunk in the morning on an empty stomach [51] and after 2 h of maceration in water [33]. *Asparagus* sp.pl. (particularly *A. acutifolius* L. and *A. officinalis* L.) were well known for their strong diuretic and depurative action due to their high mineral content [44]. In particular, a decoction of *A. acutifolius* L. shoots or *A. officinalis* L. roots was employed and *A. tenuifolius* Lam. turions were eaten for their depurative and diuretic effects [56]. The most cited species known to be useful against kidney stones and renal colic were *Ceterach officinarum* Willd. and *Cynodon dactylon* (L.) Pers. In addition, a decoction of the leaves, rhizomes, or whole plant of *C. officinarum* Willd was drunk to expel renal calculi [78] and a decoction of the rhizomes or whole plant of *C. dactylon* (L.) Pers. was taken orally against cystitis and kidney stones [37].

Most of the species (136) identified for healing problems in the genital apparatus act on women’s diseases such as menstrual pains (i.e., amenorrhea, dysmenorrhea, irregular menses), delivery problems (i.e., miscarriage, galactagogue or stopping the secretion of milk, uterine bleeding, uterotonic), and hormonal stimulation (i.e., fertility or antifertility effects, estrogenic). The flowers of *Matricaria chamomilla* L. were widely used for menstrual pains as a decoction of fresh flowers [37], infusion of dried flowers [57], or direct application of flowers as a hot unguent [54]. Additionally, the leaves of *Laurus nobilis* L. were useful to treat dysmenorrhea by preparing an infusion with honey [45] or a decoction [37]. Regarding delivery problems, some species were used as partum enhancers: examples include *Rubus idaeus* L. (dried leaf infusion), *Malva sylvestris* L. (bath in leaf infusion), and *Linum usitatissimum* L. (seed oil to drink) [57]. Other species were active in preventing uterine bleeding during or after labor, such as *Punica granatum* L. (decoction of fruit peel), *Capsella bursa-pastoris* (L.) Medik. (infusion of the dried plant), and *Bellis perennis* L. (flower infusion) [40].

Geraci et al. [20], also in accordance with other studies, focused their ethnobotanical survey on several wild plants with galactagogue properties traditionally used by women during breastfeeding to increase milk production. Several species (48) were quoted for this purpose such as the boiled aerial parts of *Borago officinalis* L. [28], the boiled leaves of *Cichorium intybus* L. [43], a seed and leaf decoction of *Foeniculum vulgare* Mill. [37,73], a seed infusion of *Nigella damascena* L. directly applied on the breast [78], and a leaf infusion [14] or the aerial parts of *Urtica dioica* L. rubbed topically [42].

The use of plants to aid miscarriage in unwanted pregnancies is no longer practiced [37], but in the past, a high dosage of active toxic compounds of some species (31) was used as abortifacients. *Petroselinum crispum* (Mill.) Fuss. was a well-known species with abortive effects, for example, via ingestion of either large doses of raw leaves [14,55] or an aerial part decoction [30] while a concentrated preparation of the seeds excited uterine fibers, causing miscarriage [40]. Other abortive preparations were a decoction of the aerial parts of *Adiantum capillus-veneris* L. [41], a flower infusion of *Artemisia absinthium* L. [50], and a vegetative part decoction of *Ruta graveolens* L. [79].

Male genito-urinary diseases treated with plant remedies (from 29 species) mainly include prostate affections. To prevent prostate cancer, a bath of the leaves of *Plantago lanceolata* L. and *P. major* L. [68] and a leaf decoction of *Beta vulgaris* L. [44] were used. To cure prostatitis, a fruit and leaf infusion of *Vaccinium myrtillus* L. [69] and a flower infusion or herbal tea with the aerial parts of *Epilobium angustifolium* L. [14,24] were employed. *Ficus carica* L. latex of unripe fruits [54] and *Euphorbia helioscopia* L. latex [27,28] were used as penis vasodilators.

#### 3.3.5. Respiratory System Diseases

According to 69 scientific papers, 499 species were used to treat respiratory system ailments (Table 2, Table S1). The most frequent uses (specifically from 282 species) were against cold, cough, and inflammation (such as laryngitis, bronchitis, and pneumonia). For example, the leaves of *Salvia officinalis* L. [34] and roots of *Foeniculum vulgare* Mill. [43] were used to make a fumigation against cold and cough, in addition to a flower infusion of *Sambucus nigra* L. [56] and a leaf decoction of *Laurus nobilis* L. [39]. Other species were cited for their action against pneumonia and bronchitis, such as *Opuntia ficus-indica* (L.) Mill. (decoction of cladodes) [41] and *Triticum aestivum* L. (bran poultice topically applied on the chest and back) [54].

A typical south Italian remedy is the so-called *ricotto*, considered a panacea for several respiratory diseases, and consisting of a decoction prepared with several different plant ingredients, boiled in water for 30–60 min, filtered, and cooled down before drinking. This preparation is currently still used for its bechic, expectorant, and anti-asthmatic properties, and the recipe may vary from one person to another. For example, Scherrer et al. [39] provided a recipe composed of *Ficus carica* L., *Prunus spinosa* L., *Pyrus communis* L., *Malus domestica* Borkh., *Prunus armeniaca* L., *Hordeum vulgare* L., *Citrus limon* (L.) Osbeck, *Malva sylvestris* L., *Foeniculum vulgare* Mill., *Cynodon dactylon* (L.) Pers., and *Laurus nobilis* L. as the main ingredients. An alternative is provided by Motti and Motti [37], where the informants mentioned the use of *Plantago lanceolata* L., *Plantago major* L., *Ceratonia siliqua* L., *Prunus dulcis* (Mill.) D.A.Webb., *Citrus reticulata* Blanco, *Clinopodium nepeta* (L.) Kuntze, and *Urtica membranacea* Poir. ex Savigny. Overall, a total of 47 different plant species were cited as the ingredients of *ricotto* in the considered studies.

A peculiar application of plants against asthma was smoking of the dried leaves of three different species: *Tussilago farfara* L. [31], *Rosmarinus officinalis* L. [51], and *Datura stramonium* L. [44].

#### 3.3.6. Nervous System Diseases

A total of 348 species from 70 papers were cited as being beneficial for nervous system diseases (Table 2, Table S1). These plants were reported to act as sedative and calming agents (including treatment against insomnia, anxiety, and epilepsy), as remedies against depression and headache, and as nervous stimulants.

*Papaver rhoeas* L. and *P. somniferum* L. were widely used. A decoction of seeds was used as a sleeping drug [36], flower or fruit infusions were administrated to children to help against insomnia [32,87], a tea obtained from petals was used as a sedative [54], a fruit decoction was used against neuralgia [44], and young shoots were added to other recipes to prevent anxious or nervous states [39]. Flowers of *Matricaria chamomilla* L. were also largely used for neurological applications. In particular, a tea made with flower heads was drunk as a tranquilizer and to treat headache [49,69], a flower infusion was used for its mild sedative and hypnotic properties [5,83], and an inflorescence decoction and a bath with the stems were used as remedies for irritability [44].

The fruit juice of *Citrus limon* (L.) Osbeck [35], leaf decoction of *Rosmarinus officinalis* L. [73], leaf infusion of *Salvia officinalis* L. [32], tincture made with the flowers of *Melissa officinalis* L. [57], and root decoction of *Valeriana officinalis* L. [45] were preparations reported for the treatment of headache.

Depression and Parkinson’s disease were other important neurological affections treated with plant remedies from 12 species. In particular, a flower infusion or tincture of *Hypericum perforatum* L. [24,56], aerial part infusion of *Leonurus cardiaca* L., and aerial part tincture of *Heracleum sphondylium* L. [89] were used as anti-depressives. In Guarino et al. [40], *Atropa bella-donna* L., *Datura stramonium* L., and *Hyoscyamus niger* L. were cited for their use in Parkinson’s disease due to their antiepileptic and antispasmodic effects.

#### 3.3.7. Cardio-Circulatory System Diseases

In total, 72 references reported the use of 333 species for the treatment of cardio-circulatory system diseases (Table 2, Table S1). This group includes actions on blood circulation (hemostatic effects, anti-atherosclerosis, against varicose veins), and blood pressure (against hypertension), and remedies for cardiac troubles.

Various species (16) were used to improve blood circulation. A bandage with a flower infusion of *Achillea millefolium* L. [89] or drinking a leaf infusion of *Fagopyrum esculentum* Moench [57] were known as stimulants of blood circulation in the Lombardia region, a flower infusion of *Crataegus monogyna* Jacq. Was used in Basilicata [27,28], and drinking fruit juice of *Citrus limon* (L.) Osbeck or eating raw fruits of *Capsicum annuum* L. [37] were remedies adopted in Campania. A bandage and poultice made with some plant organs (e.g., leaves of *Brassica oleracea* L. [59], leaves of *Arctium lappa* L., and fruits of *Aesculus hippocastanum* L. [57]) were also frequently topically applied on varicose veins.

Some plants (9) were also used to prevent severe vascular pathologies such as atherosclerosis, including *Arbutus unedo* L. (fruit decoction) [71] and *Crataegus laevigata* (Poir.) DC. (flower infusion) [89].

Different species (59) were utilized to regulate blood pressure in the case of hypertension such as a leaf infusion of *Olea europaea* L. [32,75,86]; chewing of the bulbs, a tincture [45], or raw consumption [30,37] of *Allium sativum* L. [14]; and a leaf decoction of *Cichorium intybus* L. [51,75].

With regard to cardiac diseases, folk medicine mainly concerns cardio-tonic substances or remedies against tachycardia. Species with cardiotonic effects include *Crataegus monogyna* Jacq. (flower infusion [56]), *Nerium oleander* L. (very diluted leaf infusion), and *Marrubium vulgare* L. (cataplasm from the boiled aerial parts) [90].

#### 3.3.8. Muscular-Skeletal Diseases

In total, 68 papers cited 301 plant species known to be useful against muscular-skeletal pathologies, including remedies for sprains, little fractures and weak bones, backache and stiff-neck, arthritis, rheumatisms, and muscular pains (Table 2, Table S1).

The majority of citations (161 species) dealt with the treatment of rheumatic pains. Different preparations of *Urtica dioica* L. showed anti-rheumatic properties: a leaf decoction [74], topical use with vigorous rubbing on the skin of the topical part [38], 40 days of drinking an infusion of the aerial parts [33], the leaves applied as a poultice [5], and a root decoction [31]. *Dioscorea communis* (L.) Caddick & Wilkin (mainly a cataplasm or ointment with the fruits) [70,72], *Rosmarinus officinalis* L. (leaf infusion) [33,57], and *Juniperus communis* L. (berry infusion in grappa or fruit infusion) [49,54] were also often reported for this kind of treatment.

The number of species reported against arthritis and other articular pains was also relevant (79). To treat arthrosis, topical used was common, for example, the flowers of *Hypericum perforatum* L. in an infusion or macerated in oil [5,32], marc of *Vitis vinifera* L. [35,90], a compress made with a bark decoction of *Sambucus nigra* L. [57], crushed leaves of *Dryopteris filix-mas* (L.) Schott. [40], and resin of *Abies alba* Mill. [68]. The same was applied for sprain medications, mainly consisting of a cataplasm, infusion, decoction, or directly application of crushed plants to the affected part. Among the most used plants, the resin of *Picea abies* (L.) H.Karst. or *Abies alba* L. smeared on the interested area [58], the aerial parts of *Centaurea calcitrapa* L. used to make a cataplasm [81], an alcoholic oil maceration of the dried flowers of *Arnica montana* L. used during relieving massages [55], the crushed aerial parts of *Parietaria judaica* L., and a whole plant decoction of *Matricaria chamomilla* L. [44] were identified.

Beneficial effects on backache and stiff-neck were reported after the use of various plants (19), for instance, *Brassica oleracea* L. (roasted leaves applied topically) [30], *Urtica dioica* L. (stems or leaves applied locally) [32], and *Alnus incana* (L.) Moench (compress of dried leaves) [69]. Other preparations (from 18 different species) were used to relieve pain due to sore and swollen legs or feet, such as the use of powdered *Myrtus communis* L. leaves in shoes [25], a cataplasm with the fresh leaves of *Brassica oleracea* L. [34], or soaking feet in a seed decoction of *Sinapis alba* L. [51].

#### 3.3.9. Metabolic Diseases

In total, 223 species (listed in 46 papers) were identified as being active in healing metabolic diseases, such as those showing antidiabetic and antigout activities, cytotoxic action against cancer cell proliferation, and mineral and vitamin integrators (Table 2, Table S1).

Several plants (35) were used for their hypoglycemic and anti-diabetic activities, including *Salvia officinalis* L. (leaf decoction in wine drunk after meals [39]) and *Urtica dioica* L. (cooked leaves [21] or decoction/infusion of the whole plant [75]). Moreover, a jam of the fresh fruits or a leaf infusion of *Vaccinium myrtillus* L. [59] and the leaves of *Morus nigra* L. and *M. alba* L. [40] were reported to stabilize blood sugar levels in diabetic patients. Gout was treated with the bark of *Sambucus nigra* L. (crushed and applied on painful body parts [40] or as a compress or decoction [57]). Other remedies against gout were a fresh root juice or seed decoction of *Apium graveolens* L. [62] and a whole plant decoction of *Polygonum aviculare* L. [44].

To regularize cholesterol metabolism, oral preparations were consumed (drunk or eaten), for instance, a leaf decoction from the basal rosette of *Taraxacum campylodes* G.E.Haglund [77], boiled receptacles of *Cynara scolymus* L. [37], fruit juice of *Citrus limon* (L.) Osbeck [41], and fruits of *Atropa bella-donna* L. macerated in water [23].

A very small number of plant remedies (12) were used as a preventive action against cancer and not as a curative action. A bath with the leaves of *Plantago lanceolata* L. and *P. major* L. was used to prevent prostate cancer in addition to a bath with the whole plant of *Potentilla reptans* L. [68]. *Equisetum arvense* L. (wrapping with plant boiling water) [59] and *Colchicum neapolitanum* (Ten.) Ten. [40] were also cited for their antitumor properties.

With regard to vitaminic and remineralizing properties, 26 wild and cultivated plants were added to recipes as a mineral salt supplement. Some examples were the aerial parts of *Urtica dioica* L., which are cooked and added to salads [45]; the seeds of *Prunus dulcis* (Mill.) D.A.Webb., which are very rich in minerals [22]; and the fresh leaves of *Nasturtium officinale* R.Br., which are used as ingredients in salads, soups, and omelettes [56]. Other plants were used to improve the vitamin intake, such as the pulp or juice of *Ribes nigrum* L. fruits [89]; the fresh aerial parts of *Cardamine amara* L. eaten as a snack, used as an ingredient in salads, or cooked [57]; and the raw roots of *Daucus carota* L. eaten as a source of provitamin A [90].

### 3.4. Most Relevant Italian Medicinal Plant Species

**Figure 3:**
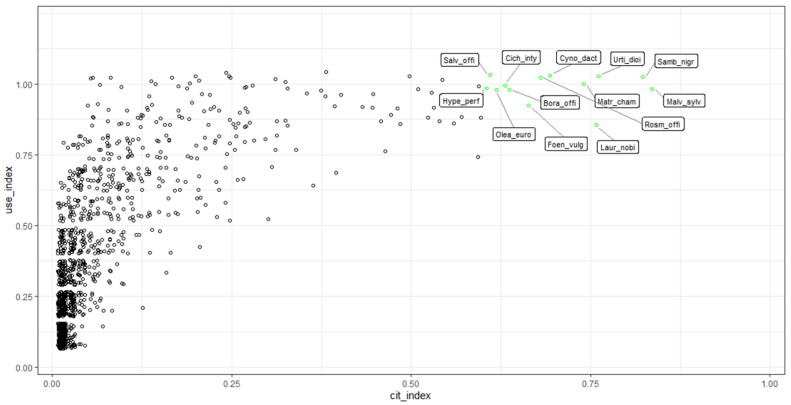
Graphic distribution of the cited medicinal species according to the citation (CI) and use (UI) indexes. To avoid overlapping, dots above 0.8 UI were randomly scattered on the y-axis (±0.1) using the R function *geometry_jitter*. In green, most relevant species with a CI above 0.6 and UI above 0.8 are shown. Malv_sylv, *Malva sylvestris* L.; Samb_nig, *Sambucus nigra* L.; Urti_dio, *Urtica dioica* L.; Matr_cham, *Matricaria chamomilla* L.; Cyno_dact, *Cynodon dactylon* (L.) Pers.; Rosm_offi, *Rosmarinus officinalis* L.; Bora_offi, *Borago officinalis* L.; Cich_inty, *Cichorium intybus* L.; Olea_euro, *Olea europaea* L.; Hype_perf, *Hypericum perforatum* L.; Salv_offi, *Salvia officinalis* L.; Laur_nobi, *Laurus nobilis* L.; Foen_vulg, *Foeniculum vulgare* Mill.

For each species considered in the present study (Table S1), the citation index (CI, number of citations of the species/75 (total number of analyzed papers)) and the use index (UI, number of pathological groups for which the species was used/9 (total number of pathological groups)) were calculated to analyze the collected ethnobotanical information in order to identify the most relevant wild and cultivated Italian plant species used for medicinal purposes. A graphical representation of all 1117 cited species according to their CI and UI (see the detailed data in Table S1) is shown in Figure 3. The 13 plant species highlighted in green (Figure 3) represent those showing both the highest number of citations (CI above 0.6) and the highest number of therapeutic applications (UI above 0.8) distributed in different pathological groups. Interestingly, in all interested Italian regions, all highly cited species were classified as spontaneously growing wild or both wild and cultivated. None of them were only cultivated.

A short summary of the preparation methods and the therapeutic use of each of the most relevant 13 species follows (see more details and references in Table S1).

*Malva sylvestris* L. (CI 0.84, UI 1.00). Mallow was reported to be an excellent medicinal plant with many different therapeutical uses. The flowers and leaves were used to make decoctions and infusions useful for diverse skin and mouth inflammations, gastrointestinal diseases, vaginal and urogenital system inflammations, and respiratory ailments. A decoction made with the roots was an excellent remedy to treat many affections such as cough, sore throat, stomach ache, toothache, menstrual pain, hypertension, dermatitis, and weakness. In other countries, such as the Iberian Peninsula and Turkey, mallow was also used as an antipyretic [92] and for abdominal pain [93].

*Sambucus nigra* L. (CI 0.82, UI 1.00). Many different preparations made with the flowers and bark were used against a wide range of affections. A decoction, infusion, or syrup made with the flowers were drunk to treat cough, cold, sore throat, bronchitis, fever, headache, toothache, hypertension, and abdominal pain. A cataplasm, oleolite, decoction, infusion, or boiled flowers were topically applied on bruises, rheumatisms, dislocated bones, eye irritation (infusion as a collyrium), and metabolic and skin diseases (i.e., gout, skin redness, hematomas, wounds). The bark also has other applications: topically on burns and sores as a lenitive, to cure arthritis, as a laxative and diuretic, and used as a systemic anti-inflammatory. The same uses were also reported in many other Mediterranean countries such as Greece [94], Spain [92], and Portugal [95].

*Urtica dioica* L. (CI 0.76, UI 1.00). Nettle was another widely recognized medicinal plant. The leaves were used both internally as an expectorant, diuretic, depurative, digestive, and galactagogue and to treat anemia, cold, and cough; and externally to treat dandruff and hair loss, dermatitis, painful joints, rheumatisms, chilblains, and wounds. The roots were used internally as an antirheumatic, to treat abdominal pain and colitis, and to fight gastric and duodenal ulcers. The aerial parts were used for liver disorders, intestinal inflammation, water retention, and to treat epistaxis and toothache. These Italian methods of preparation are in agreement with those cited, among others, in Spain, Serbia, and Morocco [92,96,97].

*Matricaria chamomilla* L. (CI 0.74, UI 1.00). The flower heads of chamomile were widely used to treat cold and respiratory system diseases, digestive system disorders, and neurological problems (against irritability and to promote sleep). It was also used against menstrual and muscular pains and as a collyrium for tired eyes and conjunctivitis. Similar applications have also been identified worldwide (e.g., Mexico, Serbia, Bulgaria, and Pakistan [96,98,99,100]).

*Cynodon dactylon* (L.) Pers. (CI 0.69, UI 1.00). The roots and rhizomes were used for a wide variety of diseases (also as an ingredient of a *ricotto* decoction), including cystitis, hepatitis, renal stones, cough and flu, fever, rheumatisms, dysmenorrhea, abscesses, and prostatitis. Conversely, an extract of the whole plant was used both orally and topically against respiratory disorders and skin diseases in Pakistan [98] and India [101].

*Rosmarinus officinalis* L. (CI 0.68, UI 1.00). Rosemary is a typical Mediterranean plant with similar therapeutic applications in many worldwide countries [92,94,102]. The aerial parts, mainly the leaves, had many phytotherapeutic uses, including a local analgesic, anti-septic, sedative, spasmolytic, anti-pyretic, and energetic, and to treat cough, constipation, bronchitis, and asthma. It was an ingredient of the Italian *ricotto* decoction.

*Borago officinalis* L. (CI 0.64, UI 1.00). Borage has been widely reported in ethnobotanical research for its diuretic, emollient, expectorant, galactagogue, diaphoretic, and anti-inflammatory properties. Among the various affections treated with borage preparations, some examples are cold, cough, lung diseases, stomach ache, intestinal regulation, abdominal pains, reddened skin, pimples, eczema, burns, toothache, and gout (topical use). Its use is also reported in other countries, such as in Algeria [103] to treat depression and other neurological disorders, and in the Spanish Balearic Islands [104] as an antipyretic and anticatarrh.

*Cichorium intybus* L. (CI 0.64, UI 1.00). The leaves and aerial parts were used mostly for their diuretic, laxative, and depurative properties, in accordance with other countries’ reports [2,98], and the roots were used to protect and purify the liver, to treat hypertension, and as an anti-acne agent.

*Olea europaea* L. (CI 0.62, UI 1.00). Preparations with the leaves and fruit oil of the olive tree were used for a wide range of affections. A leaf decoction was used both internally, drunk as a diuretic, hypotensive, and febrifuge and to treat gastrointestinal disorders, and externally to heal gout, wounds, and other dermatological problems. The fruit oil was used for its antiseptic, anti-inflammatory, and vulnerary properties against otitis, burns, hemorrhoids, sun rash, acne, rheumatisms, and sprains. When the oil is drunk, it has laxative and hypotensive properties. The leaves were widely used in Turkey as a herbal tea infusion to reduce blood pressure and diabetes or were chewed to heal oral wounds [105].

*Hypericum perforatum* L. (CI 0.61, UI 1.00). *Hypericum* flowers were widely used in phytotherapy, not only in Italy but all over the world [93,96,100,101]. An infusion to aid digestion, as a cholagogue and anti-depressant, to treat constipation and sleep disorders, with relaxing properties is used. A maceration in olive oil is applied to burns, sores, skin rash, wounds, contusions, and erythema. A decoction (also an ingredient of *ricotto*) was drunk to treat gastrointestinal and hepatic colic as a digestive and blood depurative. Moreover, the leaves and other aerial parts were used for menstrual pains, arthrosis, joint pain, and rheumatisms.

*Salvia officinalis* L. (CI 0.61, UI 1.00). The plant organs mostly utilized were the leaves, which have many therapeutical uses, including toothache and other mouth problems, stomachache, respiratory ailments, headache, and febrifuge. In some North African states, this plant was also used as an appetizer, anti-diarrheic, and anti-diabetic [103,106].

*Laurus nobilis* L. (CI 0.76, UI 0.89). In addition to being used in food recipes, laurel is also a medicinal plant widely present in Italian popular medicine and worldwide [93,103,105]. Most of the preparations for curative purposes utilized a laurel leaf decoction or infusion for external use (rheumatisms, arthritis, insect bites, contusions) and internal use (gastrointestinal pains, abdominal colic, cold, cough, stimulant of blood circulation, menstrual pain, and galactagogue action).

*Foeniculum vulgare* Mill. (CI 0.66, UI 0.89). Fennel was widely consumed in cooking and largely used in folk medicine. It was used for gastrointestinal disorders (poor digestion, aerophagia, stomachache, colic, gastric acidity, etc.), respiratory issues (sore throat, cold, cough, bronchitis), female ailments (galactagogue and emmenagogue action, to fight dysmenorrhea), and irritability and swelling and as an anti-pyretic, anti-rheumatic, and detoxifier. Similar therapeutic applications were also reported in many other countries [95,107].

## 4. Conclusions and Perspectives

The purpose of the present study was to, for the first time, collect and organize a standardized dataset containing detailed information available from the literature about Italian folk medicinal remedies for human health produced using wild and cultivated plants. Based on the large amount of literature data collected from 75 analyzed scientific papers, the present study confirmed that traditional medicine was widely diffused in the past all along the peninsula and it is still practiced in many territories by both people who are involved and not involved in agricultural practices. The most cited species (citation index close to 1) were diffused and used in many Italian regions while others had only local application. Thirteen plant species were identified as being the most relevant in Italy for medicinal use: *Malva sylvestris* L., *Sambucus nigra* L., *Urtica dioica* L., *Matricaria chamomilla* L., *Cynodon dactylon* (L.) Pers., *Rosmarinus officinalis* L., *Borago officinalis* L., *Cichorium intybus* L., *Olea europaea* L., *Hypericum perforatum* L., *Salvia officinalis* L., *Laurus nobilis* L., and *Foeniculum vulgare* Mill.

A wide range of phytotherapeutic uses were recorded, mainly concerning disorders of the digestive system, skin-ears-eyes-hair, systemic system, and genito-urinary system. In general, the most common traditional medicinal remedies were prepared by simple and easy methods, such as an infusion or decoction of the plant parts, followed by ingestion or direct external topic application. Most of the identified plants with medicinal applications were also used as food ingredients [9], therefore proving the tight relation between daily diet and human health present in the traditional folk knowledge of the different Italian regions.

This review, by collecting and organizing the knowledge of Italian wild and cultivated medicinal flora, is, therefore, a starting point for further specific bioactivity and bioprospecting studies aimed at the formulation of therapeutic drugs that are more environmentally sustainable and respectful of plant biodiversity.

## Figures and Tables

**Table 1:** Reviewed papers according to the distribution in Italian regions.

Italian Regions	Papers
Abruzzo	[21,22,23,24]
Basilicata	[21,25,26,27,28,29,30,31]
Calabria	[21,32,33,34,35]
Campania	[19,36,37,38,39,40,41,42,43,44,45]
Emilia-Romagna	[46,47,48]
Friuli-Venezia Giulia	[49,50];
Lazio	[21,23,51,52]
Liguria	[53,54,55]
Lombardia	[5,56,57,58,59,60]
Marche	[21,61,62,63]
Molise	[21,23,24,64,65];
Piemonte	[66,67,68,69]
Puglia	[21]
Sardegna	[70,71,72,73,74,75,76,77]
Sicilia	[20,76,78,79,80,81,82]
Toscana	[47,83,84,85,86]
Trentino-Alto Adige	[13]
Umbria	[21,87,88]
Valle d’Aosta	[14]
Whole Italy	[89,90]

**Table 2:** Pathological groups and principal therapeutic applications of cited plant species. The classification used was based on the standardized descriptors reported by Cook [91].

Pathological Groups	Principal Therapeutic Uses and Treated Diseases	n. of Plant Species	n. of Papers
**1—Digestive system diseases**	Astringent, anti-abdominal pain, anti-diarrheic, carminative, eupeptic, cholagogue, anti-parasitic and mycotic diseases, lenitive of teeth and mouth inflammations, oral disinfectants	705	74
**2—Skin-ears-eyes-hair diseases and wounds**	Lenitive of insect stings, emollient, cicatrizing, curative of dermatitis, skin infections, aesthetic problems, ear infections, ophthalmic inflammations, chilblains, wounds, burns, bruises	661	72
**3—Systemic diseases**	Antimicrobial, anti-phlogistic, anti-pyretic, tonic, reconstituent, depurative, analgesic, coadjuvant, cleansing, diaphoretic	531	70
**4—Genito-urinary system diseases**	Menstruation regulator, abortifacient, galactagogue, oxytocic, lenitive of urinary diseases and cystitis, lithotripter, depurative	522	75
**5—Respiratory system diseases**	Anti-asthma, cold, cough, bronchitis, expectorant, pulmonary diseases, inflammation of the respiratory system	499	69
**6—Nervous system diseases**	Sedative, calming effects, drowsiness, antidepressive, memory booster, headache, insomnia, epilepsy	348	70
**7—Cardio-circulatory system diseases**	Action on the cardiac rhythm, vasoconstrictor/vasodilatory, pressure regulation, blood depurative, cardiopathies, varicose veins	333	72
**8—Muscular-skeletal diseases**	Lumbago, arthritis pains, effect on calcium and bone metabolism, sprains, rheumatism, swollen feet and legs	301	68
**9—Metabolic diseases**	Mineral integrator, vitamin deficiency, diabetes, antigout, cytotoxic, hypo/hypercholesterolemic	223	46

## Data Availability

The data presented in this study are available in Supplementary Table S1.

## Supplementary Materials

The following supporting information can be downloaded at: https://www.mdpi.com/article/10.3390/plants11152041/s1, Table S1: Dataset of Italian *wild and cultivated* plant species used for medicinal purposes.
