# Supplementary material for: Ethnobotanical Review and Dataset Compiling on Wild and Cultivated Plants Traditionally Used as Medicinal Remedies in Italy
Source: Plants (Basel). 2022 Aug 4;11(15):2041. doi: 10.3390/plants11152041 (PMC9370752; doi:10.3390/plants11152041)
Supplement: Supplementary file 1 [file plants-11-02041-s001.zip › plants-1830896-supplementary.pdf]

Table S1. Dataset of Italian wild and cultivated plant species used for medicinal purpose. w: wild, c: cultivated, w/c: wild and cultivated; CI: citation index, UI: use index; cit.n., number of citations; nd: not determined. Pathological groups: 1) digestive system diseases, 2) skin-ears-eyes-hair diseases and wounds, 3) systemic diseases, 4) genito-urinary system diseases, 5) respiratory system diseases, 6) nervous system diseases, 7) cardio-circulatory system diseases, 8) muscular-skeletal diseases, 9) metabolic diseases.

Species	Family	Vernacular name	w/c	CI	UI	Papers	cit. n.	Pathological groups								
								1	2	3	4	5	6	7	8	9
Abies alba Mill.	Pinaceae	Abete bianco	w	0.12	0.89	Lokar and Poldrini, 1988; Pieroni, 2000; Camangi et al, 2003; Passalacqua et al, 2007; Vitalini et al, 2009; Di Sanzo et al, 2013; Cornara et al, 2014; Bellia and Pieroni, 2015; Petalka et al, 2020	9	X	X	X		X	X	X	X	X
Abies sp.pl.	Pinaceae	-	nd	0.01	0.11	Mattalia et al, 2019	1		X							
Acacia karroo Hayne	Fabaceae	-	c	0.01	0.22	Palmese et al, 2001	1			X	X					
Acanthus mollis L.	Acanthaceae	Acanto	c	0.05	0.44	Bruni et al, 1997; Leto et al, 2012; Tuttolomondo et al, 2014; Gargano et al, 2018	4	X	X					X	X	
Acer campestre L.	Sapindaceae	Acero campestre	w	0.01	0.11	Mautone et al, 2019	1				X					
Acer neapolitanum (Ten.) Gmas.	Sapindaceae	Acero napoletano	w	0.01	0.11	Guarrera and Lucia, 2007	1		X							
Acer opalus subsp. *obtusatum* (Waldst. & Kit. ex Willd.) Gams	Sapindaceae	Opalo	w	0.01	0.11	Leporatti et al, 1985b	1	X								
Acer pseudoplatanus L.	Sapindaceae	Acero di monte	w	0.01	0.11	Bellia and Pieroni, 2015	1					X				
Achillea ageratum L.	Compositae	Millefoglio	w	0.01	0.67	Guarino et al, 2008	1	X	X	X	X	X		X		
Achillea atrata L.	Compositae	Millefoglio del calcare	w	0.01	0.56	Petalka et al, 2020	1		X		X	X	X		X	
Achillea clavenae L.	Compositae	Millefolio	w	0.03	0.67	Lokar and Poldrini, 1988; Petalka et al, 2020	2	X	X		X	X	X		X	
Achillea collina (Becker ex Rchb.f.) Heimerl	Compositae	Millefoglio	w	0.05	0.67	Guarrera et al, 2005; Guarino et al, 2008; Cornara et al, 2014; Lucchetti et al, 2019	4	X	X		X	X		X	X	
Achillea erba-rotta All.	Compositae	Millefoglio erba rotta	w	0.08	0.78	Pieroni and Giusti, 2009; Mattalia et al, 2012; Cornara et al, 2014; Bellia and Pieroni, 2015; Fontefrancesco and Pieroni, 2020; Danna et al, 2022	6	X	X	X	X	X	X	X		
Achillea erba-rotta subsp. *moschata* (Wulfen) I.Richardson	Compositae	Millefoglio erba rotta	w	0.01	0.11	Bruschi et al, 2019	1	X								
Achillea ligustica All.	Compositae		w	0.09	0.67	Bruni et al, 1997; Palmese et al, 2001; Signorini et al, 2009; Leto et al, 2012; Cornara et al, 2014; Tuttolomondo et al, 2014; Tuttolomondo et al, 2014b	7	X	X	X	X		X		X	
Achillea millefolium L.	Compositae	Millefoglio	w	0.41	1.00	Lokar and Poldrini, 1988; Bruni et al, 1997; Pieroni, 2000; Leporatti and Ivancheva, 2003; Pieroni et al, 2004a; Pieroni et al, 2004b; Pieroni and Quave, 2005; Menale et al, 2006; Passalacqua et al, 2007; Gonzalez-Tejero et al, 2008; Guarino et al, 2008; Pieroni and Giusti, 2009; Vitalini et al, 2009;	30	X	X	X	X	X	X	X	X	X

						Vitalini et al, 2012; Di Novella et al, 2013; Cornara et al, 2014; Dei Cas et al, 2015; Vitalini et al, 2015; Fortini et al, 2016; Ranfa and Bodesmo, 2017; Bruschi et al, 2019; Maruca et al, 2019; Mautone et al, 2019; Bottoni et al, 2020; Fontefrancesco and Pieroni, 2020; Mattalia et al, 2020a; Petalka et al, 2020; Galuzzo et al, 2021; Mattalia et al, 2021; Danna et al, 2022										
Achillea moschata Wulfen	Compositae	Millefoglio del granito	w	0.05	0.89	Vitalini et al, 2012; Dei Cas et al, 2015; Vitalini et al, 2015; Petalka et al, 2020; Danna et al, 2022	4	X	X	X	X	X	X	X		X
Achillea nana L.	Compositae	Millefoglio nano	w	0.01	0.22	Vitalini et al, 2015	1	X				X				
Achillea nobilis L.	Compositae	-	w	0.01	0.33	Guarino et al, 2008	1	X			X	X				
Achillea sp.pl.	Compositae	-	nd	0.01	0.22	Idolo et al, 2010	1		X		X					
Aconitum napellus L.	Ranunculaceae	Aconito	w	0.03	0.22	Vitalini et al, 2015; Petalka et al, 2020	2	X		X						
Acorus calamus L.	Acoraceae	Calamo aromatico	w	0.01	0.33	Leporatti and Ivancheva, 2003	1	X	X		X					
Actaea spicata L.	Ranunculaceae	Barba di capra	w	0.01	0.11	Guarino et al, 2008	1	X								
Adiantum capillus-veneris L.	Pteridaceae	Capelvenere	w	0.31	0.78	Lokar and Poldrini, 1988; Bruni et al, 1997; Leporatti and Corradi, 2001; Palmese et al, 2001; Loi et al, 2004; Maccioni et al, 2004; Loi et al, 2005; Pieroni and Quave, 2005; De Natale and Pollio, 2007; Passalacqua et al, 2007; Guarino et al, 2008; Leporatti and Ghedira, 2009; Idolo et al, 2010; Savo et al, 2011; Leto et al, 2012; Di Sanzo et al, 2013; Cornara et al, 2014; Tuttolomondo et al, 2014; Tuttolomondo et al, 2014b; Menale et al, 2016; Petalka et al, 2020; Menale et al, 2021	23	X	X	X	X	X	X	X		
Adiantum hispidulum Sw.	Pteridaceae	-	w	0.01	0.22	Fontefrancesco and Pieroni, 2020	1				X			X		
Adonis annua L.	Ranunculaceae	Adonide	w	0.03	0.22	Bruni et al, 1997; Guarino et al, 2008	2				X			X		
Adonis annua subsp. *cupaniana* (Guss.) C.Steinb.	Ranunculaceae	Adonide	w	0.01	0.11	Lucchetti et al, 2019	1				X					
Aegopodium podagraria L.	Apiaceae	Girardina silvestre	w	0.03	0.67	Guarino et al, 2008; Petalka et al, 2020	2	X	X	X		X			X	X
Aesculus hippocastanum L.	Sapindaceae	Ippocastano	c	0.14	0.67	Pieroni, 2000; Camangi et al, 2003; Leporatti and Ivancheva, 2003; Pieroni et al, 2004b; Guarrera et al, 2005; Gonzalez-Tejero et al, 2008; Vitalini et al, 2015; Menale et al, 2016; Mattalia et al, 2019; Petalka et al, 2020	10	X	X	X		X		X		X
Aethusa cyspenapium L.	Apiaceae	-	w	0.01	0.22	Pieroni, 2000	1	X		X						
Agave americana L.	Asparagaceae	Agave	w/c	0.12	0.22	Uncini Manganelli and Tomei, 1999; Cornara et al, 2009; Savo et al, 2011; Leto et al, 2012; Montesano et al, 2012; Tuttolomondo et al, 2014; Motti and Motti, 2017; Gargano et al, 2018; Menale et al, 2021	9		X						X	

Agrimonia eupatoria L.	Rosaceae	Agrimonia comune	w	0.20	0.78	Lokar and Poldrini, 1988; Leporatti and Ivancheva, 2003; Pieroni et al, 2004a; Pieroni and Quave, 2005; De Natale and Pollio, 2007; Guarino et al, 2008; Vitalini et al, 2009; Di Novella et al, 2013; Di Sanzo et al, 2013; Cornara et al, 2014; Tuttolomondo et al, 2014; Guarrera et al, 2015; Vitalini et al, 2015; Lucchetti et al, 2019; Petalka et al, 2020	15	X	X	X	X	X	X	X		
Agropyron junceum (L.) P.Beauv.	Poaceae	-	w	0.01	0.22	Loi et al, 2005	1			X	X					
Agropyron repens (L.) P.Beauv.	Poaceae	-	w	0.01	0.33	Menale and Muoio, 2014	1	X	X			X				
Agrostemma githago L.	Caryophyllaceae	Gittaione	w	0.01	0.33	Guarino et al, 2008	1		X		X	X				
Ailanthus altissima (Mill.) Swingle	Simaroubaceae	Ailanto	w	0.04	0.33	Fortini et al, 2016; Menale et al, 2016; Lucchetti et al, 2019	3	X	X			X				
Ajuga iva (L.) Schreb.	Lamiaceae	Iva moscata	w	0.04	0.44	Bruni et al, 1997; Guarino et al, 2008; Leporatti and Ghedira, 2009	3	X	X	X	X					
Ajuga reptans L.	Lamiaceae	Iva comune	w	0.11	0.78	Lokar and Poldrini, 1988; Gonzalez-Tejero et al, 2008; Guarino et al, 2008; Di Novella et al, 2013; Maruca et al, 2019; Mautone et al, 2019; Petalka et al, 2020; Galuzzo et al, 2021	8	X	X	X	X	X		X		X
Alcea rosea L.	Malvaceae	Malvone	w/c	0.04	0.56	Bruni et al, 1997; Menale et al, 2016; Motti and Motti, 2017	3	X	X	X	X	X				
Alchemilla alpina L.	Rosaceae	Alchemilla alpestre	w	0.03	0.78	Vitalini et al, 2012; Petalka et al, 2020	2	X	X	X	X	X	X			X
Alchemilla vulgaris L.	Rosaceae	Alchemilla comune	w	0.08	0.78	Lokar and Poldrini, 1988; Leporatti and Ivancheva, 2003; Mattalia et al, 2012; Vitalini et al, 2012; Cornara et al, 2014; Dei Cas et al, 2015	6	X	X	X	X		X	X		X
Alchemilla xanthochlora Rothm.	Rosaceae	-	w	0.07	0.78	Bellia and Pieroni, 2015; Vitalini et al, 2015; Fontefrancesco and Pieroni, 2020; Petalka et al, 2020; Danna et al, 2022	5		X	X	X	X	X		X	X
Alliaria petiolata (M.Bieb.) Cavara and Grande	Brassicaceae	Alliaria	w	0.07	0.67	Guarino et al, 2008; Di Novella et al, 2013; Ranfa and Bodesmo, 2017; Lucchetti et al, 2019; Petalka et al, 2020	5	X	X	X	X	X	X			
Allium ampeloprasum L.	Amaryllidaceae	Porraccio	w	0.03	0.22	Bellia and Pieroni, 2015; Danna et al, 2022	2	X			X					
Allium cepa L.	Amaryllidaceae	Cipolla	c	0.54	1.00	Lokar and Poldrini, 1988; Bruni et al, 1997; Uncini Manganelli and Tomei, 1999; Pieroni, 2000; Ballero et al, 2001; Palmese et al, 2001; Camangi et al, 2003; Leporatti and Ivancheva, 2003; Loi et al, 2004; Pieroni et al, 2004a; Pieroni et al, 2004b; Guarrera et al, 2005; Loi et al, 2005; Pieroni and Quave, 2005; Scherrer et al, 2005; Guarrera and Lucia, 2007; Passalacqua et al, 2007; Gonzalez-Tejero et al, 2008; Guarino et al, 2008; Cornara et al,	40	X	X	X	X	X	X	X	X	X

						2009; Leporatti and Ghedira, 2009; Motti et al, 2009; Idolo et al, 2010; Savo et al, 2011; Montesano et al, 2012; Cornara et al, 2014; Menale and Muoio, 2014; Tuttolomondo et al, 2014a; Tuttolomondo et al, 2014b; Dei Cas et al, 2015; Guarrera et al, 2015; Vitalini et al, 2015; Fortini et al, 2016; Menale et al, 2016; Motti and Motti, 2017; Bruschi et al, 2019; Lucchetti et al, 2019; Fontefrancesco and Pieroni, 2020; Menale et al, 2021; Danna et al, 2022										
Allium neapolitanum Cirillo	Amaryllidaceae	Aglio napoletano	w	0.03	0.22	Lucchetti et al, 2019; Motti et al, 2020	2	X						X		
Allium nigrum L.	Amaryllidaceae	-	w	0.03	0.33	Loi et al, 2005; Leto et al, 2012	2	X	X	X						
Allium porrum L.	Amaryllidaceae	Porro	c	0.01	0.11	Idolo et al, 2010	1	X								
Allium roseum L.	Amaryllidaceae	Aglio rosato	w	0.03	0.11	Loi et al, 2005; Motti et al, 2020	2	X								
Allium sativum L.	Amaryllidaceae	Aglio	w	0.59	1.00	Lokar and Poldrini, 1988; Uncini Manganelli and Tomei, 1999; Pieroni, 2000; Ballero et al, 2001; Leporatti and Corradi, 2001; Palmese et al, 2001; Camangi et al, 2003; Leporatti and Ivancheva, 2003; Loi et al, 2004; Maccioni et al, 2004; Pieroni et al, 2004a; Pieroni et al, 2004b; Guarrera et al, 2005; Guarrera et al, 2005a; Loi et al, 2005; Pieroni and Quave, 2005; Scherrer et al, 2005; Menale et al, 2006; Passalacqua et al, 2007; Gonzalez-Tejero et al, 2008; Guarino et al, 2008; Maxia et al, 2008; Cornara et al, 2009; Leporatti and Ghedira, 2009; Motti et al, 2009; Idolo et al, 2010; Savo et al, 2011; Montesano et al, 2012; Cornara et al, 2014; Menale and Muoio, 2014; Tuttolomondo et al, 2014b; Bellia and Pieroni, 2015; Dei Cas et al, 2015; Guarrera et al, 2015; Vitalini et al, 2015; Fortini et al, 2016; Menale et al, 2016; Motti and Motti, 2017; Bruschi et al, 2019; Lucchetti et al, 2019; Mautone et al, 2019; Fontefrancesco and Pieroni, 2020; Menale et al, 2021; Danna et al, 2022	44	X	X	X	X	X	X	X	X	X
Allium schoenoprasum L.	Amaryllidaceae	-	w	0.09	1.00	Lokar and Poldrini, 1988; Mattalia et al, 2012; Vitalini et al, 2012; Cornara et al, 2014; Dei Cas et al, 2015; Vitalini et al, 2015; Petalka et al, 2020	7	X	X	X	X	X	X	X	X	X
Allium subhirsutum L.	Amaryllidaceae	-	w	0.03	0.33	Loi et al, 2004; Leto et al, 2012	2	X	X	X						
Allium triquetrum L.	Amaryllidaceae	Aglio triquetro	w	0.03	0.33	Ranfa and Bodesmo, 2017; Motti et al, 2020	2			X			X	X		
Allium ursinum L.	Amaryllidaceae	Aglio orsino	w	0.12	0.78	Leporatti and Ivancheva, 2003; Guarino et al, 2008; Idolo et al, 2010; Fortini et al, 2016; Ranfa and Bodesmo, 2017; Bruschi et al, 2019; Mattalia et al, 2019; Petalka et al, 2020; Mattalia et al, 2021	9	X	X	X	X	X	X	X		
Allium victorialis L.	Amaryllidaceae	Aglio serpentino	w	0.01	0.44	Petalka et al, 2020	1			X	X	X	X			

Alnus cordata (Loisel.) Duby	Betulaceae	Ontano napoletano	w	0.03	0.22	Menale and Muoio, 2014; Maruca et al, 2019	2	X	X							
Alnus glutinosa (L.) Gaertn.	Betulaceae	Ontano nero	w	0.09	0.44	Bruni et al, 1997; Leporatti and Ivancheva, 2003; Loi et al, 2004; Loi et al, 2005; Cornara et al, 2009; Leporatti and Ghedira, 2009; Di Novella et al, 2013	7	X	X	X	X					
Alnus incana (L.) Moench	Betulaceae	Ontano bianco	w	0.01	0.11	Fontefrancesco and Pieroni, 2020	1								X	
Aloe arborescens Mill.	Xanthorrhoeaceae	Aloe	c	0.05	0.22	Scherrer et al, 2005; Menale et al, 2016; Motti and Motti, 2017; Mautone et al, 2019	4		X						X	
Aloe maculata All.	Xanthorrhoeaceae	Aloe	c	0.01	0.11	Menale et al, 2016	1		X							
Aloe sp.pl.	Xanthorrhoeaceae	-	c	0.01	0.33	Menale et al, 2021	1	X	X			X				
Aloe vera (L.) Burm. f.	Xanthorrhoeaceae	Aloe	c	0.08	0.44	Palmese et al, 2001; Passalacqua et al, 2007; Savo et al, 2011; Tuttolomondo et al, 2014b; Fortini et al, 2016; Bruschi et al, 2019	6	X	X	X					X	
Aloysia citriodora Palau	Verbenaceae	Erba limoncina	c	0.23	0.67	Bruni et al, 1997; Uncini Manganelli and Tomei, 1999; Pieroni, 2000; Pieroni et al, 2004b; Menale et al, 2006; Gonzalez-Tejero et al, 2008; Maxia et al, 2008; Cornara et al, 2009; Savo et al, 2011; Cornara et al, 2014; Tuttolomondo et al, 2014b; Bellia and Pieroni, 2015; Mautone et al, 2019; Fontefrancesco and Pieroni, 2020; Mattalia et al, 2020a; Menale et al, 2021; Danna et al, 2022	17	X	X	X	X	X	X			
Althaea cannabina L.	Malvaceae	-	w	0.01	0.11	Mautone et al, 2019	1		X							
Althaea hirsuta L.	Malvaceae	-	w	0.03	0.44	Guarino et al, 2008; Geraci et al, 2018	2	X	X		X	X				
Althaea officinalis L.	Malvaceae	Altea comune	w	0.18	0.89	Lokar and Poldrini, 1988; Ballero et al, 2001; Leporatti and Ivancheva, 2003; Pieroni et al, 2004b; Menale et al, 2006; De Natale and Pollio, 2007; Gonzalez-Tejero et al, 2008; Di Novella et al, 2013; Menale and Muoio, 2014; Tuttolomondo et al, 2014a; Geraci et al, 2018; Fontefrancesco and Pieroni, 2020; Petalka et al, 2020	13	X	X	X	X	X	X	X		X
Amaranthus retroflexus L.	Amaranthaceae	Amaranto	w	0.03	0.22	De Natale and Pollio, 2007; Savo et al, 2011	2					X			X	
Ammi majus L.	Apiaceae	-	w	0.04	0.56	Lokar and Poldrini, 1988; Guarino et al, 2008; Galuzzo et al, 2021	3	X	X	X	X	X				
Ammi visnaga (L.) Lam.	Apiaceae	-	w	0.01	0.33	Leporatti and Ghedira, 2009	1	X			X			X		
Anacamptis morio (L.) R.M.Bateman, Pridgeon & M.W.Chase	Orchidaceae	Orchide minore	w	0.01	0.11	Petalka et al, 2020	1		X							
Anacyclus clavatus (Desf.) Pers.	Compositae	-	w	0.03	0.22	Leto et al, 2012; Tuttolomondo et al, 2014	2		X				X			

Anagallis arvensis L.	Primulaceae	-	w	0.07	0.67	Leporatti and Ivancheva, 2003; Loi et al, 2005; Guarino et al, 2008; Leto et al, 2012; Lucchetti et al, 2019	5	X	X	X	X	X			X	
Anagallis arvensis L. subsp. *arvensis*	Primulaceae	-	w	0.01	0.11	De Natale and Pollio, 2007	1					X				
Anchusa officinalis L.	Boraginaceae	Buglossa	w	0.01	0.22	Vitalini et al, 2015	1		X		X					
Anchusa undulata L. subsp. *hybrida* (Ten.) Bég.	Boraginaceae	Buglossa ondulata	w	0.03	0.22	Fortini et al, 2016; Motti and Motti, 2017	2	X						X		
Anemone hortensis L.	Ranunculaceae	Anemone viola	w	0.01	0.33	Guarino et al, 2008	1		X	X					X	
Anemone nemorosa L.	Ranunculaceae	Anemone dei boschi	w	0.01	0.33	Guarino et al, 2008	1		X	X					X	
Anemone pulsatilla L.	Ranunculaceae	-	w	0.01	0.22	Leporatti and Ivancheva, 2003	1						X	X		
Anemone vernalis L.	Ranunculaceae	-	w	0.01	0.44	Petalka et al, 2020	1		X		X	X				X
Anethum graveolens L.	Apiaceae	Aneto	c	0.08	0.44	Leporatti and Ivancheva, 2003; Menale et al, 2006; Leporatti and Ghedira, 2009; Vitalini et al, 2009; Fortini et al, 2016; Galuzzo et al, 2021	6	X			X	X		X		
Angelica archangelica L.	Apiaceae	-	w	0.04	0.33	Leporatti and Ivancheva, 2003; Guarino et al, 2008; Fontefrancesco and Pieroni, 2020	3	X	X	X						
Angelica sylvestris L.	Apiaceae	Angelica selvatica	w	0.12	0.67	Lokar and Poldrini, 1988; Guarino et al, 2008; Idolo et al, 2010; Vitalini et al, 2012; Cornara et al, 2014; Vitalini et al, 2015; Mautone et al, 2019; Fontefrancesco and Pieroni, 2020; Petalka et al, 2020	9	X	X			X	X	X		X
Antennaria dioica L.	Compositae	Sempiterni di monte	w	0.04	0.67	Lokar and Poldrini, 1988; Pieroni and Giusti, 2009; Petalka et al, 2020	3	X	X	X	X	X	X			
Anthemis arvensis L.	Compositae	Falsa camomilla	w	0.05	0.44	Uncini Manganelli and Tomei, 1999; Loi et al, 2005; Tuttolomondo et al, 2014; Galuzzo et al, 2021	4	X	X	X			X			
Anthemis arvensis L. subsp. *incrassata* (Loisel.) Nyman	Compositae	Falsa camomilla	w	0.01	0.56	Menale et al, 2016	1	X	X		X	X	X			
Anthemis cotula L.	Compositae	Camomilla fetida	w	0.01	0.11	Guarino et al, 2008	1			X						
Anthoxanthum odoratum L.	Poaceae	Paleo odoroso	w	0.01	0.11	Guarino et al, 2008	1			X						
Anthriscus cerefolium (L.) Hoffm.	Apiaceae	Cerfoglio	w/c	0.03	0.44	Guarino et al, 2008; Galuzzo et al, 2021	2	X	X	X	X					
Anthriscus sylvestris (L.) Hoffm.	Apiaceae	Cerfoglio	w	0.04	0.67	Guarino et al, 2008; Leporatti and Ghedira, 2009; Petalka et al, 2020	3		X	X	X	X			X	X
Anthyllis vulneraria L.	Fabaceae	Vulneraria	w	0.05	0.22	Lokar and Poldrini, 1988; Guarino et al, 2008; Vitalini et al, 2015; Petalka et al, 2020	4		X	X						
Antirrhinum majus L.	Plantaginaceae	Bocca di leone	w	0.01	0.44	Guarino et al, 2008	1	X	X	X		X				
Apium graveolens L.	Apiaceae	Sedano	c	0.22	0.78	Leporatti et al, 1985a; Leporatti et al, 1985b; Lokar and Poldrini, 1988; Leporatti and Ivancheva, 2003; Loi et al, 2005; Passalacqua et al, 2007; Guarino et al, 2008; Leporatti and Ghedira, 2009; Menale and	16	X	X	X	X	X			X	X

						Muoio, 2014; Dei Cas et al, 2015; Guarrera et al, 2015; Menale et al, 2016; Lucchetti et al, 2019; Mautone et al, 2019; Mattalia et al, 2020b; Galuzzo et al, 2021										
Apium nodiflorum (L.) Lag.	Apiaceae	Sedano acquatico	w/c	0.09	0.33	Pieroni, 2000; Loi et al, 2004; Guarino et al, 2008; Cornara et al, 2009; Leto et al, 2012; Tuttolomondo et al, 2014; Vitalini et al, 2015	7	X		X	X					
Aquilegia einseleana F.W.Schultz	Ranunculaceae	Aquilegia	w	0.01	0.11	Petalka et al, 2020	1						X			
Aquilegia vulgaris L.	Ranunculaceae	Aquilegia	w	0.04	0.67	Leporatti et al, 1985b; Lokar and Poldrini, 1988; Guarino et al, 2008	3	X	X	X	X		X			X
Arbutus unedo L.	Ericaceae	Corbezzolo	w	0.27	0.89	Bruni et al, 1997; Uncini Manganelli and Tomei, 1999; Ballero et al, 2001; Palmese et al, 2001; Loi et al, 2004; Maccioni et al, 2004; Loi et al, 2005; Passalacqua et al, 2007; Maxia et al, 2008; Cornara et al, 2009; Leonti et al, 2009; Leporatti and Ghedira, 2009; Signorini et al, 2009; Leto et al, 2012; Tuttolomondo et al, 2014; Menale et al, 2016; Maruca et al, 2019; Mattalia et al, 2019; Mautone et al, 2019; Menale et al, 2021	20	X	X	X	X	X	X	X	X	
Arctium lappa L.	Compositae	Bardana maggiore	w	0.31	0.89	Lokar and Poldrini, 1988; Pieroni, 2000; Leporatti and Ivancheva, 2003; Loi et al, 2004; Menale et al, 2006; Gonzalez-Tejero et al, 2008; Guarino et al, 2008; Vitalini et al, 2009; Idolo et al, 2010; Mattalia et al, 2012; Vitalini et al, 2012; Di Novella et al, 2013; Bellia and Pieroni, 2015; Dei Cas et al, 2015; Vitalini et al, 2015; Menale et al, 2016; Maruca et al, 2019; Bruschi et al, 2019; Mattalia et al, 2019; Fontefrancesco and Pieroni, 2020; Mattalia et al, 2020a; Petalka et al, 2020; Danna et al, 2022	23	X	X	X	X	X		X	X	X
Arctium minus (Hill) Bernh.	Compositae	Bardana minore	w	0.03	0.78	Guarino et al, 2008; Lucchetti et al, 2019	2	X	X	X	X	X		X	X	
Arctium nemorosum Lej.	Compositae	-	w	0.04	0.67	Leporatti et al, 1985b; Leporatti and Ivancheva, 2003; Cornara et al, 2014	3	X	X	X	X	X		X		
Arctium sp.pl.	Compositae	-	nd	0.01	0.22	Leporatti and Corradi, 2001	1		X		X					
Arctostaphylos uva-ursi (L.) Spreng.	Ericaceae	Uva orsina	w	0.11	0.67	Lokar and Poldrini, 1988; Idolo et al, 2010; Bellia and Pieroni, 2015; Dei Cas et al, 2015; Vitalini et al, 2015; Bruschi et al, 2019; Petalka et al, 2020; Danna et al, 2022	8	X	X	X	X	X				X
Arisarum vulgare O. Targ.Tozz.	Araceae	Arisaro	w	0.03	0.22	De Natale and Pollio, 2007; Tuttolomondo et al, 2014b	2		X						X	
Aristolochia clematitis L.	Aristolochiaceae	Aristolochia	w	0.04	0.44	Lokar and Poldrini, 1988; Leporatti and Ivancheva, 2003; Danna et al, 2022	3		X	X	X				X	
Aristolochia fontanesii Boiss. & Reut.	Aristolochiaceae	Aristolochia	w	0.01	0.11	Leto et al, 2012	1									X

Armoracia rusticana P. Gaertn, B.Mey & Scherb.	Brassicaceae	-	w	0.08	0.44	Lokar and Poldrini, 1988; Leporatti and Ivancheva, 2003; Pieroni et al, 2004a; Pieroni and Quave, 2005; Vitalini et al, 2015	6	X	X	X					X	
Arnica montana L.	Compositae	Arnica	w	0.19	1.00	Lokar and Poldrini, 1988; Pieroni and Giusti, 2009; Vitalini et al, 2009; Mattalia et al, 2012; Vitalini et al, 2012; Cornara et al, 2014; Bellia and Pieroni, 2015; Dei Cas et al, 2015; Vitalini et al, 2015; Bruschi et al, 2019; Fontefrancesco and Pieroni, 2020; Mattalia et al, 2020a; Petalka et al, 2020; Danna et al, 2022	14	X	X	X	X	X	X	X	X	X
Artemisia abrotanum L.	Compositae	-	w	0.03	0.33	Di Novella et al, 2013; Tuttolomondo et al, 2014a	2	X	X	X						
Artemisia absinthium L.	Compositae	Assenzio	w	0.32	1.00	Leporatti et al, 1985b; Lokar and Poldrini, 1988; Pieroni, 2000; Leporatti and Corradi, 2001; Camangi et al, 2003; Leporatti and Ivancheva, 2003; Maccioni et al, 2004; Menale et al, 2006; Guarino et al, 2008; Leporatti and Ghedira, 2009; Pieroni and Giusti, 2009; Vitalini et al, 2009; Mattalia et al, 2012; Vitalini et al, 2012; Cornara et al, 2014; Menale and Muoio, 2014; Bellia and Pieroni, 2015; Dei Cas et al, 2015; Vitalini et al, 2015; Mautone et al, 2019; Mattalia et al, 2020a; Petalka et al, 2020; Galuzzo et al, 2021; Danna et al, 2022	24	X	X	X	X	X	X	X	X	X
Artemisia alba Turra	Compositae	Assenzio maschio	w	0.04	0.22	Leto et al, 2012; Tuttolomondo et al, 2014a; Tuttolomondo et al, 2014b	3		X	X						
Artemisia arborescens (Vaill.) L.	Compositae	-	w	0.20	1.00	Bruni et al, 1997; Ballero et al, 2001; Palmese et al, 2001; Loi et al, 2004; Loi et al, 2005; De Natale and Pollio, 2007; Maxia et al, 2008; Leonti et al, 2009; Signorini et al, 2009; Savo et al, 2011; Leto et al, 2012; Tuttolomondo et al, 2014; Tuttolomondo et al, 2014a; Tuttolomondo et al, 2014b; Gargano et al, 2018	15	X	X	X	X	X	X	X	X	X
Artemisia campestris L.	Compositae	Assenzio selvatico	w	0.01	0.33	Leporatti and Ghedira, 2009	1		X	X						X
Artemisia campestris L. subsp. *glutinosa* (Besser) Batt.	Compositae	Assenzio selvatico	w	0.01	0.33	Guarino et al, 2008	1		X	X			X			
Artemisia campestris L. subsp. *variabilis* (Ten.) Greuter	Compositae	Assenzio selvatico	w	0.03	0.22	Menale et al, 2016; Menale et al, 2021	2	X						X		
Artemisia coerulescens L.	Compositae	-	w	0.01	0.22	Lokar and Poldrini, 1988	1	X		X						
Artemisia genipi Weber ex Stechm.	Compositae	Assenzio genipì	w	0.09	0.44	Pieroni and Giusti, 2009; Vitalini et al, 2012; Bellia and Pieroni, 2015; Dei Cas et al, 2015; Vitalini et al,	7	X	X	X		X				

						2015; Fontefrancesco and Pieroni, 2020; Danna et al, 2022										
Artemisia glacialis L.	Compositae	Assenzio alpino	w	0.03	0.33	Mattalia et al, 2012; Bellia and Pieroni, 2015	2	X		X		X				
Artemisia maritima L.	Compositae	-	w	0.01	0.11	Mattalia et al, 2020a	1	X								
Artemisia umbelliformis Lam.	Compositae	-	w	0.11	0.67	Pieroni and Giusti, 2009; Mattalia et al, 2012; Vitalini et al, 2012; Bellia and Pieroni, 2015; Dei Cas et al, 2015; Vitalini et al, 2015; Petalka et al, 2020; Danna et al, 2022	8	X		X	X	X	X	X		
Artemisia verlotiorum Lamotte	Compositae	-	w	0.01	0.11	Menale et al, 2016	1							X		
Artemisia vulgaris L.	Compositae	Assenzio selvatico	w	0.15	0.78	Leporatti and Ivancheva, 2003; Guarino et al, 2008; Pieroni and Giusti, 2009; Vitalini et al, 2009; Menale and Muoio, 2014; Bellia and Pieroni, 2015; Vitalini et al, 2015; Bruschi et al, 2019; Lucchetti et al, 2019; Petalka et al, 2020; Danna et al, 2022	11	X	X	X	X	X	X	X		
Arum cylindraceum Gasp.	Araceae	-	w	0.01	0.56	Guarino et al, 2008	1	X			X	X			X	X
Arum italicum Mill.	Araceae	Gigaro chiaro	w	0.22	0.56	Bruni et al, 1997; Uncini Manganelli and Tomei, 1999; Camangi et al, 2003; Pieroni et al, 2004a; Pieroni and Quave, 2005; De Natale and Pollio, 2007; Gonzalez-Tejero et al, 2008; Leporatti and Ghedira, 2009; Leto et al, 2012; Montesano et al, 2012; Tuttolomondo et al, 2014; Tuttolomondo et al, 2014b; Fortini et al, 2016; Lucchetti et al, 2019; Maruca et al, 2019; Mautone et al, 2019	16	X	X	X	X				X	
Arum maculatum L.	Araceae	Gigaro scuro	w	0.01	0.44	Leporatti and Ivancheva, 2003	1	X				X			X	X
Arum pictum L.f.	Araceae	-	w	0.03	0.33	Bruni et al, 1997; Loi et al, 2004	2		X		X				X	
Aruncus dioicus (Walter) Fernald	Rosaceae	-	w	0.01	0.44	Lokar and Poldrini, 1988	1		X	X	X				X	
Arundo donax L.	Poaceae	Canna domestica	w	0.31	0.67	Bruni et al, 1997; Ballero et al, 2001; Leporatti and Corradi, 2001; Palmese et al, 2001; Pieroni et al, 2004a; Loi et al, 2005; Pieroni and Quave, 2005; Menale et al, 2006; Passalacqua et al, 2007; Guarino et al, 2008; Maxia et al, 2008; Leporatti and Ghedira, 2009; Leto et al, 2012; Montesano et al, 2012; Tuttolomondo et al, 2014; Tuttolomondo et al, 2014a; Tuttolomondo et al, 2014b; Menale et al, 2016; Lucchetti et al, 2019; Maruca et al, 2019; Mattalia et al, 2019; Mautone et al, 2019; Menale et al, 2021	23	X	X	X	X	X		X		
Asarum europaeum L.	Aristolochiaceae	Baccaro	w	0.01	0.22	Leporatti and Ivancheva, 2003	1	X				X				
Asclepias curassavica L.	Apocynaceae	-	w	0.01	0.11	Tuttolomondo et al, 2014a	1		X							
Asparagus acutifolius L.	Asparagaceae	Asparago selvatico	w	0.27	0.67	Lokar and Poldrini, 1988; Bruni et al, 1997; Pieroni, 2000; Maccioni et al, 2004; Pieroni et al, 2004a; Loi	20	X		X	X	X		X		X

						et al, 2005; Pieroni and Quave, 2005; Pieroni et al, 2005; Scherrer et al, 2005; Guarino et al, 2008; Signorini et al, 2009; Tuttolomondo et al, 2014; Tuttolomondo et al, 2014a; Tuttolomondo et al, 2014b; Sansanelli et al, 2017; Gargano et al, 2018; Lucchetti et al, 2019; Maruca et al, 2019; Mautone et al, 2019; Menale et al, 2021										
Asparagus albus L.	Asparagaceae	-	w	0.03	0.11	Tuttolomondo et al, 2014; Gargano et al, 2018	2				X					
Asparagus officinalis L.	Asparagaceae	Asparago coltivato	c	0.16	0.78	Leporatti et al, 1985b; Lokar and Poldrini, 1988; Leporatti and Corradi, 2001; Leporatti and Ivancheva, 2003; Menale et al, 2006; Guarino et al, 2008; Cornara et al, 2009; Leporatti and Ghedira, 2009; Di Novella et al, 2013; Menale and Muoio, 2014; Vitalini et al, 2015; Petalka et al, 2020	12	X		X	X	X		X	X	X
Asparagus sp.pl.	Asparagaceae	-	nd	0.01	0.22	Idolo et al, 2010	1				X	X				
Asparagus tenuifolius Lam.	Asparagaceae	Asparago coltivato	w/c	0.04	0.22	Uncini Manganelli and Tomei, 1999; Vitalini et al, 2009; Vitalini et al, 2012	3			X	X					
Asphodeline lutea (L.) Rchb.	Xanthorrhoeaceae	-	w	0.01	0.11	Tuttolomondo et al, 2014a	1		X							
Asphodelus albus Mill.	Xanthorrhoeaceae	Asfodelo	w	0.03	0.33	Leporatti and Ghedira, 2009; Di Novella et al, 2013	2		X			X			X	
Asphodelus cerasifer Gay	Xanthorrhoeaceae	-	w	0.01	0.11	Tuttolomondo et al, 2014	1		X							
Asphodelus fistulosus L.	Xanthorrhoeaceae	Asfodelo pustolato	w	0.01	0.22	Leonti et al, 2009	1		X			X				
Asphodelus macrocarpus Parl.	Xanthorrhoeaceae	Asfodelo montano	w	0.01	0.33	Guarino et al, 2008	1		X	X	X					
Asphodelus microcarpus Saltzm. & Viv.	Xanthorrhoeaceae	Asfodelo	w	0.08	0.67	Palmese et al, 2001; Loi et al, 2004; Loi et al, 2005; Leporatti and Ghedira, 2009; Signorini et al, 2009; Tuttolomondo et al, 2014b	6	X	X	X	X	X			X	
Asphodelus ramosus L.	Xanthorrhoeaceae	Asfodelo	w	0.07	0.22	Ballero et al, 2001; Maxia et al, 2008; Leonti et al, 2009; Leto et al, 2012; Tuttolomondo et al, 2014	5		X			X				
Asplenium adiantum-nigrum L.	Aspleniaceae	Adianto nero	w	0.04	0.67	Palmese et al, 2001; Guarino et al, 2008; Di Novella et al, 2013	3	X	X	X	X	X			X	
Asplenium ruta-muraria L.	Aspleniaceae	Ruta muraria	w	0.01	0.56	Guarino et al, 2008	1	X	X		X				X	X
Asplenium scolopendrium L.	Aspleniaceae	Lingua cervina	w	0.03	0.44	Lokar and Poldrini, 1988; Di Sanzo et al, 2013	2	X	X		X	X				
Asplenium septentrionale (L.) Hoffm.	Aspleniaceae	Asplenio settentrionale	w	0.04	0.44	Dei Cas et al, 2015; Vitalini et al, 2015; Petalka et al, 2020	3	X		X	X					X
Asplenium trichomanes L.	Aspleniaceae	Tricomane	w	0.08	0.67	Lokar and Poldrini, 1988; Camangi et al, 2003; Leporatti and Ivancheva, 2003; Pieroni and Quave, 2005; De Natale and Pollio, 2007; Guarino et al, 2008	6	X	X	X	X	X			X	
Aster alpinus L.	Compositae	Astro alpino	w	0.01	0.11	Vitalini et al, 2015	1		X							

Astracantha sicula (Biv.) Greuter	Fabaceae	-	w	0.01	0.22	Tuttolomondo et al, 2014a	1		X		X					
Astragalus glycyphyllos L.	Fabaceae	Astragalo	w	0.04	0.67	Lokar and Poldrini, 1988; Leporatti and Ivancheva, 2003; Guarino et al, 2008	3	X			X	X		X	X	X
Astragalus monspessulanus L.	Fabaceae	Astragalo	w	0.01	0.11	Guarino et al, 2008	1				X					
Athamanta cretensis L.	Apiaceae	Atamanta comune	w	0.01	0.22	Petalka et al, 2020	1				X	X				
Athamanta sicula L.	Apiaceae	-	w	0.01	0.11	Tuttolomondo et al, 2014	1				X					
Athyrium filix-femina (L.) Roth	Athyriaceae	Felce femmina	w	0.01	0.11	Di Novella et al, 2013	1					X				
Atropa bella-donna L.	Solanaceae	Belladonna	w	0.18	0.89	Leporatti et al, 1985b; Leporatti and Ivancheva, 2003; De Natale and Pollio, 2007; Guarino et al, 2008; Idolo et al, 2010; Leto et al, 2012; Mattalia et al, 2012; Fortini et al, 2016; Bruschi et al, 2019; Mattalia et al, 2019; Mattalia et al, 2020b; Petalka et al, 2020; Mattalia et al, 2021	13	X	X	X		X	X	X	X	X
Avena barbata Pott ex Link	Poaceae	Avena selvatica	w	0.01	0.44	Guarino et al, 2008	1					X	X	X	X	
Avena fatua L.	Poaceae	Avena selvatica	w	0.07	0.67	Guarino et al, 2008; Tuttolomondo et al, 2014; Tuttolomondo et al, 2014a; Tuttolomondo et al, 2014b; Gargano et al, 2018	5		X	X		X	X	X	X	
Avena sativa L.	Poaceae	Avena	c	0.22	0.89	Bruni et al, 1997; Leporatti and Ivancheva, 2003; Pieroni et al, 2004a; Pieroni et al, 2004b; Pieroni and Quave, 2005; Guarino et al, 2008; Maxia et al, 2008; Leporatti and Ghedira, 2009; Montesano et al, 2012; Di Novella et al, 2013; Cornara et al, 2014; Menale and Muoio, 2014; Menale et al, 2016; Motti and Motti, 2017; Geraci et al, 2018; Lucchetti et al, 2019	16	X	X	X	X	X	X	X	X	
Ballota nigra L.	Lamiaceae	Cimiciotta comune	w	0.07	0.89	Pieroni, 2000; Pieroni et al, 2004a; Pieroni and Quave, 2005; Guarino et al, 2008; Galuzzo et al, 2021	5	X	X	X	X	X	X	X	X	
Ballota nigra subsp. *ruderalis* (Sw.) Briq.	Lamiaceae	Cimiciotta comune	w	0.01	0.11	Signorini et al, 2009	1	X								
Barbarea vulgaris R.Br.	Brassicaceae	Erba di santa Barbara	w	0.01	0.22	Guarino et al, 2008	1			X						X
Bartsia alpina L.	Orobanchaceae	Bartsia	w	0.04	0.33	Bellia and Pieroni, 2015; Dei Cas et al, 2015; Petalka et al, 2020	3		X			X			X	
Beckwithia glacialis (L.) Á.Löve & D.Löve	Ranunculaceae	-	w	0.01	0.22	Petalka et al, 2020	1						X			X
Bellis perennis L.	Compositae	Pratolina	w	0.15	0.89	Lokar and Poldrini, 1988; Leporatti and Corradi, 2001; Leporatti and Ivancheva, 2003; Passalacqua et al, 2007; Guarino et al, 2008; Cornara et al, 2014; Ranfa and Bodesmo, 2017; Lucchetti et al, 2019; Mautone et al, 2019; Petalka et al, 2020; Galuzzo et al, 2021	11	X	X	X	X	X	X	X	X	

Berberis vulgaris L.	Berberidaceae	Crespino comune	w	0.09	0.78	Lokar and Poldrini, 1988; Leporatti and Ivancheva, 2003; Idolo et al, 2010; Dei Cas et al, 2015; Vitalini et al, 2015; Petalka et al, 2020; Danna et al, 2022	7	X	X	X	X	X	X	X		
Beta vulgaris L.	Amaranthaceae	Bietola	c	0.26	0.67	Uncini Manganelli and Tomei, 1999; Pieroni, 2000; Palmese et al, 2001; Maccioni et al, 2004; Pieroni et al, 2004b; Guarrera et al, 2005; Loi et al, 2005; Menale et al, 2006; Cornara et al, 2009; Signorini et al, 2009; Cornara et al, 2014; Tuttolomondo et al, 2014; Bellia and Pieroni, 2015; Dei Cas et al, 2015; Vitalini et al, 2015; Menale et al, 2016; Motti and Motti, 2017; Menale et al, 2021; Danna et al, 2022	19	X	X	X	X	X			X	
Betula pendula Roth	Betulaceae	Betulla	w	0.18	1.00	Lokar and Poldrini, 1988; Pieroni, 2000; Leporatti and Ivancheva, 2003; Pieroni et al, 2004b; Di Novella et al, 2013; Dei Cas et al, 2015; Vitalini et al, 2015; Menale et al, 2016; Bruschi et al, 2019; Fontefrancesco and Pieroni, 2020; Mattalia et al, 2020a; Petalka et al, 2020; Danna et al, 2022	13	X	X	X	X	X	X	X	X	X
Borago officinalis L.	Boraginaceae	Borraggine	w	0.64	1.00	Leporatti et al, 1985b; Lokar and Poldrini, 1988; Bruni et al, 1997; Uncini Manganelli and Tomei, 1999; Pieroni, 2000; Ballero et al, 2001; Leporatti and Corradi, 2001; Leporatti and Ivancheva, 2003; Loi et al, 2004; Maccioni et al, 2004; Pieroni et al, 2004a; Pieroni et al, 2004b; Loi et al, 2005; Pieroni and Quave, 2005; Pieroni et al, 2005; Scherrer et al, 2005; De Natale and Pollio, 2007; Passalacqua et al, 2007; Guarino et al, 2008; Cornara et al, 2009; Leonti et al, 2009; Leporatti and Ghedira, 2009; Motti et al, 2009; Idolo et al, 2010; Savo et al, 2011; Mattalia et al, 2012; Montesano et al, 2012; Leto et al, 2012; Vitalini et al, 2012; Di Sanzo et al, 2013; Cornara et al, 2014; Tuttolomondo et al, 2014; Tuttolomondo et al, 2014a; Tuttolomondo et al, 2014b; Bellia and Pieroni, 2015; Vitalini et al, 2015; Fortini et al, 2016; Motti and Motti, 2017; Geraci et al, 2018; Bruschi et al, 2019; Lucchetti et al, 2019; Maruca et al, 2019; Mattalia et al, 2019; Mautone et al, 2019; Mattalia et al, 2020b; Petalka et al, 2020; Menale et al, 2021	47	X	X	X	X	X	X	X	X	X
Botrychium lunaria (L.) Sw.	Ophioglossaceae	Botrichio	w	0.03	0.44	Bellia and Pieroni, 2015; Petalka et al, 2020	2		X		X	X		X		
Brassica cretica Lam.	Brassicaceae	-	w	0.01	0.22	Motti and Motti, 2017	1	X	X							
Brassica montana Pourr.	Brassicaceae	-	w	0.01	0.00	Guarino et al, 2008	1				X					
Brassica napus L.	Brassicaceae	Senape bianca	c	0.04	0.22	Ballero et al, 2001; Guarino et al, 2008; Leporatti and Ghedira, 2009	3		X	X						

Brassica nigra (L.) K.Koch	Brassicaceae	-	c	0.05	0.56	Camangi et al, 2003; Leporatti and Ivancheva, 2003; Guarino et al, 2008; Idolo et al, 2010	4		X	X		X		X	X	
Brassica oleracea L.	Brassicaceae	Cavolo nero	c	0.43	0.89	Uncini Manganelli and Tomei, 1999; Pieroni, 2000; Camangi et al, 2003; Leporatti and Ivancheva, 2003; Maccioni et al, 2004; Pieroni et al, 2004a; Pieroni et al, 2004b; Guarrera et al, 2005; Pieroni and Quave, 2005; Passalacqua et al, 2007; Gonzalez-Tejero et al, 2008; Guarino et al, 2008; Motti et al, 2009; Idolo et al, 2010; Savo et al, 2011; Montesano et al, 2012; Cornara et al, 2014; Menale and Muoio, 2014; Tuttolomondo et al, 2014b; Bellia and Pieroni, 2015; Dei Cas et al, 2015; Guarrera et al, 2015; Fortini et al, 2016; Menale et al, 2016; Motti and Motti, 2017; Bruschi et al, 2019; Lucchetti et al, 2019; Maruca et al, 2019; Mautone et al, 2019; Fontefrancesco and Pieroni, 2020; Menale et al, 2021; Danna et al, 2022	32	X	X	X	X	X	X		X	X
Brassica rapa L.	Brassicaceae	Rapa	c	0.09	0.67	Guarrera et al, 2005; Guarino et al, 2008; Bellia and Pieroni, 2015; Menale et al, 2016; Motti and Motti, 2017; Petalka et al, 2020; Danna et al, 2022	7	X	X	X	X	X	X			
Brassica rupestris Raf.	Brassicaceae	-	w	0.03	0.22	Tuttolomondo et al, 2014a; Tuttolomondo et al, 2014b	2	X							X	
Brassica sp.pl.	Brassicaceae	-	nd	0.01	0.33	Cornara et al, 2009	1	X		X					X	
Bryonia alba L.	Cucurbitaceae	-	w	0.01	0.44	Leporatti and Ivancheva, 2003;	1	X	X		X				X	
Bryonia dioica Jacq.	Cucurbitaceae	Brionia comune	w	0.11	0.56	Lokar and Poldrini, 1988; Pieroni, 2000; Leporatti and Corradi, 2001; Leporatti and Ivancheva, 2003; Pieroni et al, 2004b; Guarino et al, 2008; Leporatti and Ghedira, 2009; Menale and Muoio, 2014	8	X	X		X	X			X	
Buglossoides purpurocaerulea (L.) I. M. Johnst.	Boraginaceae	Erba perla	w	0.01	0.11	Guarino et al, 2008	1	X								
Bunias erucago L.	Brassicaceae	Cascellone	w	0.05	0.22	Pieroni, 2000; Guarino et al, 2008; Tuttolomondo et al, 2014a; Ranfa and Bodesmo, 2017	4	X			X					
Buphthalmum salicifolium L.	Compositae	-	w	0.01	0.11	Lokar and Poldrini, 1988	1		X							
Bupleurum rotundifolium L.	Apiaceae	Buplero	w	0.01	0.22	Guarino et al, 2008	1	X	X							
Buxus sempervirens L.	Buxaceae	Bosso	w/c	0.05	0.33	Leporatti et al, 1985b; Guarino et al, 2008; Leporatti and Ghedira, 2009; Di Novella et al, 2013	4	X		X					X	
Calendula arvensis M.Bieb.	Compositae	Calendula	w	0.11	0.56	Loi et al, 2005; Passalacqua et al, 2007; Leporatti and Ghedira, 2009; Signorini et al, 2009; Tuttolomondo et al, 2014b; Fortini et al, 2016; Motti and Motti, 2017; Ranfa and Bodesmo, 2017	8	X	X	X	X				X	
Calendula officinalis L.	Compositae	Calendula	w/c	0.26	0.89	Lokar and Poldrini, 1988; Leporatti and Corradi, 2001; Leporatti and Ivancheva, 2003; Pieroni et al, 2004b; Guarrera et al, 2005; Menale et al, 2006;	19	X	X	X	X	X	X	X	X	

						Gonzalez-Tejero et al, 2008; Guarino et al, 2008; Vitalini et al, 2012; Cornara et al, 2014; Tuttolomondo et al, 2014a; Bellia and Pieroni, 2015; Dei Cas et al, 2015; Vitalini et al, 2015; Fortini et al, 2016; Menale et al, 2016; Bruschi et al, 2019; Lucchetti et al, 2019; Mattalia et al, 2020a										
Calendula sp.pl.	Compositae	-	nd	0.03	0.56	Idolo et al, 2010; Galuzzo et al, 2021	2	X	X		X		X	X		
Calicotome villosa (Poir.) Link	Fabaceae	Ginestra villosa	w	0.01	0.11	Loi et al, 2004	1		X							
Calluna vulgaris (L.) Hull	Ericaceae	Brugo	w	0.03	0.67	Fontefrancesco and Pieroni, 2020; Petalka et al, 2020	2	X			X	X	X	X		X
Calystegia sepium (L.) R. Br.	Convolvulaceae	Campanella bianca	w	0.07	0.44	Passalacqua et al, 2007; Di Sanzo et al, 2013; Menale and Muoio, 2014; Lucchetti et al, 2019; Mautone et al, 2019	5	X	X					X	X	
Calystegia silvatica (Kit.) Griseb.	Convolvulaceae	Campanella bianca	w	0.03	0.22	Passalacqua et al, 2007; Savo et al, 2011	2	X	X							
Camelina sativa (L.) Crantz	Brassicaceae	-	w	0.01	0.22	Leporatti and Ghedira, 2009	1	X	X							
Camellia japonica L.	Theaceae	-	w	0.01	0.11	Menale et al, 2021	1	X								
Camellia sinensis (L.) Kuntze	Theaceae	Tè	w	0.01	0.44	Bruschi et al, 2019	1	X	X			X	X			
Campanula dichotoma L.	Campanulaceae	-	w	0.01	0.11	Scherrer et al, 2005	1					X				
Campanula fragilis subsp. *fragilis*	Campanulaceae	Campanula delicata	w	0.01	0.11	Savo et al, 2011	1			X						
Campanula patula L.	Campanulaceae	-	w	0.01	0.33	Petalka et al, 2020	1						X	X	X	
Campanula rapunculus L.	Campanulaceae	-	w	0.03	0.33	Pieroni, 2000; Ranfa and Bodesmo, 2017	2	X	X		X					
Campanula rotundifolia L.	Campanulaceae	Campanula	w	0.01	0.33	Petalka et al, 2020	1						X	X	X	
Campanula scheuchzeri Vill.	Campanulaceae	Campanula di Scheurzer	w	0.01	0.11	Vitalini et al, 2015	1		X							
Cannabis sativa L.	Cannabaceae	Canapa	c	0.01	0.33	Guarrera et al, 2005	1	X	X			X				
Capparis orientalis Veill.	Capparaceae	Cappero	w	0.01	0.11	Menale et al, 2021	1		X							
Capparis spinosa L.	Capparaceae	Cappero	w/c	0.04	0.67	Cornara et al, 2009; Leporatti and Ghedira, 2009; Tuttolomondo et al, 2014a	3	X	X		X		X		X	X
Capsella bursa-pastoris (L.) Medik.	Brassicaceae	Borsa del pastore	w	0.27	0.89	Lokar and Poldrini, 1988; Leporatti and Ivancheva, 2003; Loi et al, 2005; De Natale and Pollio, 2007; Guarino et al, 2008; Leporatti and Ghedira, 2009; Signorini et al, 2009; Idolo et al, 2010; Leto et al, 2012; Vitalini et al, 2012; Cornara et al, 2014; Tuttolomondo et al, 2014; Bellia and Pieroni, 2015; Dei Cas et al, 2015; Vitalini et al, 2015; Motti and	20	X	X	X	X	X	X	X		X

						Motti, 2017; Lucchetti et al, 2019; Mautone et al, 2019; Petalka et al, 2020; Danna et al, 2022										
Capsella rubella Reut.	Brassicaceae	Borsa del pastore	w	0.03	0.33	Guarino et al, 2008; Motti and Motti, 2017	2	X			X			X		
Capsicum annuum L.	Solanaceae	Pepperoncino	c	0.24	1.00	Uncini Manganelli and Tomei, 1999; Pieroni, 2000; Leporatti and Ivancheva, 2003; Pieroni et al, 2004a; Pieroni et al, 2004b; Pieroni and Quave, 2005; Menale et al, 2006; Passalacqua et al, 2007; Guarino et al, 2008; Leporatti and Ghedira, 2009; Montesano et al, 2012; Di Novella et al, 2013; Menale and Muoio, 2014; Menale et al, 2016; Motti and Motti, 2017; Bruschi et al, 2019; Mautone et al, 2019; Menale et al, 2021	18	X	X	X	X	X	X	X	X	X
Cardamine amara L.	Brassicaceae	Billeri amara	w	0.01	0.33	Vitalini et al, 2015	1	X		X					X	
Cardamine enneaphyllos (L.) Crantz	Brassicaceae	-	w	0.03	0.44	Guarino et al, 2008; Petalka et al, 2020	2	X			X	X				X
Carduus defloratus L.	Compositae	Cardo dentellato	w	0.01	0.11	Danna et al, 2022	1				X					
Carduus nutans L.	Compositae	-	w	0.01	0.22	Tuttolomondo et al, 2014a	1	X			X					
Carduus pycnocephalus subsp. *albidus* (M.Bieb.) Kazmi	Compositae	Saettone	w	0.01	0.11	Guarino et al, 2008	1				X					
Carex sp.pl.	Cyperaceae	-	nd	0.01	0.11	Menale et al, 2016	1	X								
Carica papaya L.	Caricaceae	Papaia	c	0.01	0.11	Pieroni et al, 2004b	1		X							
Carlina acanthifolia All.	Compositae	-	w	0.04	0.33	Leporatti and Ivancheva, 2003; Fortini et al, 2016; Fontefrancesco and Pieroni, 2020	3	X	X		X					
Carlina acanthifolia subsp. *utzka* (Hacq.) Meusel & Kästner	Compositae	-	w	0.01	0.11	Di Novella et al, 2013	1	X								
Carlina acaulis L.	Compositae	Fiore di carta	w	0.08	0.78	Leporatti et al, 1985b; Lokar and Poldrini, 1988; Menale et al, 2006; Vitalini et al, 2009; Vitalini et al, 2015; Petalka et al, 2020; Danna et al, 2022	6	X	X	X	X	X	X		X	
Carlina acaulis L. subsp. *caulescens* (Lam.) Schübl. & G.Martens	Compositae	Fiore di carta	w	0.03	0.44	Guarino et al, 2008; Fortini et al, 2016	2	X	X	X	X					
Carlina corymbosa L.	Compositae	Fiore di carta	w	0.01	0.11	Savo et al, 2011	1	X								
Carpobrotus acinaciformis (L.) L.Bolus	Aizoaceae	Fico degli ottentotti	w	0.03	0.22	Motti et al, 2009; Menale et al, 2021	2	X						X		
Carpobrotus edulis (L.) N.E.Br.	Aizoaceae	Fico degli ottentotti	w	0.03	0.22	Scherrer et al, 2005; Motti and Motti, 2017	2	X	X							
Carthamus lanatus L.	Compositae	-	w	0.01	0.44	Guarino et al, 2008	1	X		X	X		X			
Carthamus tinctorius L.	Compositae	Cartamo	w/c	0.03	0.22	Galuzzo et al, 2021; Leporatti and Ghedira, 2009	2	X							X	

Carum carvi L.	Apiaceae		w	0.19	0.78	Lokar and Poldrini, 1988; Leporatti and Ivancheva, 2003; Leporatti and Ghedira, 2009; Pieroni and Giusti, 2009; Vitalini et al, 2009; Vitalini et al, 2012; Bellia and Pieroni, 2015; Dei Cas et al, 2015; Vitalini et al, 2015; Fontefrancesco and Pieroni, 2020; Mattalia et al, 2020a; Petalka et al, 2020; Galuzzo et al, 2021; Danna et al, 2022	14	X	X		X	X	X	X		X
Castanea sativa Mill.	Fagaceae	Castagno	w/c	0.19	0.67	Pieroni, 2000; Ballero et al, 2001; Camangi et al, 2003; Pieroni et al, 2004b; Guarrera et al, 2005; Scherrer et al, 2005; Passalacqua et al, 2007; Cornara et al, 2009; Idolo et al, 2010; Vitalini et al, 2015; Bruschi et al, 2019; Maruca et al, 2019; Petalka et al, 2020; Menale et al, 2021	14	X	X	X		X	X	X		
Caucalis platycarpos L.	Apiaceae		w	0.01	0.22	Guarino et al, 2008	1	X			X					
Celtis australis L.	Cannabaceae	Spacca sassi	w	0.04	0.22	Guarino et al, 2008; Di Novella et al, 2013; Lucchetti et al, 2019	3	X		X						
Centaurea benedicta (L.) L.	Compositae	Fiordaliso benedetto	w	0.01	0.33	Leporatti and Ivancheva, 2003	1	X		X		X				
Centaurea calcitrapa L.	Compositae	-	w	0.11	0.67	Guarino et al, 2008; Maxia et al, 2008; Leporatti and Ghedira, 2009; Leto et al, 2012; Tuttolomondo et al, 2014; Tuttolomondo et al, 2014b; Geraci et al, 2018; Galuzzo et al, 2021	8	X	X	X	X				X	X
Centaurea cyanus L.	Compositae	Fiordaliso	w	0.01	0.44	Leporatti and Ivancheva, 2003	1	X	X	X		X				
Centaurea jacea subsp. *gaudinii* (Boiss. & Reut.) Gremli	Compositae	Fordaliso stoppione	w	0.01	0.22	Guarrera and Lucia, 2007	1				X	X				
Centaurea nigrescens Willd.	Compositae	Fiordaliso nerastro	w	0.01	0.11	Guarino et al, 2008	1	X								
Centaurea pratensis Thuill.	Compositae	fiordaliso	w	0.03	0.11	Leporatti et al, 1985b; Guarrera and Lucia, 2007	2	X								
Centaurea solstitialis subsp. *schouwii* (DC.) Gugler	Compositae	-	w	0.01	0.11	Leto et al, 2012	1				X					
Centaurea sp.pl.	Compositae	-	nd	0.01	0.11	Mattalia et al, 2020a	1	X								
Centaurium erythraea Rafn	Gentianaceae	Centauro eritreo	w	0.26	0.89	Leporatti et al, 1985b; Lokar and Poldrini, 1988; Ballero et al, 2001; Camangi et al, 2003; Leporatti and Ivancheva, 2003; Maccioni et al, 2004; Pieroni et al, 2004a; Pieroni and Quave, 2005; Guarrera and Lucia, 2007; Guarino et al, 2008; Leporatti and Ghedira, 2009; Idolo et al, 2010; Di Novella et al, 2013; Di Sanzo et al, 2013; Cornara et al, 2014; Tuttolomondo et al, 2014b; Fortini et al, 2016; Maruca et al, 2019; Petalka et al, 2020	19	X	X	X	X	X	X	X		X

Centaurium pulchellum (Sw.) Druce	Gentianaceae	-	w	0.03	0.44	Guarino et al, 2008; Leporatti and Ghedira, 2009	2	X	X	X			X			
Centranthus ruber (L.) DC.	Caprifoliaceae	Camarezza minore	w	0.11	0.67	Passalacqua et al, 2007; Guarino et al, 2008; Cornara et al, 2009; Savo et al, 2011; Menale and Muoio, 2014; Tuttolomondo et al, 2014a; Menale et al, 2016; Mautone et al, 2019	8	X		X	X	X	X		X	
Cerastium fontanum Baumg.	Caryophyllaceae	-	w	0.01	0.11	Petalka et al, 2020	1								X	
Ceratonia siliqua L.	Fabaceae	Carrubo	w	0.20	0.44	Ballero et al, 2001; Pieroni et al, 2004b; Guarrera et al, 2005; Scherrer et al, 2005; Guarrera and Lucia, 2007; Guarino et al, 2008; Leporatti and Ghedira, 2009; Motti et al, 2009; Savo et al, 2011; Montesano et al, 2012; Menale and Muoio, 2014; Menale et al, 2016; Motti and Motti, 2017; Mautone et al, 2019; Menale et al, 2021	15	X	X	X		X				
Cerinthe major L.	Boraginaceae	Erba vajola	w	0.04	0.44	Loi et al, 2005; Guarino et al, 2008; Tuttolomondo et al, 2014	3	X	X	X			X			
Cestrum parqui Benth.	Solanaceae	-	w	0.04	0.56	Guarino et al, 2008; Menale and Muoio, 2014; Menale et al, 2016	3		X	X		X	X		X	
Ceterach officinarum Willd.	Aspleniaceae	Cedracca	w	0.26	0.89	Pieroni, 2000; Palmese et al, 2001; Maccioni et al, 2004; Pieroni et al, 2004a; Guarrera et al, 2005; Pieroni and Quave, 2005; De Natale and Pollio, 2007; Passalacqua et al, 2007; Guarino et al, 2008; Savo et al, 2011; Leto et al, 2012; Di Novella et al, 2013; Di Sanzo et al, 2013; Tuttolomondo et al, 2014; Tuttolomondo et al, 2014a; Tuttolomondo et al, 2014b; Gargano et al, 2018; Mattalia et al, 2019; Mautone et al, 2019	19	X	X	X	X	X		X	X	X
Chamaemelum nobilis All.	Compositae	-	w	0.01	0.11	Mattalia et al, 2019	1						X			
Chelidonium majus L.	Papaveraceae	Erba da porri	w	0.53	0.89	Lokar and Poldrini, 1988; Bruni et al, 1997; Pieroni, 2000; Leporatti and Corradi, 2001; Camangi et al, 2003; Leporatti and Ivancheva, 2003; Loi et al, 2004; Maccioni et al, 2004; Pieroni et al, 2004b; Guarrera et al, 2005; Menale et al, 2006; Guarrera and Lucia, 2007; Passalacqua et al, 2007; Gonzalez-Tejero et al, 2008; Guarino et al, 2008; Pieroni and Giusti, 2009; Vitalini et al, 2009; Idolo et al, 2010; Savo et al, 2011; Mattalia et al, 2012; Vitalini et al, 2012; Di Novella et al, 2013; Cornara et al, 2014; Menale and Muoio, 2014; Bellia and Pieroni, 2015; Dei Cas et al, 2015; Vitalini et al, 2015; Fortini et al, 2016; Menale et al, 2016; Motti and Motti, 2017; Bruschi et al, 2019; Lucchetti et al, 2019; Mautone et al, 2019; Bottoni et al, 2020; Mattalia et al, 2020a;	39	X	X	X	X	X	X		X	X

						Mattalia et al, 2020b; Petalka et al, 2020; Mattalia et al, 2021; Danna et al, 2022									
Chenopodium album L.	Amaranthaceae	Farinaccio bianco	w	0.04	0.44	Dei Cas et al, 2015; De Natale and Pollio, 2007; Maruca et al, 2019	3	X	X			X			X	
Chenopodium bonus-henricus L.	Amaranthaceae	-	w	0.15	0.67	Lokar and Poldrini, 1988; Pieroni, 2000; Leporatti and Ivancheva, 2003; Menale et al, 2006; Pieroni and Giusti, 2009; Vitalini et al, 2012; Di Novella et al, 2013; Dei Cas et al, 2015; Vitalini et al, 2015; Lucchetti et al, 2019; Petalka et al, 2020	11	X	X	X		X	X	X		
Chondrilla juncea L.	Compositae	-	w	0.03	0.22	Tuttolomondo et al, 2014a; Ranfa and Bodesmo, 2017	2	X	X							
Cicer arietinum L.	Fabaceae	Cece	c	0.01	0.11	Guarino et al, 2008	1	X								
Cichorium endivia L.	Compositae	Indivia	c	0.05	0.44	Cornara et al, 2009; Menale et al, 2016; Geraci et al, 2018; Menale et al, 2021	4	X		X	X			X		
Cichorium intybus L.	Compositae	Cicoria	w	0.64	1.00	Leporatti et al, 1985b; Lokar and Poldrini, 1988; Bruni et al, 1997; Uncini Manganelli and Tomei, 1999; Pieroni, 2000; Camangi et al, 2003; Leporatti and Ivancheva, 2003; Pieroni et al, 2004a; Guarrera et al, 2005; Loi et al, 2005; Pieroni and Quave, 2005; Pieroni et al, 2005; Scherrer et al, 2005; Menale et al, 2006; Passalacqua et al, 2007; Gonzalez-Tejero et al, 2008; Guarino et al, 2008; Maxia et al, 2008; Cornara et al, 2009; Leonti et al, 2009; Leporatti and Ghedira, 2009; Signorini et al, 2009; Vitalini et al, 2009; Idolo et al, 2010; Leto et al, 2012; Mattalia et al, 2012; Montesano et al, 2012; Menale and Muoio, 2014; Tuttolomondo et al, 2014; Tuttolomondo et al, 2014a; Tuttolomondo et al, 2014b; Dei Cas et al, 2015; Vitalini et al, 2015; Fortini et al, 2016; Menale et al, 2016; Motti and Motti, 2017; Sansanelli et al, 2017; Geraci et al, 2018; Bruschi et al, 2019; Lucchetti et al, 2019; Maruca et al, 2019; Mattalia et al, 2019; Mautone et al, 2019; Motti et al, 2020; Petalka et al, 2020; Galuzzo et al, 2021; Menale et al, 2021	47	X	X	X	X	X	X	X	X	X
Cinnamomum camphora (L.) J.Presl	Lauraceae	Canfora	c	0.01	0.11	Bruschi et al, 2019	1					X				
Cirsium arvense (L.) Scop.	Compositae	Cardo campestre	w	0.05	0.56	Pieroni et al, 2004b; Guarrera and Lucia, 2007; Guarino et al, 2008; Lucchetti et al, 2019	4	X	X	X	X	X				
Cistus incanus L.	Cistaceae	Cisto maggiore	w	0.01	0.11	Bruni et al, 1997	1			X						
Cistus monspeliensis L.	Cistaceae	Cisto di Montpellier	w	0.01	0.22	Uncini Manganelli and Tomei, 1999	1		X	X						
Cistus salviifolius L.	Cistaceae	Cisto salvia	w	0.03	0.11	Leto et al, 2012; Tuttolomondo et al, 2014b	2		X							
Cistus sp.pl.	Cistaceae	-	nd	0.03	0.33	Ballero et al, 2001; Loi et al, 2005	2		X	X					X	

Citrullus colocynthis (L.) Schrad.	Cucurbitaceae	-	w	0.01	0.22	Leporatti and Ghedira, 2009	1	X			X					
Citrus aurantium L.	Rutaceae	-	c	0.08	0.56	Palmese et al, 2001; Pieroni et al, 2004b; Passalacqua et al, 2007; Gonzalez-Tejero et al, 2008; Menale et al, 2016; Menale et al, 2021	6	X	X	X		X	X			
Citrus limon (L.) Osbeck	Rutaceae	limone	c	0.38	1.00	Leporatti et al, 1985a; Uncini Manganelli and Tomei, 1999; Pieroni, 2000; Ballero et al, 2001; Palmese et al, 2001; Camangi et al, 2003; Pieroni et al, 2004b; Guarrera et al, 2005; Guarrera et al, 2005a; Loi et al, 2005; Pieroni and Quave, 2005; Scherrer et al, 2005; Passalacqua et al, 2007; Maxia et al, 2008; Motti et al, 2009; Idolo et al, 2010; Savo et al, 2011; Montesano et al, 2012; Cornara et al, 2014; Menale and Muoio, 2014; Guarrera et al, 2015; Menale et al, 2016; Motti and Motti, 2017; Gargano et al, 2018; Bruschi et al, 2019; Lucchetti et al, 2019; Mautone et al, 2019; Menale et al, 2021	28	X	X	X	X	X	X	X	X	X
Citrus nobilis Lour.	Rutaceae	Arancio	c	0.01	0.22	Savo et al, 2011	1	X				X				
Citrus paradisi Macfad.	Rutaceae	Pompelmo	c	0.01	0.11	Menale et al, 2016	1							X		
Citrus reticulata Blanco	Rutaceae	Madarino	c	0.05	0.56	Uncini Manganelli and Tomei, 1999; Palmese et al, 2001; Menale et al, 2016; Motti and Motti, 2017	4	X	X	X		X	X			
Citrus sinensis (L.) Osbeck	Rutaceae	Limone	c	0.14	0.67	Palmese et al, 2001; Pieroni et al, 2004a; Pieroni et al, 2004b; Pieroni and Quave, 2005; Motti et al, 2009; Savo et al, 2011; Montesano et al, 2012; Fortini et al, 2016; Menale et al, 2016; Bruschi et al, 2019	10	X	X	X	X	X	X			
Clematis cirrhosa L.	Ranunculaceae	-	w	0.01	0.44	Palmese et al, 2001	1	X	X	X					X	
Clematis flammula L.	Ranunculaceae	Clematide fiammola	w	0.04	0.67	Leporatti and Corradi, 2001; Guarino et al, 2008; Leporatti and Ghedira, 2009	3	X	X				X	X	X	X
Clematis sp.pl.	Ranunculaceae	-	w	0.01	0.44	Ballero et al, 2001	1	X	X	X					X	
Clematis vitalba L.	Ranunculaceae	Vitalba	w	0.24	0.89	Lokar and Poldrini, 1988; Uncini Manganelli and Tomei, 1999; Pieroni, 2000; Leporatti and Corradi, 2001; Camangi et al, 2003; Leporatti and Ivancheva, 2003; Loi et al, 2004; Maccioni et al, 2004; Pieroni et al, 2004a; Pieroni et al, 2004b; Guarrera et al, 2005; Pieroni and Quave, 2005; Guarino et al, 2008; Leporatti and Ghedira, 2009; Montesano et al, 2012; Di Sanzo et al, 2013; Lucchetti et al, 2019; Mattalia et al, 2019; Mattalia et al, 2020b	18	X	X	X	X		X	X	X	X
Clinopodium alpinum (L.) Kuntze	Lamiaceae	Clinopodio alpino	w	0.01	0.22	Petalka et al, 2020	1					X		X		
Clinopodium menthifolium (Host) Stace	Lamiaceae	-	w	0.01	0.11	Guarino et al, 2008	1	X								

Clinopodium nepeta (L.) Kuntze	Lamiaceae	Erba nepetella	w	0.32	0.89	Pieroni, 2000; Leporatti and Corradi, 2001; Camangi et al, 2003; Guarrera et al, 2005; Guarrera et al, 2005a; Scherrer et al, 2005; Passalacqua et al, 2007; Guarino et al, 2008; Cornara et al, 2009; Leonti et al, 2009; Savo et al, 2011; Leto et al, 2012; Di Sanzo et al, 2013; Tuttolomondo et al, 2014; Tuttolomondo et al, 2014a; Tuttolomondo et al, 2014b; Fortini et al, 2016; Menale et al, 2016; Motti and Motti, 2017; Ranfa and Bodesmo, 2017; Maruca et al, 2019; Lucchetti et al, 2019; Mattalia et al, 2020b; Motti et al, 2020	24	X	X	X	X	X	X	X	X	
Clinopodium vulgare L.	Lamiaceae	Clinopodio comune	w	0.01	0.22	Guarino et al, 2008	1	X			X					
Coffea arabica L.	Rubiaceae	Caffè	c	0.04	0.44	Palmese et al, 2001; Maxia et al, 2008; Savo et al, 2011	3			X	X	X	X			
Coffea sp.pl.	Rubiaceae	-	c	0.01	0.22	Danna et al, 2022	1				X		X			
Colchicum autumnale L.	Colchicaceae	Colchico	w	0.03	0.44	Lokar and Poldrini, 1988; De Natale and Pollio, 2007	2	X		X	X					X
Colchicum lusitanicum Brot.	Colchicaceae	Colchico	w	0.01	0.44	Leporatti et al, 1985b	1	X			X		X		X	
Colchicum neapolitanum (Ten.) Ten.	Colchicaceae	Colchico meridionale	w	0.01	0.44	Guarino et al, 2008	1	X			X				X	X
Colutea arborescens L.	Fabaceae	Vesicaria	w	0.03	0.33	Leporatti et al, 1985b; Guarino et al, 2008	2	X			X			X		
Conium maculatum L.	Apiaceae	Cicuta maggiore	w	0.16	0.78	Lokar and Poldrini, 1988; Ballero et al, 2001; Pieroni et al, 2004a; Pieroni and Quave, 2005; Guarino et al, 2008; Leto et al, 2012; Cornara et al, 2014; Menale and Muoio, 2014; Tuttolomondo et al, 2014; Tuttolomondo et al, 2014b; Bellia and Pieroni, 2015; Mattalia et al, 2021	12	X	X	X	X	X	X		X	
Consolida regalis Gray	Ranunculaceae	Consolida	w	0.04	0.33	Leporatti et al, 1985b; Leporatti and Ivancheva, 2003; Guarino et al, 2008	3	X		X	X					
Convallaria majalis L.	Asparagaceae	Mughetto	w	0.03	0.22	Lokar and Poldrini, 1988; Leporatti and Ivancheva, 2003	2						X	X		
Convolvulus althaeoides L.	Convolvulaceae	Vilucchio	w	0.01	0.11	Loi et al, 2005	1	X								
Convolvulus arvensis L.	Convolvulaceae	Vilucchio	w	0.11	0.44	Lokar and Poldrini, 1988; Leporatti and Corradi, 2001; Leporatti and Ivancheva, 2003; De Natale and Pollio, 2007; Leporatti and Ghedira, 2009; Leto et al, 2012; Di Sanzo et al, 2013; Lucchetti et al, 2019	8	X	X		X				X	
Convolvulus cantabrica L.	Convolvulaceae	Vilucchio a bicchiere	w	0.01	0.11	Leporatti and Corradi, 2001	1	X								

Coriandrum sativum L.	Apiaceae	Coriandolo	w/c	0.07	0.33	Leporatti and Ivancheva, 2003; Guarino et al, 2008; Leporatti and Ghedira, 2009; Bruschi et al, 2019; Galuzzo et al, 2021	5	X	X	X						
Cornus mas L.	Cornaceae	Corniolo	w	0.08	0.44	Leporatti et al, 1985b; Pieroni, 2000; Leporatti and Ivancheva, 2003; Di Sanzo et al, 2013; Fortini et al, 2016; Lucchetti et al, 2019	6	X	X	X				X		
Cornus sanguinea L.	Cornaceae	Sanguinello	w	0.03	0.22	Leporatti et al, 1985b; Di Novella et al, 2013	2	X	X							
Corydalis cava (L.) Schweigg. & Körte	Papaveraceae	Colombina	w	0.01	0.11	Guarino et al, 2008	1						X			
Corylus avellana L.	Betulaceae	Nocciolo	w/c	0.07	0.67	Leporatti et al, 1985b; Leporatti and Corradi, 2001; Leporatti and Ivancheva, 2003; Vitalini et al, 2015; Petalka et al, 2020	5	X	X	X		X	X	X		
Cota altissima (L.) J.Gay	Compositae	Camomilla orticante	w	0.03	0.11	Pieroni et al, 2004a; Pieroni and Quave, 2005;	2	X								
Cota tinctoria (L.) J.Gay	Compositae	Camomilla tintoria	w	0.01	0.11	Guarino et al, 2008	1			X						
Cotinus coggygria Scop.	Anacardiaceae	Sommacco	w	0.03	0.22	Lokar and Poldrini, 1988; Leporatti and Ivancheva, 2003	2	X			X					
Cotyledon macrantha A.Berger	Crassulaceae	-	w	0.01	0.22	Menale et al, 2016	1	X	X							
Cotyledon orbiculata L.	Crassulaceae		w	0.01	0.22	Palmese et al, 2001	1		X	X						
Crataegus laevigata (Poir.) DC.	Rosaceae	Biancospino	w	0.08	0.67	Leporatti and Ivancheva, 2003; Guarino et al, 2008; Vitalini et al, 2009; Guarrera et al, 2015; Vitalini et al, 2015; Mattalia et al, 2021	6	X	X	X	X		X	X		
Crataegus monogyna Jacq.	Rosaceae	Biancospino	w	0.49	0.89	Leporatti et al, 1985b; Lokar and Poldrini, 1988; Uncini Manganelli and Tomei, 1999; Leporatti and Corradi, 2001; Camangi et al, 2003; Leporatti and Ivancheva, 2003; Loi et al, 2004; Maccioni et al, 2004; Pieroni et al, 2004a; Pieroni et al, 2004b; Loi et al, 2005; Pieroni and Quave, 2005; Passalacqua et al, 2007; Cornara et al, 2009; Signorini et al, 2009; Idolo et al, 2010; Leto et al, 2012; Mattalia et al, 2012; Vitalini et al, 2012; Di Novella et al, 2013; Cornara et al, 2014; Menale and Muoio, 2014; Sansanelli and Tassoni, 2014; Tuttolomondo et al, 2014; Tuttolomondo et al, 2014b; Bellia and Pieroni, 2015; Guarrera et al, 2015; Vitalini et al, 2015; Bruschi et al, 2019; Lucchetti et al, 2019; Maruca et al, 2019; Mattalia et al, 2019; Mautone et al, 2019; Mattalia et al, 2020b; Fontefrancesco and Pieroni, 2020; Mattalia et al, 2020a; Petalka et al, 2020	37	X	X	X	X	X	X	X	X	
Crataegus rhipidophylla Gand.	Rosaceae	-	w	0.05	0.33	Bruni et al, 1997; Menale et al, 2006; Passalacqua et al, 2007; Menale and Muoio, 2014	4	X					X	X		
Crepis biennis Lapeyr.	Compositae	Radicchiella	w	0.01	0.11	Mattalia et al, 2020b	1		X							

Crepis capillaris (L.) Wallr.	Compositae	Radicchiella	w	0.03	0.22	Uncini Manganelli and Tomei, 1999; Pieroni, 2000	2	X						X		
Crepis conyzifolia (Gouan) A.Kern.	Compositae	Radicchiella	w	0.01	0.22	Petalka et al, 2020	1		X	X						
Crepis vesicaria L.	Compositae	Radicchiella vescicosa	w	0.05	0.56	Bruni et al, 1997; Loi et al, 2004; Sansanelli and Tassoni, 2014; Tuttolomondo et al, 2014; Geraci et al, 2018	5	X		X	X			X		X
Crithmum maritimum L.	Apiaceae	Finocchio di mare	w	0.03	0.44	Cornara et al, 2009; Savo et al, 2011	2	X		X	X	X				
Crocus imperati Ten.	Iridaceae	Zafferano selvatico	w	0.01	0.11	Guarino et al, 2008	1	X								
Crocus ligusticus Mariotti	Iridaceae	-	w	0.01	0.22	Cornara et al, 2014	1			X				X		
Crocus sativus L.	Iridaceae	Zafferano	c	0.03	0.33	Idolo et al, 2010; Bruschi et al, 2019	2	X			X		X			
Crocus vernus (L.) Hill	Iridaceae	Zafferano alpino	w	0.03	0.22	Pieroni, 2000; Vitalini et al, 2015	2	X	X							
Cruciata laevipes Opiz	Rubiaceae	Crocettona	w	0.01	0.11	Lucchetti et al, 2019	1	X								
Cucumis melo L.	Cucurbitaceae	Melone	w	0.01	0.11	Passalacqua et al, 2007	1	X								
Cucumis sativus L.	Cucurbitaceae	Cetriolo	c	0.09	0.56	Pieroni et al, 2004b; Guarrera and Lucia, 2007; Passalacqua et al, 2007; Guarino et al, 2008; Fortini et al, 2016; Menale et al, 2016; Motti and Motti, 2017	7	X	X	X	X	X				
Cucurbita maxima Duchesne	Cucurbitaceae	Zucca	c	0.09	0.33	Cornara et al, 2014; Dei Cas et al, 2015; Menale et al, 2016; Motti and Motti, 2017; Bruschi et al, 2019; Menale et al, 2021; Danna et al, 2022	7	X		X	X					
Cucurbita pepo L.	Cucurbitaceae	Zucchini	c	0.09	0.44	Leporatti et al, 1985b; Lokar and Poldrini, 1988; Passalacqua et al, 2007; Guarino et al, 2008; Leporatti and Ghedira, 2009; Menale et al, 2016; Mautone et al, 2019	7	X	X		X		X			
Cuminum cyminum L.	Apiaceae	Cumino	c	0.04	0.33	Leporatti and Ghedira, 2009; Tuttolomondo et al, 2014b; Galuzzo et al, 2021	3	X			X	X				
Cupressus macrocarpa Hartw.	Cupressaceae	Cipresso	c	0.01	0.22	Leporatti et al, 1985a	1	X	X							
Cupressus sempervirens L.	Cupressaceae	Cipresso	w/c	0.11	0.78	Bruni et al, 1997; Camangi et al, 2003; Gonzalez-Tejero et al, 2008; Guarino et al, 2008; Leporatti and Ghedira, 2009; Bruschi et al, 2019; Maruca et al, 2019; Mattalia et al, 2019	8	X	X		X	X		X	X	X
Curcuma longa L.	Zingiberaceae	Curcuma	c	0.01	0.33	Bruschi et al, 2019	1			X		X			X	
Cuscuta europaea L.	Convolvulaceae	Cuscuta	w	0.01	0.22	Leporatti and Ivancheva, 2003	1	X					X			
Cyanus segetum Hill	Compositae	-	w	0.08	0.56	Pieroni et al, 2004b; Bellia and Pieroni, 2015; Dei Cas et al, 2015; Menale et al, 2016; Petalka et al, 2020; Danna et al, 2022	6		X	X	X	X	X			
Cyclamen hederifolium Aiton	Primulaceae	Ciclamino	w	0.09	0.44	Leporatti et al, 1985b; Leporatti and Ivancheva, 2003; Pieroni et al, 2004a; Guarrera et al, 2005;	7	X	X	X	X					

						Pieroni and Quave, 2005; Guarino et al, 2008; Leto et al, 2012										
Cyclamen purpurascens Mill.	Primulaceae	Ciclamino alpino	w	0.03	0.56	Lokar and Poldrini, 1988; Petalka et al, 2020	2	X	X	X	X			X		
Cyclamen repandum Sm.	Primulaceae	Ciclamino primaverile	w	0.05	0.33	Leporatti and Ivancheva, 2003; Loi et al, 2004; Loi et al, 2005; Tuttolomondo et al, 2014a	4	X	X		X					
Cyclamen sp.pl.	Primulaceae	-	nd	0.01	0.11	Menale and Muoio, 2014	1	X								
Cydonia oblonga Mill.	Rosaceae	Cotogno	c	0.09	0.67	Bruni et al, 1997; Pieroni, 2000; Palmese et al, 2001; Leporatti and Ivancheva, 2003; Pieroni et al, 2004b; Loi et al, 2005; Guarino et al, 2008	7	X	X	X		X	X			X
Cymbalaria muralis P.Gaertn., B.Mey. & Scherb.	Plantaginaceae	Ciombolino	w	0.08	0.33	Lokar and Poldrini, 1988; De Natale and Pollio, 2007; Guarino et al, 2008; Idolo et al, 2010; Mautone et al, 2019; Mattalia et al, 2021	6		X	X					X	
Cynara cardunculus L.	Compositae	Carciofo	w/c	0.20	0.67	Pieroni, 2000; Ballero et al, 2001; Palmese et al, 2001; Loi et al, 2004; Pieroni et al, 2004a; Guarrera et al, 2005; Pieroni and Quave, 2005; Passalacqua et al, 2007; Leporatti and Ghedira, 2009; Idolo et al, 2010; Tuttolomondo et al, 2014; Dei Cas et al, 2015; Fortini et al, 2016; Bruschi et al, 2019; Mautone et al, 2019	15	X		X	X			X	X	X
Cynara scolymus L.	Compositae	Cardo domestico	c	0.14	0.56	Pieroni et al, 2004a; Pieroni and Quave, 2005; Scherrer et al, 2005; Guarino et al, 2008; Leporatti and Ghedira, 2009; Montesano et al, 2012; Motti and Motti, 2017; Mattalia et al, 2019; Galuzzo et al, 2021; Menale et al, 2021	10	X		X	X			X		X
Cynodon dactylon (L.) Pers	Poaceae	Gramigna	w	0.69	1.00	Leporatti et al, 1985a; Leporatti et al, 1985b; Lokar and Poldrini, 1988; Bruni et al, 1997; Uncini Manganelli and Tomei, 1999; Pieroni, 2000; Leporatti and Corradi, 2001; Camangi et al, 2003; Leporatti and Ivancheva, 2003; Maccioni et al, 2004; Loi et al, 2004; Pieroni et al, 2004a; Guarrera et al, 2005; Guarrera et al, 2005a; Scherrer et al, 2005; Menale et al, 2006; De Natale and Pollio, 2007; Passalacqua et al, 2007; Gonzalez-Tejero et al, 2008; Guarino et al, 2008; Cornara et al, 2009; Leonti et al, 2009; Leporatti and Ghedira, 2009; Motti et al, 2009; Pieroni and Giusti, 2009; Vitalini et al, 2009; Idolo et al, 2010; Savo et al, 2011; Leto et al, 2012; Montesano et al, 2012; Di Novella et al, 2013; Di Sanzo et al, 2013; Cornara et al, 2014; Menale and Muoio, 2014; Tuttolomondo et al, 2014; Tuttolomondo et al, 2014b; Bellia and Pieroni, 2015; Guarrera et al, 2015; Vitalini et al, 2015; Fortini et al, 2016; Menale et al, 2016; Motti and Motti, 2017; Gargano et al, 2018; Maruca et al, 2019; Mattalia et al, 2019; Mautone et al, 2019; Fontefrancesco and	51	X	X	X	X	X	X	X	X	X

						Pieroni, 2020; Mattalia et al, 2020a; Mattalia et al, 2020b; Menale et al, 2021; Danna et al, 2022									
Cynoglossum creticum Mill.	Boraginaceae	Lingua di cane	w	0.04	0.44	Palmese et al, 2001; Loi et al, 2005; Di Novella et al, 2013	3	X	X	X		X				
Cynoglossum magellense Ten.	Boraginaceae	Lingua di cane	w	0.01	0.11	Leporatti et al, 1985b	1		X							
Cynoglossum officinale L.	Boraginaceae	Lingua di cane	w	0.03	0.44	Guarino et al, 2008; Di Novella et al, 2013	2	X	X	X			X			
Cynoglossum sp.pl.	Boraginaceae	-	nd	0.01	0.11	Passalacqua et al, 2007	1		X							
Cyperus longus L.	Cyperaceae	Zigolo comune	w	0.01	0.11	Leporatti and Ghedira, 2009	1							X		
Cyperus rotundus L.	Cyperaceae	-	w	0.01	0.11	De Natale and Pollio, 2007	1			X						
Cytinus hypocistis (L.) L.	Cytinaceae	Ipocisto comune	w	0.01	0.22	Leporatti and Ghedira, 2009	1	X						X		
Cytisus scoparius (L.) Link	Fabaceae	Ginestra dei carbonai	w	0.07	0.56	Leporatti and Corradi, 2001; Menale et al, 2006; Passalacqua et al, 2007; Guarino et al, 2008; Vitalini et al, 2015	5	X	X		X			X	X	
Cytisus villosus Pourr.	Fabaceae	-	w	0.03	0.22	Leto et al, 2012; Tuttolomondo et al, 2014b	2		X						X	
Daphne gnidium L.	Thymelaeaceae	Dafne gnidio	w	0.04	0.56	Ballero et al, 2001; Leporatti and Ghedira, 2009; Leto et al, 2012	3	X	X	X				X	X	
Daphne laureola L.	Thymelaeaceae	Dafne lauro	W	0.01	0.11	Di Novella et al, 2013	1	X								
Daphne mezereum L.	Thymelaeaceae	Fior di stecco	w	0.03	0.22	Leporatti and Ivancheva, 2003; Danna et al, 2022	2		X		X					
Datura stramonium L.	Solanaceae	-	w	0.14	0.56	Bruni et al, 1997; Leporatti and Ivancheva, 2003; Gonzalez-Tejero et al, 2008; Guarino et al, 2008; Di Novella et al, 2013; Menale and Muoio, 2014; Bellia and Pieroni, 2015; Menale et al, 2016; Mautone et al, 2019; Menale et al, 2021	10	X	X			X	X	X		
Daucus carota L.	Apiaceae	Carota selvatica	w	0.31	1.00	Leporatti et al, 1985b; Lokar and Poldrini, 1988; Uncini Manganelli and Tomei, 1999; Pieroni, 2000; Palmese et al, 2001; Leporatti and Ivancheva, 2003; Loi et al, 2004; Pieroni et al, 2004b; Guarrera et al, 2005; Loi et al, 2005; Guarino et al, 2008; Cornara et al, 2009; Leporatti and Ghedira, 2009; Leto et al, 2012; Cornara et al, 2014; Tuttolomondo et al, 2014b; Dei Cas et al, 2015; Motti and Motti, 2017; Bruschi et al, 2019; Lucchetti et al, 2019; Mautone et al, 2019; Petalka et al, 2020; Galuzzo et al, 2021	23	X	X	X	X	X	X	X	X	X
Daucus carota L. subsp. *sativus* (Hoffm.) Arcang.	Apiaceae	Carota	c	0.03	0.44	Passalacqua et al, 2007; Menale et al, 2016	2	X	X	X		X				
Dianthus carthusianorum L.	Caryophyllaceae	Garofanino	w	0.01	0.44	Guarino et al, 2008	1			X	X	X	X			
Dianthus siculus C.Presl	Caryophyllaceae	-	w	0.01	0.11	Bruni et al, 1997	1		X							
Dictamnus albus L.	Rutaceae	Dittamo	w	0.03	0.33	Lokar and Poldrini, 1988; Leporatti and Ivancheva, 2003	2	X	X						X	

Digitalis ferruginea L.	Plantaginaceae	-	w	0.01	0.11	Guarino et al, 2008	1							X		
Digitalis grandiflora Mill.	Plantaginaceae	Digitale grande	w	0.01	0.11	Lokar and Poldrini, 1988	1							X		
Digitalis lutea subsp. *australis* (Ten.) Arcang.	Plantaginaceae	Digitale piccola	w	0.01	0.11	De Natale and Pollio, 2007	1							X		
Digitalis purpurea L.	Plantaginaceae	Digitale rossa	w	0.04	0.44	Bruni et al, 1997; Loi et al, 2004; Menale et al, 2006	3				X	X		X	X	
Dioscorea communis (L.) Caddick & Wilkin	Dioscoreaceae	Tamaro	w	0.23	0.78	Lokar and Poldrini, 1988; Bruni et al, 1997; Ballero et al, 2001; Palmese et al, 2001; Camangi et al, 2003; Leporatti and Ivancheva, 2003; Guarrera et al, 2005; Loi et al, 2005; Guarrera and Lucia, 2007; Guarino et al, 2008; Leporatti and Ghedira, 2009; Leto et al, 2012; Di Sanzo et al, 2013; Tuttolomondo et al, 2014; Tuttolomondo et al, 2014b; Guarrera et al, 2015; Mattalia et al, 2019	17	X	X	X	X	X		X	X	
Diplotaxis erucoides (L.) DC.	Brassicaceae	Rucola	w	0.03	0.33	Leporatti et al, 1985b; Lucchetti et al, 2019	2	X			X	X				
Diplotaxis tenuifolia (L.) DC.	Brassicaceae	Rucola	w/c	0.15	0.56	Leporatti and Corradi, 2001; Pieroni et al, 2004a; Pieroni and Quave, 2005; Gonzalez-Tejero et al, 2008; Savo et al, 2011; Montesano et al, 2012; Motti and Motti, 2017; Lucchetti et al, 2019; Mautone et al, 2019; Motti et al, 2020; Petalka et al, 2020	11	X	X	X	X				X	
Dipsacus fullonum L.	Caprifoliaceae	Scardaccione	w	0.08	0.78	Bruni et al, 1997; Guarino et al, 2008; Di Sanzo et al, 2013; Cornara et al, 2014; Motti and Motti, 2017; Petalka et al, 2020	6	X	X	X	X	X			X	X
Dittrichia graveolens (L.) Greuter	Compositae	Enula	w	0.03	0.44	Maxia et al, 2008; Galuzzo et al, 2021	2	X	X	X		X				
Dittrichia viscosa (L.) Greuter	Compositae	Enula appiccicosa	w	0.18	0.56	Uncini Manganelli and Tomei, 1999; Loi et al, 2004; Maccioni et al, 2004; Passalacqua et al, 2007; Guarino et al, 2008; Cornara et al, 2009; Leonti et al, 2009; Motti et al, 2009; Leto et al, 2012; Tuttolomondo et al, 2014; Tuttolomondo et al, 2014a; Tuttolomondo et al, 2014b; Gargano et al, 2018	13	X	X	X		X		X		
Drimia maritima (L.) Stearn	Asparagaceae	-	w	0.08	0.44	Bruni et al, 1997; Ballero et al, 2001; Guarrera et al, 2005; Leporatti and Ghedira, 2009; Leto et al, 2012; Tuttolomondo et al, 2014	6	X	X		X			X		
Drosera rotundifolia L.	Droseraceae	Acchiappamosche	w	0.03	0.33	Leporatti and Ivancheva, 2003; Petalka et al, 2020	2	X	X			X				
Dryas octopetala L.	Rosaceae	Camedrio alpino	w	0.03	0.33	Petalka et al, 2021	2			X			X	X		
Dryopteris filix-mas (L.) Schott.	Dryopteridaceae	Felce maschio	w	0.15	0.67	Lokar and Poldrini, 1988; Bruni et al, 1997; Leporatti and Ivancheva, 2003; Loi et al, 2004; Guarino et al, 2008; Leporatti and Ghedira, 2009; Di Sanzo et al, 2013; Dei Cas et al, 2015; Vitalini et al, 2015; Petalka et al, 2020; Danna et al, 2022	11	X	X	X		X			X	X

Dryopteris mindshelkensis Pavlov	Dryopteridaceae	-	w	0.01	0.11	Guarino et al, 2008	1			X						
Dysphania ambrosioides (L.) Mosyakin & Clemants	Amaranthaceae	Dripide	w	0.01	0.22	Leporatti and Ghedira, 2009	1	X			X					
Ecballium elaterium (L.) A.Rich.	Cucurbitaceae	Cocomero asinino	w	0.15	0.44	Leporatti and Corradi, 2001; Leporatti and Ivancheva, 2003; Pieroni et al, 2004a; Loi et al, 2005; Pieroni and Quave, 2005; Guarino et al, 2008; Leporatti and Ghedira, 2009; Leto et al, 2012; Montesano et al, 2012; Di Novella et al, 2013; Tuttolomondo et al, 2014	11	X	X		X		X			
Echinacea purpurea (L.) Moench	Compositae	Echinacea	w	0.01	0.11	Bruschi et al, 2019	1					X				
Echium italicum L.	Boraginaceae	Viperina maggiore	w	0.05	0.56	Pieroni, 2000; De Natale and Pollio, 2007; Leto et al, 2012; Tuttolomondo et al, 2014	4	X	X	X	X	X				
Echium plantagineum L.	Boraginaceae	-	w	0.01	0.11	Motti et al, 2009	1				X					
Echium vulgare L.	Boraginaceae	Viperina azzurra	w	0.03	0.44	Guarino et al, 2008; Ranfa and Bodesmo, 2017	2	X	X	X	X					
Elaeagnus rhamnoides (L.) A.Nelson	Elaeagnaceae	Olivagno	w	0.04	0.67	Vitalini et al, 2015; Petalka et al, 2020; Danna et al, 2022	3		X	X	X	X	X			X
Elymus hispidus (Opiz) Melderis	Poaceae	-	w	0.01	0.11	Tuttolomondo et al, 2014a	1				X					
Elymus repens (L.) Gould	Poaceae	-	w	0.11	0.78	Leporatti and Corradi, 2001; Leporatti and Ivancheva, 2003; Pieroni and Quave, 2005; Maxia et al, 2008; Tuttolomondo et al, 2014a; Menale et al, 2016; Lucchetti et al, 2019; Petalka et al, 2020	8	X	X	X	X	X	X	X		
Ephedra aphylla Forssk.	Ephedraceae	Efedra	w	0.01	0.11	Gonzalez-Tejero et al, 2008	1			X						
Epilobium angustifolium L.	Onagraceae	Garofanino a foglie strette	w	0.07	0.33	Menale et al, 2006; Fontefrancesco and Pieroni, 2020; Petalka et al, 2020; Mattalia et al, 2021; Danna et al, 2022	5	X			X					X
Epilobium montanum L.	Onagraceae	Garofanino di montagna	w	0.03	0.22	Fortini et al, 2016; Petalka et al, 2020	2				X					X
Epilobium palustre L.	Onagraceae	Garofanino acquatico	w	0.01	0.11	Petalka et al, 2020	1				X					
Epilobium parviflorum Schreb.	Onagraceae	-	w	0.03	0.22	Fortini et al, 2016; Petalka et al, 2020	2				X					X
Epilobium sp.pl.	Onagraceae	-	nd	0.01	0.11	Bottoni et al, 2020	1			X						
Equisetum arvense L.	Equisetaceae	Equiseto dei campi	w	0.38	1.00	Lokar and Poldrini, 1988; Uncini Manganelli and Tomei, 1999; Pieroni, 2000; Loi et al, 2004; Maccioni et al, 2004; Pieroni et al, 2004b; Gonzalez-Tejero et al, 2008; Guarino et al, 2008; Cornara et al, 2009; Leto et al, 2012; Mattalia et al, 2012; Vitalini et al, 2012; Tuttolomondo et al, 2014; Tuttolomondo et al, 2014b; Bellia and Pieroni, 2015; Dei Cas et al,	28	X	X	X	X	X	X	X	X	X

						2015; Vitalini et al, 2015; Fortini et al, 2016; Gargano et al, 2018; Bruschi et al, 2019; Lucchetti et al, 2019; Maruca et al, 2019; Mattalia et al, 2019; Mautone et al, 2019; Fontefrancesco and Pieroni, 2020; Mattalia et al, 2020a; Petalka et al, 2020; Danna et al, 2022										
Equisetum giganteum L.	Equisetaceae	-	w	0.01	0.11	Leto et al, 2012	1				X					
Equisetum palustre L.	Equisetaceae	Equiseto acquatico	w	0.01	0.11	Petalka et al, 2020	1		X							
Equisetum pratense Ehrh.	Equisetaceae	Equiseto	w	0.01	0.11	Petalka et al, 2020	1		X							
Equisetum ramosissimum Desf.	Equisetaceae	-	w	0.01	0.11	Cornara et al, 2009	1				X					
Equisetum sp.pl.	Equisetaceae	-	nd	0.04	0.67	Idolo et al, 2010; Savo et al, 2011; Cornara et al, 2014	3	X	X		X	X		X	X	
Equisetum telmateia Ehrh.	Equisetaceae	Equiseto gigante	w	0.14	0.89	Leporatti et al, 1985b; Uncini Manganelli and Tomei, 1999; Leporatti and Corradi, 2001; Camangi et al, 2003; Leporatti and Ivancheva, 2003; Guarino et al, 2008; Cornara et al, 2009; Leporatti and Ghedira, 2009; Fortini et al, 2016; Lucchetti et al, 2019	10	X	X	X	X	X		X	X	X
Erica arborea L.	Ericaceae	Erica arborea	w	0.03	0.22	Leporatti and Ghedira, 2009; Fontefrancesco and Pieroni, 2020	2				X				X	
Erica herbacea L.	Ericaceae	-	w	0.05	0.44	Lokar and Poldrini, 1988; Bellia and Pieroni, 2015; Vitalini et al, 2015; Petalka et al, 2020	4	X			X	X	X			
Erica sp.pl.	Ericaceae	-	nd	0.01	0.11	Ballero et al, 2001	1				X					
Erigeron acris L.	Compositae	Cespica acre	w	0.03	0.33	Pieroni et al, 2004a; Pieroni and Quave, 2005	2	X	X						X	
Erigeron alpinus L.	Compositae	Cespica alpina	w	0.01	0.33	Petalka et al, 2020	1		X	X	X					
Erigeron canadensis L.	Compositae	Cespica canadese	w	0.04	0.67	De Natale and Pollio, 2007; Guarino et al, 2008; Leporatti and Ghedira, 2009	3	X		X	X	X			X	X
Eriobotrya japonica (Thunb.) Lindl.	Rosaceae	Nespolo del giappone	c	0.05	0.33	Uncini Manganelli and Tomei, 1999; Scherrer et al, 2005; Motti et al, 2009; Menale et al, 2016	4	X				X			X	
Eriophorum angustifolium Honck.	Cyperaceae	Erioforo	w	0.01	0.22	Petalka et al, 2020	1		X	X						
Eriophorum scheuchzeri Hoppe	Cyperaceae	Erioforo	w	0.03	0.33	Dei Cas et al, 2015; Vitalini et al, 2015	2		X			X		X		
Erodium cicutarium (L.) L'Hér.	Geraniaceae	Becco di gru	w	0.04	0.44	Leporatti and Ivancheva, 2003; Loi et al, 2004; Leporatti and Ghedira, 2009	3	X	X		X			X		
Eruca vesicaria (L.) Cav.	Brassicaceae	Rucola comune	w/c	0.03	0.44	Guarino et al, 2008; Motti et al, 2020	2	X	X	X						X
Eryngium amethystinum L.	Apiaceae	Calcatreppola ametista	w	0.04	0.33	De Natale and Pollio, 2007; Guarino et al, 2008; Petalka et al, 2020	3	X			X	X				

Eryngium campestre L.	Apiaceae	Calcatreppola	w	0.07	0.56	Leporatti and Ivancheva, 2003; Guarino et al, 2008; Leto et al, 2012; Tuttolomondo et al, 2014; Petalka et al, 2020	5	X	X		X	X	X			
Eryngium maritimum L.	Apiaceae	Calcatreppola marina	w	0.01	0.11	Palmese et al, 2001	1	X								
Erysimum cheiri (L.) Crantz	Brassicaceae	-	w	0.03	0.22	Passalacqua et al, 2007; Guarino et al, 2008	2				X			X		
Erysimum sp.pl.	Brassicaceae	-	nd	0.01	0.11	Di Sanzo et al, 2013	1	X								
Eucalyptus camaldulensis Dehnh.	Myrtaceae	Eucalipto	w/c	0.08	0.44	Scherrer et al, 2005; Gonzalez-Tejero et al, 2008; Guarino et al, 2008; Cornara et al, 2009; Motti and Motti, 2017; Lucchetti et al, 2019	6	X		X		X				X
Eucalyptus globulus Labill.	Myrtaceae	Eucalipto	w/c	0.22	0.89	Uncini Manganelli and Tomei, 1999; Leporatti and Ivancheva, 2003; Maccioni et al, 2004; Loi et al, 2005; Guarino et al, 2008; Maxia et al, 2008; Cornara et al, 2009; Savo et al, 2011; Montesano et al, 2012; Cornara et al, 2014; Menale and Muoio, 2014; Menale et al, 2016; Motti and Motti, 2017; Bruschi et al, 2019; Mautone et al, 2019; Menale et al, 2021	16	X	X	X	X	X	X	X	X	
Eucalyptus sp.pl.	Myrtaceae	-	nd	0.08	0.67	Ballero et al, 2001; Palmese et al, 2001; Leporatti and Ghedira, 2009; Motti et al, 2009; Mattalia et al, 2019; Mattalia et al, 2020b	6	X	X	X		X		X		X
Euonymus europaeus L.	Celastraceae	Evonimo	w	0.05	0.22	Leporatti et al, 1985b; Bruni et al, 1997; Guarino et al, 2008; Di Novella et al, 2013	4	X	X							
Eupatorium cannabinum L.	Compositae	Canapa acquatica	w	0.07	0.67	Leporatti et al, 1985b; Lokar and Poldrini, 1988; Pieroni et al, 2004b; Guarino et al, 2008; Galuzzo et al, 2021	5	X	X	X	X	X			X	
Euphorbia amygdaloides L.	Euphorbiaceae	-	w	0.04	0.22	De Natale and Pollio, 2007; Guarino et al, 2008; Menale et al, 2016	3		X				X			
Euphorbia characias L.	Euphorbiaceae	Euforbia cespugliosa	w	0.04	0.22	Di Novella et al, 2013; Tuttolomondo et al, 2014; Guarrera et al, 2015	3	X	X							
Euphorbia characias subsp. *wulfenii* (Hoppe ex W.D.J.Koch) Radcl.-Sm.	Euphorbiaceae	Euforbia cespugliosa	w	0.01	0.22	Lokar and Poldrini, 1988	1	X	X							
Euphorbia cyparissias L.	Euphorbiaceae	Euforbia cipressina	w	0.08	0.22	Pieroni, 2000; Pieroni et al, 2004a; Pieroni and Quave, 2005; Dei Cas et al, 2015; Vitalini et al, 2015; Fortini et al, 2016	6	X	X							
Euphorbia dendroides L.	Euphorbiaceae	Euforbia arborea	w	0.07	0.11	Savo et al, 2011; Di Sanzo et al, 2013; Gargano et al, 2018; Mautone et al, 2019; Menale et al, 2021	5		X							
Euphorbia falcata L.	Euphorbiaceae	-	w	0.01	0.22	Leporatti et al, 1985b	1	X	X							
Euphorbia helioscopia L.	Euphorbiaceae	Euforbia calenzuola	w	0.20	0.56	Pieroni et al, 2004a; Pieroni and Quave, 2005; De Natale and Pollio, 2007; Guarino et al, 2008; Cornara et al, 2009; Signorini et al, 2009; Idolo et al, 2010; Di Novella et al, 2013; Tuttolomondo et al,	15	X	X		X		X		X	

						2014; Vitalini et al, 2015; Fortini et al, 2016; Menale et al, 2016; Motti and Motti, 2017; Mattalia et al, 2021; Danna et al, 2022										
Euphorbia lathyris L.	Euphorbiaceae	Euforbia catapunza	w	0.05	0.22	Pieroni, 2000; Guarino et al, 2008; Cornara et al, 2009; Tuttolomondo et al, 2014b	4	X	X							
Euphorbia paralias L.	Euphorbiaceae	-	w	0.01	0.11	Guarrera and Lucia, 2007	1						X			
Euphorbia peplus L.	Euphorbiaceae	Euforbia minore	w	0.03	0.33	Guarino et al, 2008; Lucchetti et al, 2019	2	X	X				X			
Euphorbia rigida M.Bieb.	Euphorbiaceae	-	w	0.03	0.11	Leto et al, 2012; Gargano et al, 2018	2								X	
Euphorbia seguieriana Neck.	Euphorbiaceae	-	w	0.01	0.11	Danna et al, 2022	1		X							
Euphorbia sp.pl.	Euphorbiaceae	-	nd	0.03	0.11	Pieroni et al, 2004b; Loi et al, 2005	2		X							
Euphrasia minima Jacq. ex DC.	Orobanchaceae	Eufrasia	w	0.01	0.22	Petalka et al, 2020	1					X			X	
Euphrasia officinalis L.	Orobanchaceae	Eufrasia	w	0.05	0.44	Leporatti and Ivancheva, 2003; Vitalini et al, 2009; Petalka et al, 2020; Danna et al, 2022	4		X	X		X			X	
Euphrasia officinalis subsp. *versicolor* (A.Kern.) Vitek	Orobanchaceae	Eufrasia	w	0.01	0.22	Petalka et al, 2020	1					X			X	
Euphrasia rostkoviana Hayne	Orobanchaceae	-	w	0.04	0.67	Vitalini et al, 2012; Vitalini et al, 2015; Petalka et al, 2020	3	X	X			X	X	X	X	
Euphrasia sp.pl.	Orobanchaceae	-	nd	0.01	0.22	Lokar and Poldrini, 1988	1		X	X						
Euphrasia stricta D. Wolff	Orobanchaceae	-	w	0.01	0.22	Petalka et al, 2020	1					X			X	
Fagopyrum esculentum Moench	Polygonaceae	-	w	0.01	0.11	Vitalini et al, 2015	1							X		
Fagus sylvatica L.	Fagaceae	Faggio	w	0.08	0.56	Leporatti et al, 1985b; Camangi et al, 2003; Vitalini et al, 2009; Di Novella et al, 2013; Petalka et al, 2020; Mattalia et al, 2021	6	X	X	X		X	X			
Ferula communis L.	Apiaceae	Ferula	w	0.04	0.67	Loi et al, 2005; Guarino et al, 2008; Tuttolomondo et al, 2014b	3		X	X			X	X	X	X
Ficaria verna Huds.	Ranunculaceae	Favagello	w	0.11	0.56	Maccioni et al, 2004; Guarrera et al, 2005; Guarrera and Lucia, 2007; Passalacqua et al, 2007; Guarino et al, 2008; Di Sanzo et al, 2013; Ranfa and Bodesmo, 2017; Lucchetti et al, 2019	8	X	X	X				X	X	
Ficaria verna subsp. *ficariiformis* (Rouy & Foucaud) B.Walln.	Ranunculaceae	Favagello	w	0.01	0.11	Menale et al, 2016	1	X								
Ficus carica L.	Moraceae	Fico	w/c	0.59	0.78	Leporatti et al, 1985a; Lokar and Poldrini, 1988; Bruni et al, 1997; Uncini Manganelli and Tomei, 1999; Pieroni, 2000; Ballero et al, 2001; Leporatti and Corradi, 2001; Palmese et al, 2001; Camangi et al, 2003; Leporatti and Ivancheva, 2003; Maccioni et al, 2004; Pieroni et al, 2004a; Guarrera et al, 2005; Guarrera et al, 2005a; Loi et al, 2005; Pieroni and	44	X	X	X	X	X		X	X	

						Quave, 2005; Scherrer et al, 2005; Menale et al, 2006; De Natale and Pollio, 2007; Passalacqua et al, 2007; Gonzalez-Tejero et al, 2008; Guarino et al, 2008; Maxia et al, 2008; Cornara et al, 2009; Leporatti and Ghedira, 2009; Motti et al, 2009; Idolo et al, 2010; Savo et al, 2011; Montesano et al, 2012; Di Novella et al, 2013; Cornara et al, 2014; Menale and Muoio, 2014; Guarrera et al, 2015; Vitalini et al, 2015; Fortini et al, 2016; Menale et al, 2016; Motti and Motti, 2017; Lucchetti et al, 2019; Maruca et al, 2019; Mautone et al, 2019; Mattalia et al, 2020a; Mattalia et al, 2020b; Mattalia et al, 2021; Menale et al, 2021										
Filipendula ulmaria (L.) Maxim.	Rosaceae	Olmaria comune	w	0.05	0.78	Leporatti and Ivancheva, 2003; Dei Cas et al, 2015; Petalka et al, 2020; Danna et al, 2022	4		X	X	X	X	X		X	X
Filipendula vulgaris Moench	Rosaceae	Olmaria peperina	w	0.01	0.33	Guarino et al, 2008	1	X		X	X					
Foeniculum vulgare Mill.	Apiaceae	Finocchio	w/c	0.66	0.89	Leporatti et al, 1985b; Lokar and Poldrini, 1988; Uncini Manganelli and Tomei, 1999; Pieroni, 2000; Ballero et al, 2001; Leporatti and Corradi, 2001; Palmese et al, 2001; Camangi et al, 2003; Leporatti and Ivancheva, 2003; Maccioni et al, 2004; Guarrera et al, 2005; Guarrera et al, 2005a; Loi et al, 2005; Scherrer et al, 2005; Passalacqua et al, 2007; Gonzalez-Tejero et al, 2008; Guarino et al, 2008; Maxia et al, 2008; Cornara et al, 2009; Leonti et al, 2009; Leporatti and Ghedira, 2009; Motti et al, 2009; Idolo et al, 2010; Savo et al, 2011; Leto et al, 2012; Menale and Muoio, 2014; Tuttolomondo et al, 2014; Tuttolomondo et al, 2014a; Tuttolomondo et al, 2014b; Guarrera et al, 2015; Fortini et al, 2016; Menale et al, 2016; Motti and Motti, 2017; Ranfa and Bodesmo, 2017; Sansanelli et al, 2017; Gargano et al, 2018; Geraci et al, 2018; Bruschi et al, 2019; Lucchetti et al, 2019; Maruca et al, 2019; Mattalia et al, 2019; Mautone et al, 2019; Mattalia et al, 2020a; Mattalia et al, 2020b; Motti et al, 2020; Galuzzo et al, 2021; Mattalia et al, 2021; Menale et al, 2021; Danna et al, 2022	49	X	X	X	X	X	X	X	X	
Fragaria moschata (Duchesne) Duchesne	Rosaceae	Fragolina	w	0.03	0.56	Lokar and Poldrini, 1988; Leporatti and Ivancheva, 2003	2	X	X	X	X		X			
Fragaria vesca L.	Rosaceae	Fragolina	w/c	0.20	1.00	Leporatti et al, 1985b; Lokar and Poldrini, 1988; Leporatti and Ivancheva, 2003; Pieroni et al, 2004b; Guarrera et al, 2005; Passalacqua et al, 2007; Guarino et al, 2008; Savo et al, 2011; Vitalini et al, 2012; Tuttolomondo et al, 2014a; Bellia and Pieroni,	15	X	X	X	X	X	X	X	X	X

						2015; Motti and Motti, 2017; Fontefrancesco and Pieroni, 2020; Petalka et al, 2020; Danna et al, 2022										
Fragaria viridis Weston	Rosaceae	-	w	0.04	0.67	Leporatti and Ivancheva, 2003; Mattalia et al, 2020b; Petalka et al, 2020	3	X	X	X		X	X			X
Frangula alnus Mill.	Rhamnaceae	Frangola comune	w	0.05	0.33	Lokar and Poldrini, 1988; Leporatti and Ivancheva, 2003; Menale et al, 2006; Petalka et al, 2020	4	X				X				X
Fraxinus excelsior L.	Oleaceae	Frassino maggiore	w	0.11	0.89	Leporatti et al, 1985b; Pieroni et al, 2004a; Pieroni and Quave, 2005; Mattalia et al, 2012; Bellia and Pieroni, 2015; Dei Cas et al, 2015; Vitalini et al, 2015; Petalka et al, 2020	8	X	X	X	X	X		X	X	X
Fraxinus ornus L.	Oleaceae	Orniello	w	0.19	0.89	Lokar and Poldrini, 1988; Bruni et al, 1997; Pieroni, 2000; Camangi et al, 2003; Maccioni et al, 2004; Guarrera and Lucia, 2007; Guarino et al, 2008; Cornara et al, 2009; Savo et al, 2011; Di Sanzo et al, 2013; Cornara et al, 2014; Guarrera et al, 2015; Mautone et al, 2019; Petalka et al, 2020	14	X	X	X	X	X		X	X	X
Fraxinus sp.pl.	Oleaceae	-	nd	0.03	0.22	Bruschi et al, 2019; Mattalia et al, 2019	2		X		X					
Fumaria capreolata L.	Papaveraceae	Fumaria bianca	w	0.04	0.67	Guarino et al, 2008; Leporatti and Ghedira, 2009; Cornara et al, 2014	3	X	X	X	X			X	X	
Fumaria officinalis L.	Papaveraceae	Fumaria comune	w	0.18	0.89	Leporatti et al, 1985b; Lokar and Poldrini, 1988; Uncini Manganelli and Tomei, 1999; Leporatti and Ivancheva, 2003; Loi et al, 2004; Menale et al, 2006; De Natale and Pollio, 2007; Guarino et al, 2008; Leporatti and Ghedira, 2009; Vitalini et al, 2015; Lucchetti et al, 2019; Fontefrancesco and Pieroni, 2020; Petalka et al, 2020	13	X	X	X	X	X	X	X	X	
Galactites tomentosa Moench	Compositae	Scarlina	w	0.01	0.11	Motti et al, 2009	1		X							
Galanthus nivalis L.	Amaryllidaceae	Bucaneve	w	0.01	0.33	Leporatti and Ivancheva, 2003	1	X	X						X	
Galega officinalis L.	Fabaceae	-	w	0.07	0.67	Lokar and Poldrini, 1988; Leporatti and Ivancheva, 2003; Guarino et al, 2008; Vitalini et al, 2015; Petalka et al, 2020	5	X		X	X	X			X	X
Galeopsis ladanum subsp. *angustifolia* (Ehrh. ex Hoffm.) Gaudin	Lamiaceae	Canapetta viola	w	0.01	0.22	Guarino et al, 2008	1				X	X				
Galeopsis pubescens Besser	Lamiaceae	Canapetta pelosa	w	0.01	0.44	Petalka et al, 2020	1		X	X		X	X			
Galeopsis tetrahit L.	Lamiaceae	Canapetta	w	0.03	0.22	Leporatti and Ivancheva, 2003; Guarino et al, 2008	2				X	X				
Galium album Mill.	Rubiaceae	Caglio bianco	w	0.03	0.11	Pieroni et al, 2004a; Pieroni and Quave, 2005	2		X							
Galium aparine L.	Rubiaceae	Attaccabraghe	w	0.07	0.67	Leporatti and Ivancheva, 2003; Guarino et al, 2008; Dei Cas et al, 2015; Lucchetti et al, 2019; Petalka et al, 2020	5	X	X	X	X	X	X			
Galium lucidum All.	Rubiaceae	Caglio lucido	w	0.01	0.11	Savo et al, 2011	1								X	
Galium mollugo L.	Rubiaceae	Caglio tirolese	w	0.03	0.56	Lokar and Poldrini, 1988; Petalka et al, 2020	2	X	X		X		X		X	

Galium odoratum (L.) Scop.	Rubiaceae	Caglio profumato	w	0.05	0.67	Leporatti and Ivancheva, 2003; Guarino et al, 2008; Vitalini et al, 2009; Petalka et al, 2020	4	X	X	X	X		X	X		
Galium verum L.	Rubiaceae	Caglio giallo	w	0.08	0.67	Leporatti and Ivancheva, 2003; Pieroni et al, 2004a; Pieroni and Quave, 2005; Guarino et al, 2008; Mautone et al, 2019; Petalka et al, 2020	6	X	X	X	X	X	X			
Genista anglica L.	Fabaceae	-	w	0.01	0.22	Guarrera and Lucia, 2007	1		X		X					
Genista tinctoria L.	Fabaceae	Ginestra minore	w	0.04	0.22	Leporatti and Ivancheva, 2003; Guarino et al, 2008; Petalka et al, 2020	3	X			X					
Gentiana acaulis L.	Gentianaceae	Genziana	w	0.14	0.89	Pieroni, 2000; Pieroni and Giusti, 2009; Vitalini et al, 2009; Mattalia et al, 2012; Bellia and Pieroni, 2015; Dei Cas et al, 2015; Vitalini et al, 2015; Fontefrancesco and Pieroni, 2020; Petalka et al, 2020; Danna et al, 2022	10	X	X	X	X	X	X	X	X	
Gentiana dinarica Beck	Gentianaceae	Genziana appenninica	w	0.03	0.44	Idolo et al, 2010; Fortini et al, 2016	2	X		X			X	X		
Gentiana ligustica R.Vilm. & Chopinet	Gentianaceae	Genziana ligure	w	0.01	0.44	Cornara et al, 2014	1	X		X				X		X
Gentiana lutea L.	Gentianaceae	Genziana maggiore	w	0.32	1.00	Leporatti et al, 1985b; Lokar and Poldrini, 1988; Bruni et al, 1997; Leporatti and Ivancheva, 2003; Loi et al, 2004; Loi et al, 2005; Guarino et al, 2008; Pieroni and Giusti, 2009; Vitalini et al, 2009; Idolo et al, 2010; Mattalia et al, 2012; Di Novella et al, 2013; Di Sanzo et al, 2013; Cornara et al, 2014; Bellia and Pieroni, 2015; Vitalini et al, 2015; Fortini et al, 2016; Bruschi et al, 2019; Bottoni et al, 2020; Fontefrancesco and Pieroni, 2020; Mattalia et al, 2020a; Petalka et al, 2020; Mattalia et al, 2021; Danna et al, 2022	24	X	X	X	X	X	X	X	X	X
Gentiana punctata L.	Gentianaceae	Genziana punteggiata	w	0.07	0.67	Vitalini et al, 2012; Dei Cas et al, 2015; Vitalini et al, 2015; Petalka et al, 2020; Danna et al, 2022	5	X		X	X	X		X		X
Gentiana purpurea L.	Gentianaceae	Genziana rossa	w	0.01	0.44	Vitalini et al, 2012	1	X		X	X			X		
Gentiana verna L.	Gentianaceae	Genziana primaticcia	w	0.05	0.56	Mattalia et al, 2012; Fortini et al, 2016; Fontefrancesco and Pieroni, 2020; Danna et al, 2022	4	X		X	X	X	X			
Geranium dissectum L.	Gentianaceae	Geranio selvatico	w	0.01	0.11	Lucchetti et al, 2019	1	X								
Geranium pratense L.	Gentianaceae	-	w	0.01	0.56	Petalka et al, 2020	1		X	X		X	X	X		
Geranium robertianum L.	Gentianaceae	Geranio di San Roberto	w	0.11	0.89	Lokar and Poldrini, 1988; Bruni et al, 1997; Loi et al, 2004; Leporatti and Ghedira, 2009; Vitalini et al, 2015; Fontefrancesco and Pieroni, 2020; Petalka et al, 2020; Danna et al, 2022	8	X	X	X	X	X	X	X		X
Geranium sanguineum L.	Gentianaceae	-	w	0.03	0.44	Leporatti and Ivancheva, 2003; Petalka et al, 2020	2	X				X	X	X		
Geum montanum L.	Rosaceae	Cariofillata montana	w	0.01	0.33	Petalka et al, 2020	1				X	X		X		
Geum reptans L.	Rosaceae	Cariofillata montana	w	0.01	0.33	Petalka et al, 2020	1					X	X	X		

Geum rivale L.	Rosaceae	Cariofillata fluviale	w	0.01	0.33	Petalka et al, 2020	1					X	X	X		
Geum urbanum L.	Rosaceae	Cariofillata comune	w	0.08	0.67	Lokar and Poldrini, 1988; Leporatti and Ivancheva, 2003; Guarino et al, 2008; Di Sanzo et al, 2013; Tuttolomondo et al, 2014a; Petalka et al, 2020	6	X	X	X		X	X	X		
Glaucium flavum Crantz	Papaveraceae	-	w	0.01	0.11	Leporatti and Ivancheva, 2003	1		X							
Glebionis coronaria (L.) Cass. ex Spach	Compositae	Crisantemo giallo	w	0.01	0.11	Tuttolomondo et al, 2014a	1						X			
Glechoma hederacea L.	Lamiaceae	Ellera comune	w	0.05	1.00	Lokar and Poldrini, 1988; Leporatti and Ivancheva, 2003; Petalka et al, 2020; Galuzzo et al, 2021	4	X	X	X	X	X	X	X	X	X
Globularia alypum L.	Plantaginaceae	Vedovella cespitosa	w	0.03	0.22	Leporatti and Ghedira, 2009; Tuttolomondo et al, 2014a	2	X			X					
Globularia cordifolia L.	Plantaginaceae	Vedovella celeste	w	0.01	0.22	Petalka et al, 2020	1				X	X				
Glycyrrhiza glabra L.	Fabaceae	Liquirizia	w	0.20	0.78	Bruni et al, 1997; Leporatti and Corradi, 2001; Leporatti and Ivancheva, 2003; Pieroni et al, 2004a; Pieroni and Quave, 2005; Menale et al, 2006; Passalacqua et al, 2007; Maxia et al, 2008; Leporatti and Ghedira, 2009; Leto et al, 2012; Montesano et al, 2012; Tuttolomondo et al, 2014; Tuttolomondo et al, 2014b; Sansanelli et al, 2017; Bruschi et al, 2019	15	X	X	X	X	X		X	X	
Gratiola officinalis L.	Plantaginaceae	Graziella	w	0.03	0.44	Lokar and Poldrini, 1988; Leporatti and Ivancheva, 2003	2	X			X			X		X
Gymnadenia nigra (L.) Rchb.f.	Orchidaceae	Manina scura	w	0.03	0.33	Vitalini et al, 2012; Danna et al, 2022	2	X			X	X				
Harpagophytum procumbens (Burch.) DC. ex Meisn.	Pedaliaceae	-	w	0.01	0.22	Bruschi et al, 2019	1			X					X	
Hedera helix L.	Araliaceae	Edera	w	0.47	0.89	Leporatti et al, 1985a; Leporatti et al, 1985b; Lokar and Poldrini, 1988; Bruni et al, 1997; Ballero et al, 2001; Leporatti and Corradi, 2001; Camangi et al, 2003; Leporatti and Ivancheva, 2003; Loi et al, 2004; Pieroni et al, 2004b; Guarrera et al, 2005; Guarrera et al, 2005a; Loi et al, 2005; De Natale and Pollio, 2007; Passalacqua et al, 2007; Gonzalez-Tejero et al, 2008; Guarino et al, 2008; Maxia et al, 2008; Leporatti and Ghedira, 2009; Signorini et al, 2009; Vitalini et al, 2009; Idolo et al, 2010; Leto et al, 2012; Cornara et al, 2014; Tuttolomondo et al, 2014; Guarrera et al, 2015; Vitalini et al, 2015; Fortini et al, 2016; Bruschi et al, 2019; Lucchetti et al, 2019; Maruca et al, 2019; Mautone et al, 2019; Fontefrancesco and Pieroni, 2020; Petalka et al, 2020; Danna et al, 2022	35	X	X	X	X	X	X	X	X	

Hedera sp.pl.	Araliaceae	-	nd	0.01	0.11	Mattalia et al, 2020a	1			X						
Hedysarum coronarium L.	Fabaceae	Sulla	w	0.01	0.11	Lucchetti et al, 2019	1				X					
Helianthemum oelandicum subsp. *alpestris* (Jacq.) Breistr.	Cistaceae	Eliantemo	w	0.01	0.33	Petalka et al, 2020	1		X	X				X		
Helianthus annuus L.	Compositae	Eliantemo annuale	c	0.05	0.78	Leporatti and Corradi, 2001; Leporatti and Ivancheva, 2003; Guarino et al, 2008; Leporatti and Ghedira, 2009	4	X	X	X	X		X	X		X
Helianthus tuberosus L.	Compositae	Girasole del canada	w	0.08	0.33	Leporatti and Ivancheva, 2003; Guarrera and Lucia, 2007; Guarino et al, 2008; Di Sanzo et al, 2013; Motti et al, 2020; Galuzzo et al, 2021	6	X			X					X
Helichrysum italicum (Roth) G.Don	Compositae	Elicriso	w	0.18	0.67	Leporatti et al, 1985b; Bruni et al, 1997; Uncini Manganelli and Tomei, 1999; Pieroni, 2000; Camangi et al, 2003; Passalacqua et al, 2007; Guarino et al, 2008; Cornara et al, 2009; Di Novella et al, 2013; Fortini et al, 2016; Mattalia et al, 2019; Mattalia et al, 2020a; Galuzzo et al, 2021	13	X	X	X		X	X		X	
Helichrysum italicum subsp. *microphyllum* (Willd.) Nyman	Compositae	Elicriso	w	0.03	0.22	Ballero et al, 2001; Loi et al, 2005	2		X			X				
Heliotropium europaeum L.	Boraginaceae	Eliotropio selvatico	w	0.04	0.33	Passalacqua et al, 2007; Leporatti and Ghedira, 2009; Maruca et al, 2019	3	X	X		X					
Helleborus bocconei Ten.	Ranunculaceae	-	w	0.04	0.33	Leporatti and Corradi, 2001; Guarino et al, 2008; Leto et al, 2012	3	X	X				X			
Helleborus foetidus L.	Ranunculaceae	Elleboro puzzolente	w	0.09	0.56	Pieroni, 2000; Leporatti and Corradi, 2001; Scherrer et al, 2005; Guarino et al, 2008; Idolo et al, 2010; Cornara et al, 2014; Mattalia et al, 2021	7	X	X	X	X			X		
Helleborus odorus subsp. *laxus* (Host) Merxm. & Podlech	Ranunculaceae	Elleboro	w	0.01	0.22	Lokar and Poldrini, 1988	1			X				X		
Helleborus odorus Waldst. & Kit. ex Willd.	Ranunculaceae	Elleboro	w	0.01	0.22	Leporatti and Ivancheva, 2003	1						X	X		
Helleborus sp.pl.	Ranunculaceae	-	nd	0.01	0.22	Camangi et al, 2003	1	X	X							
Helleborus viridis L.	Ranunculaceae	Elleboro selvatico	w	0.01	0.22	Pieroni, 2000	1	X	X							
Helminthotheca echioides (L.) Holub	Compositae	Aspraggine	w	0.01	0.11	Tuttolomondo et al, 2014	1		X							
Hepatica nobilis Mill.	Ranunculaceae	Erba trinità	w	0.03	0.56	Leporatti et al, 1985b; Guarino et al, 2008	2	X		X	X		X	X		
Heracleum sphondylium L.	Apiaceae	Panace comune	w	0.05	0.33	Leporatti and Ivancheva, 2003; Dei Cas et al, 2015; Vitalini et al, 2015; Petalka et al, 2020	4	X			X		X			
Herniaria glabra L.	Caryophyllaceae	Erniaria glabra	w	0.03	0.44	Leporatti and Ivancheva, 2003; Petalka et al, 2020	2				X	X	X			X
Hesperis matronalis L.	Brassicaceae	Violaciocca	w	0.01	0.56	Guarino et al, 2008	1		X	X	X	X				X

Hippocrepis emerus (L.) Lassen	Fabaceae	Sferracavallo comune	w	0.03	0.11	De Natale and Pollio, 2007; Guarino et al, 2008	2							X		
Hordeum vulgare L.	Poaceae	Orzo selvatico	w	0.36	0.67	Bruni et al, 1997; Pieroni, 2000; Ballero et al, 2001; Palmese et al, 2001; Camangi et al, 2003; Loi et al, 2004; Maccioni et al, 2004; Pieroni et al, 2004a; Guarrera et al, 2005; Pieroni and Quave, 2005; Scherrer et al, 2005; De Natale and Pollio, 2007; Passalacqua et al, 2007; Gonzalez-Tejero et al, 2008; Guarino et al, 2008; Maxia et al, 2008; Leporatti and Ghedira, 2009; Idolo et al, 2010; Savo et al, 2011; Montesano et al, 2012; Menale and Muoio, 2014; Dei Cas et al, 2015; Vitalini et al, 2015; Menale et al, 2016; Motti and Motti, 2017; Menale et al, 2021; Danna et al, 2022	27	X	X	X	X	X			X	
Humulus lupulus L.	Cannabaceae	Luppolo	w	0.15	0.78	Lokar and Poldrini, 1988; Bruni et al, 1997; Pieroni, 2000; Leporatti and Ivancheva, 2003; Passalacqua et al, 2007; Mattalia et al, 2012; Di Novella et al, 2013; Vitalini et al, 2015; Bruschi et al, 2019; Mattalia et al, 2019; Petalka et al, 2020	11	X	X	X	X	X	X	X		
Hylocomium splendens (Hedw.) Schimp.	Hylocomiaceae	-	w	0.01	0.11	Dei Cas et al, 2015	1	X								
Hyoscyamus albus L.	Solanaceae	Giusquiamo bianco	w	0.11	0.67	Bruni et al, 1997; Uncini Manganelli and Tomei, 1999; Leporatti and Corradi, 2001; Leporatti and Ivancheva, 2003; Tuttolomondo et al, 2014; Tuttolomondo et al, 2014a; Tuttolomondo et al, 2014b; Guarrera et al, 2015	8	X	X	X		X	X		X	
Hyoscyamus niger L.	Solanaceae	Giusquiamo nero	w	0.11	0.78	Lokar and Poldrini, 1988; Bruni et al, 1997; Leporatti and Ivancheva, 2003; Guarino et al, 2008; Vitalini et al, 2009; Di Sanzo et al, 2013; Vitalini et al, 2015; Petalka et al, 2020	8	X	X	X		X	X		X	X
Hyoseris radiata L.	Compositae	Radicchio selvatico	w	0.04	0.22	Cornara et al, 2009; Tuttolomondo et al, 2014; Geraci et al, 2018	3	X			X					
Hypericum hircinum L.	Hypericaceae	Erba di San Giovanni	w	0.04	0.22	Pieroni et al, 2004a; Pieroni and Quave, 2005; Tuttolomondo et al, 2014a	3		X			X				
Hypericum maculatum Crantz	Hypericaceae	Erba di San Giovanni	w	0.01	0.44	Petalka et al, 2020	1		X	X		X		X		
Hypericum montanum L.	Hypericaceae	Erba di San Giovanni	w	0.03	0.22	Petalka et al, 2021	2							X		X
Hypericum perfoliatum L.	Hypericaceae	Erba di San Giovanni	w	0.03	0.11	Tuttolomondo et al, 2014; Gargano et al, 2018	2		X							
Hypericum perforatum L.	Hypericaceae	Erba di San Giovanni	w	0.61	1.00	Leporatti et al, 1985b; Lokar and Poldrini, 1988; Uncini Manganelli and Tomei, 1999; Camangi et al, 2003; Leporatti and Ivancheva, 2003; Loi et al, 2004; Maccioni et al, 2004; Pieroni et al, 2004b; Loi et al, 2005; Scherrer et al, 2005; De Natale and	45	X	X	X	X	X	X	X	X	X

						Pollio, 2007; Gonzalez-Tejero et al, 2008; Maxia et al, 2008; Cornara et al, 2009; Pieroni and Giusti, 2009; Signorini et al, 2009; Vitalini et al, 2009; Idolo et al, 2010; Savo et al, 2011; Leto et al, 2012; Mattalia et al, 2012; Vitalini et al, 2012; Di Novella et al, 2013; Di Sanzo et al, 2013; Cornara et al, 2014; Tuttolomondo et al, 2014; Tuttolomondo et al, 2014b; Bellia and Pieroni, 2015; Dei Cas et al, 2015; Vitalini et al, 2015; Fortini et al, 2016; Menale et al, 2016; Motti and Motti, 2017; Gargano et al, 2018; Bruschi et al, 2019; Lucchetti et al, 2019; Maruca et al, 2019; Mattalia et al, 2019; Mautone et al, 2019; Bottoni et al, 2020; Fontefrancesco and Pieroni, 2020; Mattalia et al, 2020b; Petalka et al, 2020; Mattalia et al, 2021; Danna et al, 2022										
Hypochaeris achyrophorus L.	Compositae	-	w	0.03	0.11	Geraci et al, 2018; Lucchetti et al, 2019	2				X					
Hypochaeris glabra L.	Compositae	Costolina liscia	w	0.01	0.22	Loi et al, 2004	1	X		X						
Hypochaeris radicata L.	Compositae	Costolina maggiore	w	0.04	0.22	Pieroni, 2000; Tuttolomondo et al, 2014a; Geraci et al, 2018	3			X	X					
Hyssopus officinalis L.	Lamiaceae	Issopo	c	0.07	0.44	Lokar and Poldrini, 1988; Tuttolomondo et al, 2014; Tuttolomondo et al, 2014b; Bellia and Pieroni, 2015; Menale et al, 2021	5		X	X		X	X			
Ilex aquifolium L.	Aquifoliaceae	Agrifolglio	w/c	0.07	0.67	Leporatti et al, 1985b; Bruni et al, 1997; Guarino et al, 2008; Leto et al, 2012; Petalka et al, 2020	5	X	X	X		X	X		X	
Inula conyza (Griess.) DC.	Compositae	Enula	w	0.01	0.33	Cornara et al, 2014	1	X		X					X	
Inula helenium L.	Compositae	Enula	w	0.03	0.67	Leporatti and Ivancheva, 2003; Guarino et al, 2008	2	X	X	X	X	X		X		
Iris germanica L.	Iridaceae	Giaggiolo	w/c	0.07	0.44	Lokar and Poldrini, 1988; Leporatti and Ivancheva, 2003; Motti et al, 2009; Menale et al, 2016; Motti and Motti, 2017	5	X	X		X	X				
Iris pallida Lam.	Iridaceae	Giaggiolo pallido	w	0.01	0.22	Leporatti and Ivancheva, 2003	1	X				X				
Iris pseudacorus L.	Iridaceae	Giaggiolo acquatico	w	0.03	0.22	Leporatti and Ivancheva, 2003; Di Novella et al, 2013	2	X	X							
Isatis tinctoria L.	Brassicaceae	Glasto comune	w	0.01	0.22	Guarino et al, 2008	1	X								X
Jacobaea candida (C.Presl) B.Nord. & Greuter	Compositae	Senecione bianco	w	0.01	0.11	Leto et al, 2012	1			X						
Jacobaea delphiniifolia (Vahl) Pelser & Veldkamp	Compositae	-	w	0.01	0.22	Leto et al, 2012	1		X				X			
Jasminum officinale L.	Oleaceae	Gelsomino	w	0.03	0.22	Lucchetti et al, 2019; Tuttolomondo et al, 2014b	2		X			X				
Juglans regia L.	Juglandaceae	Noce	c	0.45	1.00	Lokar and Poldrini, 1988; Bruni et al, 1997; Pieroni, 2000; Ballero et al, 2001; Leporatti and Corradi, 2001; Palmese et al, 2001; Camangi et al, 2003;	33	X	X	X	X	X	X	X	X	X

						Leporatti and Ivancheva, 2003; Pieroni et al, 2004b; Guarrera et al, 2005; Menale et al, 2006; Passalacqua et al, 2007; Guarino et al, 2008; Leporatti and Ghedira, 2009; Vitalini et al, 2009; Idolo et al, 2010; Savo et al, 2011; Mattalia et al, 2012; Montesano et al, 2012; Di Novella et al, 2013; Di Sanzo et al, 2013; Cornara et al, 2014; Menale and Muoio, 2014; Bellia and Pieroni, 2015; Vitalini et al, 2015; Menale et al, 2016; Bruschi et al, 2019; Maruca et al, 2019; Mautone et al, 2019; Fontefrancesco and Pieroni, 2020; Mattalia et al, 2020a; Menale et al, 2021; Danna et al, 2022										
Juniperus communis L.	Cupressaceae	Ginepro	w	0.35	1.00	Leporatti et al, 1985b; Lokar and Poldrini, 1988; Uncini Manganelli and Tomei, 1999; Pieroni, 2000; Leporatti and Corradi, 2001; Palmese et al, 2001; Camangi et al, 2003; Leporatti and Ivancheva, 2003; Scherrer et al, 2005; Menale et al, 2006; Gonzalez-Tejero et al, 2008; Cornara et al, 2009; Pieroni and Giusti, 2009; Vitalini et al, 2009; Vitalini et al, 2012; Di Sanzo et al, 2013; Cornara et al, 2014; Tuttolomondo et al, 2014a; Bellia and Pieroni, 2015; Dei Cas et al, 2015; Vitalini et al, 2015; Fortini et al, 2016; Bruschi et al, 2019; Mattalia et al, 2020a; Petalka et al, 2020; Danna et al, 2022	26	X	X	X	X	X	X	X	X	X
Juniperus communis var. *saxatilis* Pall.	Cupressaceae	Ginepro	w	0.03	0.56	Di Novella et al, 2013; Petalka et al, 2020	2	X	X		X	X	X			
Juniperus oxycedrus L.	Cupressaceae	Ginepro giallo	w	0.08	0.56	Bruni et al, 1997; Ballero et al, 2001; Loi et al, 2004; Cornara et al, 2009; Leporatti and Ghedira, 2009; Lucchetti et al, 2019	6	X	X		X	X			X	
Juniperus phoenicea L.	Cupressaceae	Ginepro fenicio	w	0.01	0.11	Leporatti and Ghedira, 2009	1				X					
Juniperus sabina L.	Cupressaceae	-	w	0.04	0.44	Vitalini et al, 2015; Petalka et al, 2020; Danna et al, 2022	3			X	X	X	X			
Juniperus sp.pl.	Cupressaceae	-	nd	0.01	0.22	Idolo et al, 2010	1					X			X	
Kalanchoe sp.pl.	Crassulaceae	-	nd	0.01	0.11	Menale et al, 2021	1			X						
Kickxia elatine (L.) Dumort.	Plantaginaceae	Cencio minore	w	0.01	0.11	Tuttolomondo et al, 2014	1		X							
Kickxia spuria (L.) Dumort.	Plantaginaceae	Cencio spurio	w	0.01	0.11	Tuttolomondo et al, 2014	1		X							
Knautia arvensis (L.) Coult.	Caprifoliaceae	Ambretta	w	0.03	0.33	Guarino et al, 2008; Mattalia et al, 2019	2	X	X			X				
Laburnum anagyroides Medik.	Fabaceae	Maggiociondolo	w	0.03	0.11	Leporatti and Ivancheva, 2003; Guarino et al, 2008	2	X								
Lactuca sativa L.	Compositae	Lattuga	c	0.26	0.78	Leporatti and Corradi, 2001; Pieroni et al, 2004a; Guarrera et al, 2005; Pieroni and Quave, 2005;	19	X	X	X	X	X	X	X		

						Scherrer et al, 2005; Passalacqua et al, 2007; Guarino et al, 2008; Motti et al, 2009; Savo et al, 2011; Montesano et al, 2012; Menale and Muoio, 2014; Vitalini et al, 2015; Fortini et al, 2016; Menale et al, 2016; Motti and Motti, 2017; Geraci et al, 2018; Bruschi et al, 2019; Mautone et al, 2019; Menale et al, 2021										
Lactuca serriola L.	Compositae	Lattuga selvatica	w	0.08	0.78	Uncini Manganelli and Tomei, 1999; Pieroni, 2000; Guarino et al, 2008; Savo et al, 2011; Mattalia et al, 2012; Geraci et al, 2018	6	X	X	X	X	X	X	X		
Lactuca virosa Habl.	Compositae	-	w	0.03	0.33	Guarino et al, 2008; Galuzzo et al, 2021	2		X	X			X			
Lamium album L.	Lamiaceae	Falsa ortica	w	0.14	0.89	Lokar and Poldrini, 1988; Bruni et al, 1997; Leporatti and Ivancheva, 2003; De Natale and Pollio, 2007; Guarino et al, 2008; Vitalini et al, 2012; Menale and Muoio, 2014; Bellia and Pieroni, 2015; Vitalini et al, 2015; Petalka et al, 2020	10	X	X	X	X	X	X	X		X
Lamium galeobdolon subsp. *flavidum* (F.Herm.) Á.Löve & D.Löve	Lamiaceae	-	w	0.01	0.11	Petalka et al, 2020	1				X					
Lamium maculatum (L.) L.	Lamiaceae	Falsa Ortica a macchie	w	0.01	0.22	Guarino et al, 2008	1				X			X		
Lamium purpureum L.	Lamiaceae	Falsa ortica rossa	w	0.01	0.67	Petalka et al, 2020	1			X	X	X	X		X	X
Lapsana communis L.	Compositae	Lassana	w	0.01	0.56	Guarino et al, 2008	1	X	X		X				X	X
Larix decidua Mill.	Pinaceae	Larice	w	0.14	1.00	Lokar and Poldrini, 1988; Pieroni and Giusti, 2009; Vitalini et al, 2009; Vitalini et al, 2012; Cornara et al, 2014; Bellia and Pieroni, 2015; Dei Cas et al, 2015; Vitalini et al, 2015; Petalka et al, 2020; Danna et al, 2022	10	X	X	X	X	X	X	X	X	X
Lathraea squamaria L.	Orobanchaceae	-	w	0.01	0.11	Guarino et al, 2008	1						X			
Lathyrus vernus (L.) Bernh.	Fabaceae	Cicerchia primaticcia	w	0.01	0.22	Guarino et al, 2008	1	X	X							
Laurus nobilis L.	Lauraceae	Alloro	w/c	0.76	0.89	Lokar and Poldrini, 1988; Bruni et al, 1997; Uncini Manganelli and Tomei, 1999; Pieroni, 2000; Ballero et al, 2001; Leporatti and Corradi, 2001; Palmese et al, 2001; Camangi et al, 2003; Loi et al, 2004; Pieroni et al, 2004a; Pieroni et al, 2004b; Guarrera et al, 2005; Guarrera et al, 2005a; Loi et al, 2005; Pieroni and Quave, 2005; Scherrer et al, 2005; Guarrera and Lucia, 2007; Passalacqua et al, 2007; Gonzalez-Tejero et al, 2008; Guarino et al, 2008; Maxia et al, 2008; Cornara et al, 2009; Leonti et al, 2009; Leporatti and Ghedira, 2009; Motti et al, 2009; Signorini et al, 2009; Vitalini et al, 2009; Idolo et al, 2010; Savo et al, 2011; Leto et al, 2012;	56	X	X	X	X	X	X	X	X	

						Montesano et al, 2012; Di Novella et al, 2013; Di Sanzo et al, 2013; Cornara et al, 2014; Menale and Muoio, 2014; Tuttolomondo et al, 2014; Tuttolomondo et al, 2014a; Tuttolomondo et al, 2014b; Bellia and Pieroni, 2015; Dei Cas et al, 2015; Guarrera et al, 2015; Vitalini et al, 2015; Fortini et al, 2016; Menale et al, 2016; Motti and Motti, 2017; Gargano et al, 2018; Bruschi et al, 2019; Lucchetti et al, 2019; Mattalia et al, 2019; Mautone et al, 2019; Bottoni et al, 2020; Mattalia et al, 2020b; Motti et al, 2020; Mattalia et al, 2021; Menale et al, 2021; Danna et al, 2022										
Lavandula angustifolia Mill.	Lamiaceae	Lavanda	w/c	0.27	0.78	Leporatti et al, 1985b; Camangi et al, 2003; Leporatti and Ivancheva, 2003; Pieroni et al, 2004b; Passalacqua et al, 2007; Guarrera et al, 2005; Scherrer et al, 2005; Cornara et al, 2009; Leto et al, 2012; Cornara et al, 2014; Tuttolomondo et al, 2014b; Dei Cas et al, 2015; Vitalini et al, 2015; Menale et al, 2016; Bruschi et al, 2019; Maruca et al, 2019; Mautone et al, 2019; Galuzzo et al, 2021; Menale et al, 2021; Danna et al, 2022	20	X	X	X	X	X	X		X	
Lavandula latifolia Medik.	Lamiaceae	Lavanda selvatica	w	0.01	0.11	Pieroni et al, 2004b	1		X							
Lavandula sp.pl.	Lamiaceae	-	nd	0.01	0.22	Lucchetti et al, 2019	1	X	X							
Lavandula stoechas L.	Lamiaceae	Lavanda selvatica	c	0.07	0.78	Uncini Manganelli and Tomei, 1999; Ballero et al, 2001; Loi et al, 2005; Maxia et al, 2008; Leporatti and Ghedira, 2009	5	X	X	X		X	X	X	X	
Lavatera olbia L.	Lamiaceae	Lavatera	w	0.03	0.44	Ballero et al, 2001; Signorini et al, 2009	2	X	X	X		X				
Lavatera sp.pl.	Lamiaceae	-	nd	0.01	0.11	Savo et al, 2011	1					X				
Lavatera triloba subsp. *agrigentina* (Tineo) R.Fern.	Malvaceae	-	w	0.01	0.33	Tuttolomondo et al, 2014	1	X		X		X				
Lemna minor L.	Araceae	Lenticchia d'acqua	w	0.01	0.11	Leporatti et al, 1985b	1		X							
Lens culinaris Medik.	Fabaceae	Lenticchia	c	0.03	0.22	Guarrera and Lucia, 2007; Menale et al, 2016	2				X		X			
Leontodon tuberosus L.	Compositae	Dente di leone	w	0.01	0.11	Pieroni, 2000	1							X		
Leontopodium nivale (Ten.) Huet ex Hand.-Mazz.	Compositae	Stella alpina	w	0.03	0.44	Bellia and Pieroni, 2015; Petalka et al, 2020	2	X	X	X		X				
Leonurus cardiaca L.	Lamiaceae	Cardiaca comune	w	0.03	0.44	Leporatti and Ivancheva, 2003; Petalka et al, 2020	2				X		X	X		X
Leopoldia comosa (L.) Parl.	Asparagaceae	Lampascione	w	0.15	0.67	Guarrera et al, 2005; Pieroni and Quave, 2005; Guarrera and Lucia, 2007; Guarino et al, 2008; Motti et al, 2009; Savo et al, 2011; Di Novella et al,	11	X	X	X	X		X	X		

						2013; Di Sanzo et al, 2013; Tuttolomondo et al, 2014b; Sansanelli et al, 2017; Maruca et al, 2019										
Lepidium graminifolium L. subsp. *graminifolium*	Brassicaceae	Lepidio graminifolio	w	0.01	0.11	Savo et al, 2011	1					X				
Lepidium latifolium L.	Brassicaceae	Lepidio grande	w	0.01	0.11	Ranfa and Bodesmo, 2017	1						X			
Lepidium sativum L.	Brassicaceae	Lepidio	w	0.01	0.56	Petalka et al, 2020	1	X	X	X		X	X			
Leucanthemopsis alpina (L.) Heywood	Compositae	-	w	0.03	0.67	Vitalini et al, 2015; Petalka et al, 2020	2	X		X	X	X	X	X		
Leucanthemum vulgare (Vaill.) Lam.	Compositae	Margherita selvatica	w	0.03	0.33	Cornara et al, 2009; Petalka et al, 2020	2			X	X			X		
Leucojum vernum L.	Amaryllidaceae	Camapanella	w	0.01	0.33	Lokar and Poldrini, 1988	1		X					X		X
Levisticum officinale W.D.J.Koch	Apiaceae	Levistico	w/c	0.01	0.22	Leporatti and Ivancheva, 2003	1	X			X					
Ligustrum vulgare L.	Oleaceae	Ligustro	w	0.03	0.33	Scherrer et al, 2005; Guarino et al, 2008	2	X	X	X						
Lilium bulbiferum L.	Liliaceae	Giglio arancio	w	0.04	0.67	Camangi et al, 2003; Guarino et al, 2008; Petalka et al, 2020	3	X	X	X	X		X		X	
Lilium candidum L.	Liliaceae	Giglio bianco	c	0.05	0.44	Bruni et al, 1997; Pieroni, 2000; Cornara et al, 2009; Danna et al, 2022	4		X	X	X	X				
Lilium martagon L.	Liliaceae	Giglio martagone	w	0.03	0.67	Vitalini et al, 2015; Petalka et al, 2020	2		X	X			X	X	X	X
Linaria vulgaris Mill.	Plantaginaceae	Linaria comune	w	0.11	0.56	Leporatti et al, 1985b; Lokar and Poldrini, 1988; Leporatti and Corradi, 2001; Leporatti and Ivancheva, 2003; Pieroni et al, 2004a; Pieroni et al, 2004b; Pieroni and Quave, 2005; Guarino et al, 2008	8	X	X	X	X				X	
Linum bienne Mill.	Linaceae	Lino	w	0.01	0.11	Pieroni et al, 2004b	1		X							
Linum catharticum L.	Linaceae	Lino purgante	w	0.01	0.44	Guarino et al, 2008	1	X	X	X	X					
Linum usitatissimum L.	Linaceae	Lino	c	0.55	0.89	Lokar and Poldrini, 1988; Bruni et al, 1997; Uncini Manganelli and Tomei, 1999; Pieroni, 2000; Ballero et al, 2001; Palmese et al, 2001; Camangi et al, 2003; Leporatti and Ivancheva, 2003; Guarrera et al, 2005; Loi et al, 2004; Maccioni et al, 2004; Pieroni et al, 2004b; Guarrera et al, 2005; Loi et al, 2005; Scherrer et al, 2005; Gonzalez-Tejero et al, 2008; Guarino et al, 2008; Maxia et al, 2008; Leporatti and Ghedira, 2009; Motti et al, 2009; Pieroni and Giusti, 2009; Idolo et al, 2010; Savo et al, 2011; Vitalini et al, 2012; Cornara et al, 2014; Menale and Muoio, 2014; Tuttolomondo et al, 2014a; Tuttolomondo et al, 2014b; Bellia and Pieroni, 2015; Dei Cas et al, 2015; Guarrera et al, 2015; Vitalini et al, 2015; Fortini et al, 2016; Menale et al, 2016; Motti and Motti, 2017; Bruschi et al, 2019; Lucchetti et al, 2019; Maruca et al, 2019; Bottoni et al, 2020; Menale et al, 2021; Danna et al, 2022	41	X	X	X	X	X		X	X	X

Lithospermum officinale L.	Boraginaceae	Erba perla maggiore	w	0.01	0.33	Guarino et al, 2008	1	X	X		X					
Lobularia maritima (L.) Desv.	Brassicaceae	Filigrana comune	w/c	0.04	0.56	Savo et al, 2011; Menale et al, 2016; Mautone et al, 2019	3	X		X	X	X		X		
Lolium perenne L.	Poaceae	Loglio	w	0.01	0.22	Guarino et al, 2008	1	X					X			
Lolium sp.pl.	Poaceae	-	nd	0.01	0.11	Idolo et al, 2010	1						X			
Lolium temulentum L.	Poaceae	Loglio	w	0.01	0.33	Guarino et al, 2008	1	X		X			X			
Lonicera caprifolium L.	Caprifoliaceae	Caprifoglio	w/c	0.03	0.56	Passalacqua et al, 2007; Guarino et al, 2008	2	X	X	X	X	X				
Lonicera implexa Aiton	Caprifoliaceae	Caprifoglio mediterraneo	w	0.07	0.22	Bruni et al, 1997; Palmese et al, 2001; Loi et al, 2004; Loi et al, 2005; Guarino et al, 2008	5	X			X					
Loranthus europaeus Jacq.	Loranthaceae	Vischio bruno	w	0.03	0.22	Leporatti and Corradi, 2001; Guarino et al, 2008	2						X	X		
Lotus corniculatus L.	Fabaceae	Ginestrino comune	w	0.03	0.44	Lokar and Poldrini, 1988; Guarino et al, 2008	2	X	X				X	X		
Lunaria annua L.	Brassicaceae	Medaglie di papa	w	0.03	0.22	Guarino et al, 2008; Lucchetti et al, 2019	2				X					X
Lupinus albus L.	Fabaceae	Lupino greco	w	0.09	0.44	Pieroni et al, 2004b; Pieroni and Quave, 2005; Guarino et al, 2008; Menale and Muoio, 2014; Tuttolomondo et al, 2014a; Vitalini et al, 2015; Menale et al, 2016	7	X	X					X		X
Lupinus luteus L.	Fabaceae	Lupino irsuto	w	0.01	0.11	Guarrera and Lucia, 2007	1							X		
Lupinus sp.pl.	Fabaceae	-	nd	0.01	0.33	Leporatti and Ghedira, 2009	1	X	X							X
Lycium barbarum L.	Solanaceae	Goji	c	0.01	0.11	Bruschi et al, 2019	1			X						
Lycium europaeum L.	Solanaceae	Goji	w	0.03	0.33	Leporatti and Ghedira, 2009; Tuttolomondo et al, 2014	2	X	X	X						
Lycoperdon sp.pl.	Agaricaceae	-	nd	0.01	0.11	Fortini et al, 2016	1							X		
Lycopersicon esculentum Mill.	Solanaceae	Pomodoro	w/c	0.15	0.78	Bruni et al, 1997; Uncini Manganelli and Tomei, 1999; Guarrera et al, 2005; Pieroni and Quave, 2005; Menale et al, 2006; Guarino et al, 2008; Leporatti and Ghedira, 2009; Motti et al, 2009; Menale and Muoio, 2014; Menale et al, 2016; Mautone et al, 2019	11	X	X	X	X			X	X	X
Lycopodium clavatum L.	Lycopodiaceae	Licopodio	w	0.04	0.44	Leporatti and Ivancheva, 2003; Pieroni et al, 2004b; Petalka et al, 2020	3		X			X		X		X
Lycopodium sp.pl.	Lycopodiaceae	-	nd	0.01	0.33	Lokar and Poldrini, 1988	1	X	X		X					
Lycopus europaeus L.	Lamiaceae	Erba sega	w	0.01	0.22	Lokar and Poldrini, 1988	1								X	X
Lythrum salicaria L.	Lythraceae	Salcerella	w	0.03	0.22	Di Novella et al, 2013; Vitalini et al, 2015	2	X	X							
Malus domestica Borkh.	Rosaceae	Melo domestico	w/c	0.30	0.56	Pieroni, 2000; Ballero et al, 2001; Camangi et al, 2003; Maccioni et al, 2004; Pieroni et al, 2004a; Pieroni et al, 2004b; Guarrera et al, 2005; Guarrera et al, 2005a; Pieroni and Quave, 2005; Scherrer et al, 2005; De Natale and Pollio, 2007; Guarino et al, 2008; Cornara et al, 2009; Motti et al, 2009; Cornara et al, 2014; Menale and Muoio, 2014; Guarrera et al, 2015; Vitalini et al, 2015; Menale et al, 2016; Motti	22	X	X	X	X	X				

						and Motti, 2017; Bruschi et al, 2019; Menale et al, 2021									
Malus pumila Mill.	Rosaceae	Melo domestico	w/c	0.01	0.33	Savo et al, 2011	1	X			X	X				
Malus sylvestris (L.) Mill.	Rosaceae	Melo selvatico	w	0.08	0.44	Menale et al, 2006; Idolo et al, 2010; Di Novella et al, 2013; Fortini et al, 2016; Mattalia et al, 2020b; Mattalia et al, 2021	6	X			X	X	X			
Malva alcea L.	Malvaceae	Malvone	w/c	0.01	0.22	Petalka et al, 2020	1		X				X			
Malva arborea (L.) Webb & Berthel.	Malvaceae	Malva arborea	w	0.03	0.56	Maxia et al, 2008; Cornara et al, 2009	2	X	X	X	X	X				
Malva multiflora (Cav.) Soldano, Banfi & Galasso	Malvaceae	-	w	0.01	0.44	Signorini et al, 2009	1	X	X	X		X				
Malva neglecta Wallr.	Malvaceae	Malva violetta	w	0.12	0.67	Guarrera et al, 2005; Vitalini et al, 2009; Pieroni and Giusti, 2009; Mattalia et al, 2012; Vitalini et al, 2012; Dei Cas et al, 2015; Vitalini et al, 2015; Petalka et al, 2020; Danna et al, 2022	9	X	X	X	X	X	X			
Malva nicaeensis All.	Malvaceae	-	w	0.03	0.56	Leonti et al, 2009; Tuttolomondo et al, 2014	2	X	X		X	X			X	
Malva parviflora L.	Malvaceae	Malva minore	w	0.01	0.56	Palmese et al, 2001	1	X	X	X	X				X	
Malva sp.pl.	Malvaceae	-	nd	0.04	0.78	Savo et al, 2011; Mattalia et al, 2019; Mattalia et al, 2021	3	X	X	X	X	X	X		X	
Malva sylvestris L.	Malvaceae	Malva comune	w	0.84	1.00	Leporatti et al, 1985b; Lokar and Poldrini, 1988; Bruni et al, 1997; Uncini Manganelli and Tomei, 1999; Pieroni, 2000; Ballero et al, 2001; Leporatti and Corradi, 2001; Camangi et al, 2003; Leporatti and Ivancheva, 2003; Loi et al, 2004; Maccioni et al, 2004; Pieroni et al, 2004a; Pieroni et al, 2004b; Guarrera et al, 2005; Guarrera et al, 2005a; Loi et al, 2005; Pieroni and Quave, 2005; Scherrer et al, 2005; Menale et al, 2006; De Natale and Pollio, 2007; Guarrera and Lucia, 2007; Passalacqua et al, 2007; Gonzalez-Tejero et al, 2008; Guarino et al, 2008; Maxia et al, 2008; Cornara et al, 2009; Leonti et al, 2009; Leporatti and Ghedira, 2009; Motti et al, 2009; Pieroni and Giusti, 2009; ; Signorini et al, 2009; Idolo et al, 2010; Leto et al, 2012; Mattalia et al, 2012; Montesano et al, 2012; Di Novella et al, 2013; Di Sanzo et al, 2013; Cornara et al, 2014; Menale and Muoio, 2014; Tuttolomondo et al, 2014; Tuttolomondo et al, 2014a; Tuttolomondo et al, 2014b; Bellia and Pieroni, 2015; Guarrera et al, 2015; Vitalini et al, 2015; Fortini et al, 2016; Menale et al, 2016; Motti and Motti, 2017; Ranfa and Bodesmo, 2017; Gargano et al, 2018; Bruschi et al, 2019; Lucchetti et al, 2019; Maruca et al, 2019; Mattalia et al, 2019; Mautone et al, 2019; Bottoni et	62	X	X	X	X	X	X	X	X	X

						al, 2020; Fontefrancesco and Pieroni, 2020; Mattalia et al, 2020a; Mattalia et al, 2020b; Petalka et al, 2020; Menale et al, 2021; Danna et al, 2022										
Malva thuringiaca (L.) Vis.	Malvaceae	-	w	0.01	0.33	Fortini et al, 2016	1	X				X	X			
Malva trimestris (L.) Salisb.	Malvaceae	-	w	0.01	0.22	Tuttolomondo et al, 2014	1	X		X						
Malva verticillata L.	Malvaceae	-	w	0.01	0.33	Vitalini et al, 2015	1	X			X				X	
Mandragora officinaris Mill.	Solanaceae	Mandragora	w/c	0.03	0.11	Leto et al, 2012; Tuttolomondo et al, 2014	2		X							
Marrubium incanum Desr.	Lamiaceae	Marrubio bianco	w	0.04	0.56	Leporatti et al, 1985b; Pieroni and Quave, 2005; Guarrera and Lucia, 2007	3	X		X	X			X		X
Marrubium vulgare L.	Lamiaceae	Marrubio comune	w	0.27	0.89	Bruni et al, 1997; Leporatti and Ivancheva, 2003; Loi et al, 2004; Pieroni et al, 2004a; Pieroni and Quave, 2005; Guarrera and Lucia, 2007; Passalacqua et al, 2007; Guarino et al, 2008; Leonti et al, 2009; Leporatti and Ghedira, 2009; Signorini et al, 2009; Leto et al, 2012; Montesano et al, 2012; Di Novella et al, 2013; Di Sanzo et al, 2013; Tuttolomondo et al, 2014; Tuttolomondo et al, 2014a; Tuttolomondo et al, 2014b; Petalka et al, 2020; Galuzzo et al, 2021	20	X	X	X	X	X	X	X	X	
Matricaria chamomilla L.	Compositae	Camomilla	w	0.74	1.00	Lokar and Poldrini, 1988; Bruni et al, 1997; Uncini Manganelli and Tomei, 1999; Pieroni, 2000; Ballero et al, 2001; Leporatti and Corradi, 2001; Palmese et al, 2001; Camangi et al, 2003; Leporatti and Ivancheva, 2003; Loi et al, 2004; Maccioni et al, 2004; Pieroni et al, 2004a; Pieroni et al, 2004b; Guarrera et al, 2005; Guarrera et al, 2005a; Pieroni and Quave, 2005; Scherrer et al, 2005; Menale et al, 2006; De Natale and Pollio, 2007; Passalacqua et al, 2007; Gonzalez-Tejero et al, 2008; Guarino et al, 2008; Maxia et al, 2008; Cornara et al, 2009; Leporatti and Ghedira, 2009; Motti et al, 2009; Signorini et al, 2009; Vitalini et al, 2009; Idolo et al, 2010; Savo et al, 2011; Mattalia et al, 2012; Vitalini et al, 2012; Di Novella et al, 2013; Cornara et al, 2014; Menale and Muoio, 2014; Tuttolomondo et al, 2014; Tuttolomondo et al, 2014a; Tuttolomondo et al, 2014b; Bellia and Pieroni, 2015; Dei Cas et al, 2015; Guarrera et al, 2015; Vitalini et al, 2015; Fortini et al, 2016; Menale et al, 2016; Motti and Motti, 2017; Bruschi et al, 2019; Mattalia et al, 2019; Mautone et al, 2019; Bottoni et al, 2020; Fontefrancesco and Pieroni, 2020; Mattalia et al, 2020a; Mattalia et al, 2020b; Petalka et al, 2020;	55	X	X	X	X	X	X	X	X	X

						Galuzzo et al, 2021; Mattalia et al, 2021; Menale et al, 2021										
Matricaria discoidea DC.	Compositae	Falsa camomilla	w	0.03	0.44	Vitalini et al, 2012; Danna et al, 2022	2	X	X				X		X	
Matricaria matricarioides (Less.) Porter	Compositae	Falsa camomilla	w	0.01	0.11	Vitalini et al, 2012	1						X			
Medicago arabica (L.) Huds.	Fabaceae	Erba medica araba	w	0.01	0.22	Signorini et al, 2009	1		X					X		
Medicago falcata L.	Fabaceae	-	w	0.01	0.11	Vitalini et al, 2015	1		X							
Medicago lupulina L.	Fabaceae	Erba medica	w	0.01	0.11	Lucchetti et al, 2019	1		X							
Medicago polymorpha L.	Fabaceae	-	w	0.01	0.22	Leto et al, 2012	1		X						X	
Medicago sativa L.	Fabaceae	Erba medica	w/c	0.08	0.67	Guarino et al, 2008; Leporatti and Ghedira, 2009; Dei Cas et al, 2015; Vitalini et al, 2015; Geraci et al, 2018; Lucchetti et al, 2019	6		X	X	X			X	X	X
Melaleuca alternifolia (Maiden & Betche) Cheel	Myrtaceae	-	w	0.01	0.11	Bruschi et al, 2019	1		X							
Melampyrum arvense L.	Orobanchaceae	Spigarola bianca	w	0.01	0.22	Guarino et al, 2008	1		X	X						
Melia azedarach L.	Meliaceae	Azadiracta	c	0.03	0.22	Bruni et al, 1997; Palmese et al, 2001	2	X		X						
Melilotus altissimus Thuill.	Fabaceae	Meliloto gigante	w	0.01	0.11	Guarino et al, 2008	1						X			
Melilotus officinalis (L.) Pall.	Fabaceae	Meliloto	w	0.05	0.78	Leporatti and Ivancheva, 2003; Guarino et al, 2008; Vitalini et al, 2015; Petalka et al, 2020	4	X	X	X	X	X	X			X
Melilotus sulcatus Desf.	Fabaceae	-	w	0.01	0.22	Menale et al, 2016	1		X		X					
Melissa officinalis L.	Lamiaceae	Vera melissa	c	0.45	0.89	Leporatti et al, 1985a; Lokar and Poldrini, 1988; Pieroni, 2000; Ballero et al, 2001; Camangi et al, 2003; Leporatti and Ivancheva, 2003; Pieroni et al, 2004b; Loi et al, 2005; Menale et al, 2006; Passalacqua et al, 2007; Gonzalez-Tejero et al, 2008; Guarino et al, 2008; Maxia et al, 2008; Vitalini et al, 2009; Idolo et al, 2010; Savo et al, 2011; Vitalini et al, 2012; Di Novella et al, 2013; Cornara et al, 2014; Tuttolomondo et al, 2014b; Bellia and Pieroni, 2015; Dei Cas et al, 2015; Vitalini et al, 2015; Fortini et al, 2016; Menale et al, 2016; Bruschi et al, 2019; Lucchetti et al, 2019; Mattalia et al, 2019; Fontefrancesco and Pieroni, 2020; Mattalia et al, 2020a; Galuzzo et al, 2021; Mattalia et al, 2021; Danna et al, 2022	33	X	X	X	X	X	X	X	X	
Melittis melissophyllum L.	Lamiaceae	Erba limona	w	0.01	0.22	Idolo et al, 2010	1	X				X				

Mentha aquatica L.	Lamiaceae	Menta acquatica	w	0.12	0.78	Bruni et al, 1997; Loi et al, 2004; De Natale and Pollio, 2007; Guarino et al, 2008; Leto et al, 2012; Di Sanzo et al, 2013; Tuttolomondo et al, 2014; Vitalini et al, 2015; Petalka et al, 2020	9	X	X	X		X	X	X	X	
Mentha arvensis L.	Lamiaceae	Menta dei prati	w	0.05	0.67	Dei Cas et al, 2015; Vitalini et al, 2015; Mattalia et al, 2019; Petalka et al, 2020	4	X	X	X		X	X	X		
Mentha longifolia (L.) L.	Lamiaceae	-	w	0.12	0.89	Lokar and Poldrini, 1988; Uncini Manganelli and Tomei, 1999; Vitalini et al, 2009; Vitalini et al, 2012; Bellia and Pieroni, 2015; Vitalini et al, 2015; Motti et al, 2020; Petalka et al, 2020; Danna et al, 2022	9	X	X	X	X	X	X	X	X	
Mentha piperita L.	Lamiaceae	Menta piperita	c	0.16	0.56	Uncini Manganelli and Tomei, 1999; Leporatti and Corradi, 2001; Camangi et al, 2003; Leporatti and Ivancheva, 2003; Menale et al, 2006; Gonzalez-Tejero et al, 2008; Maxia et al, 2008; Dei Cas et al, 2015; Bruschi et al, 2019; Lucchetti et al, 2019; Maruca et al, 2019; Mautone et al, 2019	12	X	X	X		X	X			
Mentha pulegium L.	Lamiaceae	Menta poleggio	w	0.15	0.89	Leporatti and Ivancheva, 2003; Guarino et al, 2008; Maxia et al, 2008; Leporatti and Ghedira, 2009; Di Sanzo et al, 2013; Tuttolomondo et al, 2014; Tuttolomondo et al, 2014a; Tuttolomondo et al, 2014b; Motti et al, 2020; Petalka et al, 2020; Menale et al, 2021	11	X	X	X	X	X	X	X	X	
Mentha rotundifolia (L.) Huds.	Lamiaceae	-	c	0.03	0.33	Loi et al, 2005; Mautone et al, 2019	2	X		X		X				
Mentha sp.pl.	Lamiaceae	-	nd	0.23	0.89	Leporatti et al, 1985b; Bruni et al, 1997; Pieroni, 2000; Loi et al, 2005; Scherrer et al, 2005; Cornara et al, 2009; Leporatti and Ghedira, 2009; Savo et al, 2011; Cornara et al, 2014; Menale and Muoio, 2014; Sansanelli and Tassoni, 2014; Menale et al, 2016; Motti and Motti, 2017; Bruschi et al, 2019; Mattalia et al, 2020a; Mattalia et al, 2020b; Galuzzo et al, 2021; Mattalia et al, 2021	18	X	X	X	X	X	X	X	X	
Mentha spicata L.	Lamiaceae	Menta comune	w	0.20	0.67	Ballero et al, 2001; Leporatti and Ivancheva, 2003; Pieroni et al, 2004a; Pieroni et al, 2004b; Pieroni and Quave, 2005; Passalacqua et al, 2007; Guarino et al, 2008; Leporatti and Ghedira, 2009; Pieroni and Giusti, 2009; Leto et al, 2012; Dei Cas et al, 2015; Fortini et al, 2016; Mautone et al, 2019; Fontefrancesco and Pieroni, 2020; Motti et al, 2020	15	X	X	X	X	X	X			
Mentha suaveolens Ehrh.	Lamiaceae	-	w	0.11	0.67	Uncini Manganelli and Tomei, 1999; Camangi et al, 2003; Pieroni et al, 2004b; De Natale and Pollio, 2007; Guarino et al, 2008; Leporatti and Ghedira, 2009; Leto et al, 2012; Tuttolomondo et al, 2014	8	X	X	X	X	X	X			
Menyanthes trifoliata L.	Menyanthaceae	Trifoglio fibrino	w	0.03	0.56	Leporatti and Ivancheva, 2003; Petalka et al, 2020	2	X		X	X	X				X

Mercurialis annua L.	Euphorbiaceae	Mercorella comune	w	0.24	0.56	Leporatti et al, 1985b; Lokar and Poldrini, 1988; Bruni et al, 1997; Camangi et al, 2003; Maccioni et al, 2004; Pieroni et al, 2004a; Guarrera et al, 2005; Pieroni and Quave, 2005; Scherrer et al, 2005; Guarino et al, 2008; Cornara et al, 2009; Leporatti and Ghedira, 2009; Cornara et al, 2014; Menale and Muoio, 2014; Fortini et al, 2016; Menale et al, 2016; Motti and Motti, 2017; Mautone et al, 2019	18	X	X	X	X				X	
Mercurialis perennis L.	Euphorbiaceae	Mercorella bastarda	w	0.03	0.22	Guarino et al, 2008; Menale and Muoio, 2014	2	X			X					
Mespilus germanica L.	Rosaceae	Nespolo	c	0.07	0.22	Pieroni, 2000; Pieroni et al, 2004b; Guarino et al, 2008; Menale and Muoio, 2014; Menale et al, 2021	5	X				X				
Micromeria graeca (L.) Benth. ex Rchb.	Lamiaceae	Issopo alpino	w	0.08	0.44	Bruni et al, 1997; Loi et al, 2004; Guarrera et al, 2005; Savo et al, 2011; Tuttolomondo et al, 2014a; Menale et al, 2016	6	X	X			X			X	
Morus alba L.	Moraceae	Gelso bianco	w/c	0.16	0.78	Leporatti and Ivancheva, 2003; Pieroni et al, 2004a; Pieroni et al, 2004b; Pieroni and Quave, 2005; Guarino et al, 2008; Leporatti and Ghedira, 2009; Savo et al, 2011; Menale and Muoio, 2014; Menale et al, 2016; Motti and Motti, 2017; Mautone et al, 2019; Motti et al, 2020	12	X	X	X	X	X		X		X
Morus nigra L.	Moraceae	Gelso nero	w/c	0.14	0.67	Bruni et al, 1997; Leporatti and Ivancheva, 2003; Pieroni et al, 2004a; Pieroni and Quave, 2005; Guarino et al, 2008; Menale and Muoio, 2014; Menale et al, 2016; Lucchetti et al, 2019; Mautone et al, 2019; Mattalia et al, 2020b	10	X	X		X	X		X		X
Mutellina adonidifolia (J.Gay) Gutermann	Apiaceae	-	w	0.01	0.44	Petalka et al, 2020	1		X		X	X	X			
Myosotis arvensis (L.) Hill	Boraginaceae	Nontiscordardimè	w	0.01	0.11	Lucchetti et al, 2019	1		X							
Myosotis sylvatica Hoffm.	Boraginaceae	Nontiscordardimè	w	0.03	0.22	Guarino et al, 2008; Fontefrancesco and Pieroni, 2020	2	X	X							
Myricaria germanica (L.) Desv.	Tamaricaceae	Tamerice montana	w	0.01	0.11	Petalka et al, 2020	1		X							
Myristica fragrans Houtt.	Myristicaceae	Noce moscata	c	0.03	0.11	Bellia and Pieroni, 2015; Danna et al, 2022	2				X					
Myrtus communis L.	Myrtaceae	Mirto	w/c	0.32	0.89	Lokar and Poldrini, 1988; Bruni et al, 1997; Uncini Manganelli and Tomei, 1999; Palmese et al, 2001; Loi et al, 2004; Guarrera et al, 2005; Loi et al, 2005; Scherrer et al, 2005; Guarrera and Lucia, 2007; Passalacqua et al, 2007; Gonzalez-Tejero et al, 2008; Guarino et al, 2008; Maxia et al, 2008; Cornara et al, 2009; Leonti et al, 2009; Leporatti and Ghedira, 2009; Signorini et al, 2009; Savo et al, 2011; Leto et al, 2012; Montesano et al, 2012; Maruca et al, 2019;	24	X	X	X	X	X	X	X	X	

						Mautone et al, 2019; Mattalia et al, 2020b; Menale et al, 2021									
Narcissus tazetta L.	Amaryllidaceae	Narciso nostrano	w	0.01	0.22	Leto et al, 2012	1						X	X		
Nasturtium officinale R.Br.	Brassicaceae	Nasturzio	w	0.28	1.00	Lokar and Poldrini, 1988; Bruni et al, 1997; Leporatti and Ivancheva, 2003; Loi et al, 2004; Maccioni et al, 2004; Guarrera et al, 2005; Guarrera et al, 2005a; Gonzalez-Tejero et al, 2008; Guarino et al, 2008; Leporatti and Ghedira, 2009; Vitalini et al, 2012; Di Novella et al, 2013; Cornara et al, 2014; Tuttolomondo et al, 2014; Tuttolomondo et al, 2014a; Tuttolomondo et al, 2014b; Dei Cas et al, 2015; Vitalini et al, 2015; Geraci et al, 2018; Mattalia et al, 2020b; Petalka et al, 2020	21	X	X	X	X	X	X	X	X	X
Nepeta cataria L.	Lamiaceae	Gattaria comune	w	0.11	0.56	Leporatti and Ivancheva, 2003; Guarino et al, 2008; Pieroni and Giusti, 2009; Tuttolomondo et al, 2014a; Vitalini et al, 2015; Mautone et al, 2019; Petalka et al, 2020; Danna et al, 2022	8	X			X	X	X	X		
Nepeta nepetella L.	Lamiaceae	Gattaia minore	w	0.01	0.33	Leporatti et al, 1985b	1	X			X		X			
Nephrolepis cordifolia (L.) C. Presl	Nephrolepidaceae	-	w	0.01	0.11	Menale et al, 2016	1		X							
Nerium oleander L.	Apocynaceae	Oleandro	w/c	0.11	0.78	Leporatti et al, 1985a; Lokar and Poldrini, 1988; Bruni et al, 1997; Camangi et al, 2003; Leporatti and Ghedira, 2009; Signorini et al, 2009; Leto et al, 2012; Tuttolomondo et al, 2014	8	X	X	X	X	X	X	X		
Nicotiana tabacum L.	Solanaceae	Tabacco	c	0.09	0.56	Leporatti et al, 1985b; Uncini Manganelli and Tomei, 1999; Palmese et al, 2001; Camangi et al, 2003; Gonzalez-Tejero et al, 2008; Fortini et al, 2016; Mautone et al, 2019	7	X	X	X		X		X		
Nigella damascena L.	Ranunculaceae	Damigella	w/c	0.04	0.22	Leporatti and Ghedira, 2009; Leto et al, 2012; Geraci et al, 2018	3	X			X					
Nigella sativa L.	Ranunculaceae	-	w	0.01	0.22	Leporatti and Ivancheva, 2003	1	X			X					
Nymphaea alba L.	Nymphaeaceae	Ninfea	w/c	0.01	0.22	Leporatti and Ivancheva, 2003	1	X					X			
Ocimum basilicum L.	Lamiaceae	Basilico	c	0.39	0.67	Leporatti et al, 1985b; Bruni et al, 1997; Pieroni, 2000; Ballero et al, 2001; Camangi et al, 2003; Pieroni et al, 2004a; Pieroni et al, 2004b; Pieroni and Quave, 2005; Passalacqua et al, 2007; Loi et al, 2005; Gonzalez-Tejero et al, 2008; Guarino et al, 2008; Maxia et al, 2008; Cornara et al, 2009; Leporatti and Ghedira, 2009; Idolo et al, 2010; Savo et al, 2011; Montesano et al, 2012; Menale and Muoio, 2014; Dei Cas et al, 2015; Vitalini et al, 2015; Fortini et al, 2016; Menale et al, 2016; Motti and Motti, 2017; Bruschi et al, 2019; Lucchetti et al, 2019; Mautone et al, 2019; Galuzzo et al, 2021; Menale et al, 2021	29	X	X	X	X	X	X			

Oenanthe pimpinelloides L.	Apiaceae	Finocchio acquatico	w	0.01	0.11	Gonzalez-Tejero et al, 2008	1	X								
Olea europaea L.	Oleaceae	Olivo	w/c	0.62	1.00	Leporatti et al, 1985a; Lokar and Poldrini, 1988; Uncini Manganelli and Tomei, 1999; Pieroni, 2000; Ballero et al, 2001; Leporatti and Corradi, 2001; Palmese et al, 2001; Camangi et al, 2003; Leporatti and Ivancheva, 2003; Loi et al, 2004; Maccioni et al, 2004; Pieroni et al, 2004a; Pieroni et al, 2004b; Guarrera et al, 2005; Guarrera et al, 2005a; Loi et al, 2005; Pieroni and Quave, 2005; Scherrer et al, 2005; Menale et al, 2006; Passalacqua et al, 2007; Gonzalez-Tejero et al, 2008; Guarino et al, 2008; Maxia et al, 2008; Cornara et al, 2009; Leonti et al, 2009; Leporatti and Ghedira, 2009; Motti et al, 2009; Signorini et al, 2009; Savo et al, 2011; Di Novella et al, 2013; Cornara et al, 2014; Menale and Muoio, 2014; Montesano et al, 2012; Guarrera et al, 2015; Vitalini et al, 2015; Fortini et al, 2016; Menale et al, 2016; Motti and Motti, 2017; Bruschi et al, 2019; Lucchetti et al, 2019; Maruca et al, 2019; Mattalia et al, 2019; Mautone et al, 2019; Mattalia et al, 2020b; Menale et al, 2021; Danna et al, 2022	46	X	X	X	X	X	X	X	X	X
Onobrychis alba (Waldst. & Kit.) Desv.	Fabaceae	Lupinella bianca	w	0.01	0.00	Guarino et al, 2008	1				X					
Onobrychis altissima Grossh.	Fabaceae	Lupinella	w	0.01	0.00	Guarino et al, 2008	1				X					
Onobrychis montana DC.	Fabaceae	-	w	0.01	0.00	Guarino et al, 2008	1				X					
Ononis spinosa L.	Fabaceae	Ononide spinosa	w	0.09	0.67	Lokar and Poldrini, 1988; Leporatti and Ivancheva, 2003; Menale et al, 2006; Guarino et al, 2008; Di Novella et al, 2013; Bellia and Pieroni, 2015; Petalka et al, 2020	7	X	X	X	X				X	X
Ononis spinosa subsp. *hircina* (Jacq.) Gams	Fabaceae	Ononide spinosa	w	0.01	0.33	Leporatti and Ivancheva, 2003	1	X	X		X					
Ononis viscosa L.	Fabaceae	Ononide appiccicosa	w	0.01	0.11	Di Sanzo et al, 2013	1	X								
Onopordum acanthium L.	Compositae	Onopordo tomentoso	w	0.01	0.11	Leporatti and Ivancheva, 2003	1									X
Opuntia ficus-indica (L.) Mill.	Cactaceae	Fico d'India	w/c	0.31	0.78	Bruni et al, 1997; Uncini Manganelli and Tomei, 1999; Ballero et al, 2001; Palmese et al, 2001; Loi et al, 2004; Loi et al, 2005; Scherrer et al, 2005; Passalacqua et al, 2007; Maxia et al, 2008; Cornara et al, 2009; Leonti et al, 2009; Signorini et al, 2009; Savo et al, 2011; Leto et al, 2012; Tuttolomondo et al, 2014; Tuttolomondo et al, 2014a; Tuttolomondo et al, 2014b; Menale et al, 2016; Motti and Motti,	23	X	X	X	X	X	X		X	

						2017; Gargano et al, 2018; Maruca et al, 2019; Mautone et al, 2019; Menale et al, 2021										
Orchis sp.pl.	Orchidaceae	-	nd	0.01	0.33	Lokar and Poldrini, 1988	1	X	X	X						
Origanum majorana L.	Lamiaceae	Maggiorana	w/c	0.12	0.67	Leporatti et al, 1985b; Uncini Manganelli and Tomei, 1999; Loi et al, 2004; Loi et al, 2005; Guarino et al, 2008; Leporatti and Ghedira, 2009; Ranfa and Bodesmo, 2017; Lucchetti et al, 2019; Mattalia et al, 2020a	9	X	X	X	X	X	X			
Origanum sp.pl.	Lamiaceae	-	nd	0.01	0.44	Galuzzo et al, 2021	1	X		X		X	X			
Origanum vulgare L.	Lamiaceae	Origano	w/c	0.27	0.89	Lokar and Poldrini, 1988; Pieroni, 2000; Leporatti and Corradi, 2001; Leporatti and Ivancheva, 2003; De Natale and Pollio, 2007; Guarino et al, 2008; Cornara et al, 2009; Idolo et al, 2010; Cornara et al, 2014; Tuttolomondo et al, 2014b; Bellia and Pieroni, 2015; Vitalini et al, 2015; Fortini et al, 2016; Bruschi et al, 2019; Lucchetti et al, 2019; Mautone et al, 2019; Petalka et al, 2020; Mattalia et al, 2021	20	X	X	X	X	X	X		X	X
Origanum vulgare subsp. *viridulum* (Martrin-Donos) Nyman	Lamiaceae	Origano	w/c	0.11	0.56	Leporatti and Ivancheva, 2003; Pieroni et al, 2004a; Pieroni and Quave, 2005; Scherrer et al, 2005; Passalacqua et al, 2007; Guarino et al, 2008; Savo et al, 2011; Leto et al, 2012; Montesano et al, 2012; Menale et al, 2016; Menale et al, 2021	8	X		X		X	X		X	
Ornithogalum umbellatum L.	Asparagaceae	Ornitogallo	w	0.01	0.11	Petalka et al, 2020	1							X		
Orobanche crenata Forssk.	Orobanchaceae	Succiamele delle fave	w	0.01	0.22	Tuttolomondo et al, 2014b	1	X				X				
Oryza sativa L.	Poaceae	Riso	c	0.07	0.33	Pieroni et al, 2004b; Gonzalez-Tejero et al, 2008; Maxia et al, 2008; Bruschi et al, 2019; Danna et al, 2022	5	X	X	X						
Oryzopsis miliacea (L.) Asch. & Schweinf.	Poaceae	Miglio	w	0.01	0.11	Tuttolomondo et al, 2014b	1	X								
Ostrya carpinifolia Scop.	Betulaceae	Carpino nero	w	0.04	0.11	Savo et al, 2011; Lucchetti et al, 2019; Mautone et al, 2019	3					X				
Oxalis acetosella L.	Oxalidaceae	Acetosella dei boschi	w	0.07	0.56	Lokar and Poldrini, 1988; Pieroni, 2000; Leporatti and Ivancheva, 2003; Dei Cas et al, 2015; Vitalini et al, 2015	5	X	X	X	X	X				
Oxalis corniculata L.	Oxalidaceae	Acetosella dei campi	w	0.01	0.22	Bruschi et al, 2019	1			X						X
Oxalis pes-caprae L.	Oxalidaceae	Acetosella gialla	w	0.01	0.11	Tuttolomondo et al, 2014b	1	X								
Paeonia officinalis L.	Paeoniaceae	Peonia selvatica	w	0.03	0.22	Leporatti and Ivancheva, 2003; Vitalini et al, 2015	2					X	X			
Pallenis spinosa (L.) Cass.	Compositae	Asterisco spinoso	w	0.01	0.33	Tuttolomondo et al, 2014	1		X		X				X	
Panax ginseng C.A.Mey.	Araliaceae	Ginseng	c	0.01	0.11	Bruschi et al, 2019	1			X						

Panicum miliaceum L.	Poaceae	Panico	w	0.04	0.56	Pieroni, 2000; Guarino et al, 2008; Vitalini et al, 2015	3	X	X	X	X	X				
Papaver rhoeas L.	Papaveraceae	Papavero	w	0.59	0.89	Leporatti et al, 1985b; Lokar and Poldrini, 1988; Bruni et al, 1997; Pieroni, 2000; Leporatti and Corradi, 2001; Palmese et al, 2001; Camangi et al, 2003; Leporatti and Ivancheva, 2003; Loi et al, 2004; Pieroni et al, 2004b; Guarrera et al, 2005; Loi et al, 2005; Pieroni and Quave, 2005; Scherrer et al, 2005; Menale et al, 2006; De Natale and Pollio, 2007; Gonzalez-Tejero et al, 2008; Guarino et al, 2008; Maxia et al, 2008; Cornara et al, 2009; Leporatti and Ghedira, 2009; Motti et al, 2009; Signorini et al, 2009; Vitalini et al, 2009; Idolo et al, 2010; Savo et al, 2011; Leto et al, 2012; Di Novella et al, 2013; Menale and Muoio, 2014; Tuttolomondo et al, 2014; Tuttolomondo et al, 2014b; Guarrera et al, 2015; Vitalini et al, 2015; Fortini et al, 2016; Menale et al, 2016; Motti and Motti, 2017; Gargano et al, 2018; Bruschi et al, 2019; Lucchetti et al, 2019; Maruca et al, 2019; Mautone et al, 2019; Mattalia et al, 2020a; Petalka et al, 2020; Menale et al, 2021	44	X	X	X	X	X	X	X		X
Papaver somniferum L.	Papaveraceae	Papavero domestico	w/c	0.16	0.44	Bruni et al, 1997; Uncini Manganelli and Tomei, 1999; Pieroni et al, 2004a; Pieroni and Quave, 2005; Guarino et al, 2008; Montesano et al, 2012; Tuttolomondo et al, 2014; Tuttolomondo et al, 2014a; Mattalia et al, 2019; Mattalia et al, 2020b; Mattalia et al, 2021; Danna et al, 2022	12	X	X			X	X			
Papaver somniferum subsp. *setigerum* (DC.) Arcang.	Papaveraceae	Papavero domestico	w/c	0.05	0.33	Uncini Manganelli and Tomei, 1999; Palmese et al, 2001; Camangi et al, 2003; Motti et al, 2009	4	X				X	X			
Parietaria judaica L.	Urticaceae	Vetriola giudaica	w	0.49	0.89	Leporatti et al, 1985a; Lokar and Poldrini, 1988; Bruni et al, 1997; Uncini Manganelli and Tomei, 1999; Pieroni, 2000; Ballero et al, 2001; Leporatti and Corradi, 2001; Palmese et al, 2001; Loi et al, 2004; Pieroni et al, 2004a; Guarrera et al, 2005; Guarrera et al, 2005a; Loi et al, 2005; Pieroni and Quave, 2005; Scherrer et al, 2005; Guarrera and Lucia, 2007; Passalacqua et al, 2007; Gonzalez-Tejero et al, 2008; Guarino et al, 2008; Maxia et al, 2008; Cornara et al, 2009; Leonti et al, 2009; Motti et al, 2009; Signorini et al, 2009; Leto et al, 2012; Tuttolomondo et al, 2014; Tuttolomondo et al, 2014a; Tuttolomondo et al, 2014b; Guarrera et al, 2015; Fortini et al, 2016; Menale et al, 2016; Motti	36	X	X	X	X	X	X	X	X	

						and Motti, 2017; Gargano et al, 2018; Maruca et al, 2019; Menale et al, 2021									
Parietaria lusitanica L.	Urticaceae	-	w	0.01	0.33	Bruni et al, 1997	1	X	X				X			
Parietaria officinalis L.	Urticaceae	Vetriola comune	w	0.24	1.00	Maccioni et al, 2004; Pieroni et al, 2004b; Passalacqua et al, 2007; Vitalini et al, 2009; Mattalia et al, 2012; Di Novella et al, 2013; Cornara et al, 2014; Menale and Muoio, 2014; Tuttolomondo et al, 2014a; Bellia and Pieroni, 2015; Vitalini et al, 2015; Fortini et al, 2016; Lucchetti et al, 2019; Mautone et al, 2019; Fontefrancesco and Pieroni, 2020; Mattalia et al, 2020a; Mattalia et al, 2020b; Danna et al, 2022	18	X	X	X	X	X	X	X	X	X
Parietaria sp.pl.	Urticaceae	-	w	0.04	0.78	Camangi et al, 2003; Idolo et al, 2010; Savo et al, 2011	3	X	X	X	X	X		X	X	
Paris quadrifolia L.	Melanthiaceae	Uva volpina	w	0.03	0.33	Guarino et al, 2008; Vitalini et al, 2015	2	X	X				X			
Parnassia palustris L.	Celastraceae	Parnassia	w	0.01	0.33	Petalka et al, 2020	1					X		X	X	
Paronychia argentea Lam.	Caryophyllaceae	Paronchia	w	0.00	0.11	Bruni et al, 1997					X					
Passiflora caerulea L.	Passifloraceae	Passiflora	c	0.03	0.11	Uncini Manganelli and Tomei, ; Bruschi et al, 2019	2						X			
Passiflora edulis Sims	Passifloraceae	Frutto della passione	c	0.03	0.33	Passalacqua et al, 2007; Menale et al, 2021	2	X	X				X			
Passiflora sp.pl.	Passifloraceae	-	nd	0.01	0.11	Bottoni et al, 2020	1						X			
Pastinaca sativa L.	Apiaceae	Pastinaca comune	w/c	0.03	0.44	Leporatti and Ivancheva, 2003; Guarino et al, 2008	2	X			X				X	X
Pastinaca umbrosa Steven	Apiaceae	-	w	0.01	0.11	Fortini et al, 2016	1	X								
Peganum harmala L.	Nitrariaceae	-	c	0.01	0.11	Leporatti and Ghedira, 2009	1						X			
Pelargonium peltatum (L.) L'Hér.	Geraniaceae	Geranio coltivato	c	0.03	0.11	Bruni et al, 1997; Menale et al, 2016	2		X							
Pelargonium sp.pl.	Geraniaceae	-	c	0.01	0.11	Danna et al, 2022	1		X							
Pelargonium zonale (L.) L'Hér. ex Aiton	Geraniaceae	Geranio zonale	c	0.04	0.22	Bellia and Pieroni, 2015; Fortini et al, 2016; Menale et al, 2016	3	X	X							
Periploca graeca L.	Apocynaceae	Periploca maggiore	c	0.01	0.33	Leporatti and Ivancheva, 2003	1	X			X			X		
Persicaria bistorta (L.) Samp.	Polygonaceae	-	w	0.04	0.44	Leporatti and Ivancheva, 2003; Mattalia et al, 2012; Bellia and Pieroni, 2015	3	X	X	X	X					
Persicaria hydropiper (L.) Delarbre	Polygonaceae	Persicaria pepe d'acqua	w	0.01	0.22	Guarino et al, 2008	1				X			X		
Persicaria lapathifolia (L.) Delarbre	Polygonaceae	Persicaria	w	0.01	0.22	Guarino et al, 2008	1				X			X		
Petasites albus (L.) Gaertn.	Compositae	Farfaraccio bianco	w	0.01	0.11	Leporatti et al, 1985b	1		X							
Petasites hybridus (L.) G.Gaertn., B.Mey. & Scherb.	Compositae	Farfaraccio maggiore	w	0.12	0.89	Lokar and Poldrini, 1988; Pieroni, 2000; Leporatti and Ivancheva, 2003; Guarino et al, 2008; Vitalini et al, 2012; Cornara et al, 2014; Ranfa and Bodesmo, 2017; Petalka et al, 2020; Danna et al, 2022	9	X	X	X	X	X	X	X		X

Petroselinum crispum (Mill.) Fuss	Apiaceae	Prezzemolo	c	0.54	0.89	Leporatti et al, 1985b; Lokar and Poldrini, 1988; Bruni et al, 1997; Uncini Manganelli and Tomei, 1999; Ballero et al, 2001; Leporatti and Corradi, 2001; Camangi et al, 2003; Leporatti and Ivancheva, 2003; Loi et al, 2004; Maccioni et al, 2004; Pieroni et al, 2004a; Guarrera et al, 2005; Loi et al, 2005; Pieroni and Quave, 2005; Scherrer et al, 2005; Guarrera and Lucia, 2007; Passalacqua et al, 2007; Gonzalez-Tejero et al, 2008; Guarino et al, 2008; Maxia et al, 2008; Cornara et al, 2009; Leporatti and Ghedira, 2009; Motti et al, 2009; Savo et al, 2011; Montesano et al, 2012; Cornara et al, 2014; Menale and Muoio, 2014; Tuttolomondo et al, 2014a; Tuttolomondo et al, 2014b; Dei Cas et al, 2015; Vitalini et al, 2015; Fortini et al, 2016; Menale et al, 2016; Motti and Motti, 2017; Bruschi et al, 2019; Lucchetti et al, 2019; Mautone et al, 2019; Galuzzo et al, 2021; Menale et al, 2021; Danna et al, 2022	40	X	X	X	X	X	X	X	X	
Petroselinum sp.pl.	Apiaceae	-	nd	0.01	0.22	Idolo et al, 2010	1	X	X							
Petunia sp.pl.	Solanaceae	-	nd	0.01	0.11	Menale et al, 2016	1	X								
Peucedanum officinale L.	Apiaceae	-	w	0.03	0.44	Leporatti and Ivancheva, 2003; Guarino et al, 2008	2	X		X	X	X				
Peucedanum oreoselinum (L.) Moench	Apiaceae	-	w	0.01	0.22	Guarino et al, 2008	1			X		X				
Peucedanum ostruthium (L.) W.D.J.Koch	Apiaceae	-	w	0.05	0.89	Lokar and Poldrini, 1988; Vitalini et al, 2015; Petalka et al, 2020; Danna et al, 2022	4	X	X	X	X	X	X		X	X
Phaseolus vulgaris L.	Fabaceae	Fagiolo	c	0.12	0.44	Pieroni, 2000; Camangi et al, 2003; Guarrera et al, 2005; Guarrera and Lucia, 2007; Passalacqua et al, 2007; Guarino et al, 2008; Menale et al, 2016; Lucchetti et al, 2019; Menale et al, 2021	9		X		X			X		X
Phlomis fruticosa L.	Lamiaceae	Salvione giallo	w	0.01	0.11	Guarrera and Lucia, 2007	1					X				
Phragmites australis (Cav.) Trin. ex Steud.	Poaceae	Cannarella	w	0.03	0.11	Bruni et al, 1997; Guarino et al, 2008	2			X						
Physalis alkekengi L.	Solanaceae	Alkekengi	w/c	0.01	0.33	Leporatti and Ivancheva, 2003	1			X	X				X	
Phytolacca americana L.	Phytolaccaceae	Fitolacca	w	0.04	0.33	Bruni et al, 1997; Uncini Manganelli and Tomei, 1999; Gonzalez-Tejero et al, 2008	3	X		X			X			
Picea abies (L.) H.Karst.	Pinaceae	Abete rosso	w	0.09	0.89	Lokar and Poldrini, 1988; Vitalini et al, 2009; Vitalini et al, 2012; Dei Cas et al, 2015; Vitalini et al, 2015; Petalka et al, 2020; Danna et al, 2022	7	X	X	X	X	X	X	X	X	
Picris hieracioides Sibth. & Sm.	Compositae	Aspraggine comune	w	0.03	0.22	Scherrer et al, 2005; Lucchetti et al, 2019	2		X		X					
Pilosella officinarum Vaill.	Compositae	Pelosella comune	w	0.11	0.78	Lokar and Poldrini, 1988; Leporatti and Ivancheva, 2003; Maccioni et al, 2004; Guarino et al, 2008;	8	X	X	X	X	X		X	X	

						Bellia and Pieroni, 2015; Vitalini et al, 2015; Bruschi et al, 2019; Petalka et al, 2020										
Pilosella piloselloides (Vill.) Soják	Compositae	Pelosella fiorentina	w	0.01	0.11	Menale et al, 2016	1				X					
Pimpinella anisum L.	Apiaceae	Anice verde	w	0.18	0.67	Lokar and Poldrini, 1988; Bruni et al, 1997; Ballero et al, 2001; Leporatti and Ivancheva, 2003; Guarrera et al, 2005; Passalacqua et al, 2007; Leporatti and Ghedira, 2009; Idolo et al, 2010; Savo et al, 2011; Dei Cas et al, 2015; Vitalini et al, 2015; Bruschi et al, 2019; Lucchetti et al, 2019	13	X		X	X	X	X		X	
Pimpinella major (L.) Huds.	Apiaceae	Tragoselino maggiore	w	0.01	0.44	Petalka et al, 2020	1			X	X	X	X			
Pimpinella saxifraga L.	Apiaceae	Tragoselino comune	w	0.04	0.44	Leporatti and Ivancheva, 2003; Di Novella et al, 2013; Petalka et al, 2020	3	X			X	X	X			
Pinguicula alpina L.	Lentibulariaceae	Erba unta alpina	w	0.04	0.44	Vitalini et al, 2009; Vitalini et al, 2015; Petalka et al, 2020	3		X	X		X	X			
Pinguicula grandiflora Lam.	Lentibulariaceae	-	w	0.01	0.11	Fontefrancesco and Pieroni, 2020	1		X							
Pinguicula leptoceras Rchb.	Lentibulariaceae	-	w	0.01	0.22	Vitalini et al, 2015	1		X	X						
Pinguicula sp.pl.	Lentibulariaceae	-	w	0.01	0.11	Danna et al, 2022	1		X							
Pinguicula vulgaris L.	Lentibulariaceae	Erba unta comune	w	0.03	0.11	Cornara et al, 2014; Bellia and Pieroni, 2015	2		X							
Pinus cembra L.	Pinaceae	Pino cembro	w	0.08	0.89	Pieroni and Giusti, 2009; Mattalia et al, 2012; Bellia and Pieroni, 2015; Vitalini et al, 2015; Petalka et al, 2020; Danna et al, 2022	6	X	X	X	X	X	X	X		X
Pinus halepensis Mill.	Pinaceae	Pino d'Aleppo	w	0.07	0.33	Maccioni et al, 2004; Scherrer et al, 2005; Passalacqua et al, 2007; Menale and Muoio, 2014; Motti and Motti, 2017	5		X			X			X	
Pinus mugo Turra	Pinaceae	Pino mugo	w	0.16	0.67	Lokar and Poldrini, 1988; Vitalini et al, 2009; Idolo et al, 2010; Mattalia et al, 2012; Vitalini et al, 2012; Bellia and Pieroni, 2015; Dei Cas et al, 2015; Vitalini et al, 2015; Bruschi et al, 2019; Fontefrancesco and Pieroni, 2020; Mattalia et al, 2020a; Petalka et al, 2020	12	X	X	X		X	X	X		
Pinus nigra J.F.Arnold	Pinaceae	Pino nero	w	0.01	0.22	Idolo et al, 2010	1		X			X				
Pinus nigra subsp. *laricio* Maire	Pinaceae	Pino nero	w	0.04	0.33	Guarrera and Lucia, 2007; Passalacqua et al, 2007; Maruca et al, 2019	3	X	X			X				
Pinus pinaster Aiton	Pinaceae	Pino marittimo	w	0.04	0.67	Uncini Manganelli and Tomei, 1999; Cornara et al, 2009; Leporatti and Ghedira, 2009	3	X	X	X	X	X			X	
Pinus pinea L.	Pinaceae	Pino domestico	w	0.08	0.33	Motti et al, 2009; Menale and Muoio, 2014; Menale et al, 2016; Motti and Motti, 2017; Lucchetti et al, 2019; Menale et al, 2021	6	X	X			X				
Pinus sp.pl.	Pinaceae	-	nd	0.05	0.33	Camangi et al, 2003; Loi et al, 2005; Cornara et al, 2014; Mattalia et al, 2019	4	X	X			X				

Pinus sylvestris L.	Pinaceae	Pino silvestre	w	0.09	0.67	Lokar and Poldrini, 1988; Leporatti and Ivancheva, 2003; Vitalini et al, 2009; Bellia and Pieroni, 2015; Vitalini et al, 2015; Petalka et al, 2020; Danna et al, 2022	7		X	X	X	X	X		X	
Pistacia lentiscus L.	Anacardiaceae	Lentisco	w	0.24	0.67	Bruni et al, 1997; Uncini Manganelli and Tomei, 1999; Ballero et al, 2001; Palmese et al, 2001; Loi et al, 2004; Guarrera et al, 2005; Loi et al, 2005; Scherrer et al, 2005; Passalacqua et al, 2007; Guarino et al, 2008; Maxia et al, 2008; Leonti et al, 2009; Leporatti and Ghedira, 2009; Signorini et al, 2009; Savo et al, 2011; Leto et al, 2012; Tuttolomondo et al, 2014a; Guarrera et al, 2015	18	X	X	X		X		X	X	
Pistacia terebinthus L.	Anacardiaceae	Terebinto	w	0.05	0.56	Bruni et al, 1997; Palmese et al, 2001; Guarino et al, 2008; Leporatti and Ghedira, 2009	4	X	X		X	X			X	
Pistacia vera L.	Anacardiaceae	Pistacchio	w	0.01	0.11	Leporatti and Ghedira, 2009	1								X	
Pisum sativum subsp. *elatius* (M.Bieb.) Asch. & Graebn.	Fabaceae	Pisello	w	0.01	0.11	Loi et al, 2005	1			X						
Plantago afra L.	Plantaginaceae	Piantaggine pulicaria	w	0.03	0.22	De Natale and Pollio, 2007; Danna et al, 2022	2		X					X		
Plantago alpina L.	Plantaginaceae	Piantaggine alpina	w	0.01	0.22	Vitalini et al, 2015	1		X					X		
Plantago coronopus L.	Plantaginaceae	Piantaggine barbata	w	0.04	0.33	Uncini Manganelli and Tomei, 1999; Tuttolomondo et al, 2014b; Ranfa and Bodesmo, 2017	3	X	X	X						
Plantago lagopus L.	Plantaginaceae	Piantaggine piede di lepre	w	0.01	0.11	Di Novella et al, 2013	1		X							
Plantago lanceolata L.	Plantaginaceae	Piantaggine lunga	w	0.50	1.00	Lokar and Poldrini, 1988; Bruni et al, 1997; Uncini Manganelli and Tomei, 1999; Pieroni, 2000; Camangi et al, 2003; Loi et al, 2004; Maccioni et al, 2004; Pieroni et al, 2004a; Pieroni et al, 2004b; Guarrera et al, 2005; Pieroni and Quave, 2005; De Natale and Pollio, 2007; Gonzalez-Tejero et al, 2008; Guarino et al, 2008; Cornara et al, 2009; Signorini et al, 2009; Vitalini et al, 2009; Mattalia et al, 2012; Cornara et al, 2014; Menale and Muoio, 2014; Bellia and Pieroni, 2015; Guarrera et al, 2015; Vitalini et al, 2015; Fortini et al, 2016; Menale et al, 2016; Motti and Motti, 2017; Lucchetti et al, 2019; Maruca et al, 2019; Mautone et al, 2019; Fontefrancesco and Pieroni, 2020; Mattalia et al, 2019; Mattalia et al, 2020a; Mattalia et al, 2020b; Petalka et al, 2020; Mattalia et al, 2021; Menale et al, 2021; Danna et al, 2022	37	X	X	X	X	X	X	X	X	X
Plantago major L.	Plantaginaceae	Piantaggine maggiore	w	0.53	1.00	Leporatti et al, 1985b; Lokar and Poldrini, 1988; Bruni et al, 1997; Uncini Manganelli and Tomei, 1999; Pieroni, 2000; Camangi et al, 2003; Maccioni	39	X	X	X	X	X	X	X	X	X

						et al, 2004; Pieroni et al, 2004a; Guarrera et al, 2005; Guarrera et al, 2005a; Pieroni and Quave, 2005; Scherrer et al, 2005; Menale et al, 2006; Guarrera and Lucia, 2007; Passalacqua et al, 2007; Gonzalez-Tejero et al, 2008; Guarino et al, 2008; Cornara et al, 2009; Vitalini et al, 2009; Idolo et al, 2010; Leto et al, 2012; Mattalia et al, 2012; Vitalini et al, 2012; Di Novella et al, 2013; Menale and Muoio, 2014; Tuttolomondo et al, 2014; Tuttolomondo et al, 2014b; Bellia and Pieroni, 2015; Dei Cas et al, 2015; Guarrera et al, 2015; Vitalini et al, 2015; Fortini et al, 2016; Menale et al, 2016; Motti and Motti, 2017; Mautone et al, 2019; Bottoni et al, 2020; Fontefrancesco and Pieroni, 2020; Petalka et al, 2020; Danna et al, 2022										
Plantago media L.	Plantaginaceae	Piantaggine media	w	0.05	1.00	Vitalini et al, 2015; Fortini et al, 2016; Petalka et al, 2020; Danna et al, 2022	4	X	X	X	X	X	X	X	X	X
Plantago sp.pl.	Plantaginaceae	-	nd	0.07	0.56	Leporatti and Corradi, 2001; Leporatti and Ivancheva, 2003; Savo et al, 2011; Di Sanzo et al, 2013; Bruschi et al, 2019	5	X	X	X		X			X	
Platanus orientalis L.	Platanaceae	Platano	w/c	0.01	0.11	Menale and Muoio, 2014	1								X	
Plumbago europaea L.	Plumbaginaceae	Plumbago	w	0.04	0.33	Palmese et al, 2001; Loi et al, 2004; Passalacqua et al, 2007	3	X	X	X						
Poa pratensis L.	Poaceae	Fienarola comune	w/c	0.01	0.11	Gonzalez-Tejero et al, 2008	1					X				
Polygala chamaebuxus L.	Polygalaceae	Poligala falso-bosso	w	0.01	0.11	Petalka et al, 2020	1						X			
Polygala nicaeensis Risso ex W.D.J.Koch	Polygalaceae	Poligala	w	0.03	0.22	Di Novella et al, 2013; Cornara et al, 2014	2			X		X				
Polygonatum odoratum (Mill.) Druce	Asparagaceae	Sigillo di Salomone	w	0.07	0.67	Leporatti et al, 1985b; Lokar and Poldrini, 1988; Leporatti and Ivancheva, 2003; Guarino et al, 2008; Petalka et al, 2020	5	X	X	X		X			X	X
Polygonum aviculare L.	Polygonaceae	Centinodio	w	0.22	1.00	Leporatti et al, 1985b; Leporatti and Ivancheva, 2003; Gonzalez-Tejero et al, 2008; Guarino et al, 2008; Leporatti and Ghedira, 2009; Savo et al, 2011; Leto et al, 2012; Di Novella et al, 2013; Tuttolomondo et al, 2014; Menale et al, 2016; Motti and Motti, 2017; Maruca et al, 2019; Mautone et al, 2019; Petalka et al, 2020; Menale et al, 2021; Danna et al, 2022	16	X	X	X	X	X	X	X	X	X
Polypodium australe Fée	Polypodiaceae	-	w	0.03	0.11	Tuttolomondo et al, 2014; Tuttolomondo et al, 2014b	2	X								
Polypodium sp.pl.	Polypodiaceae	-	nd	0.01	0.11	Passalacqua et al, 2007	1		X							
Polypodium vulgare L.	Polypodiaceae	Polipodio comune	w	0.18	0.56	Leporatti et al, 1985b; Leporatti and Corradi, 2001; Leporatti and Ivancheva, 2003; Loi et al, 2005; Mattalia et al, 2012; Tuttolomondo et al, 2014a;	13	X		X	X	X	X			

						Bellia and Pieroni, 2015; Vitalini et al, 2015; Mattalia et al, 2019; Mautone et al, 2019; Petalka et al, 2020; Danna et al, 2022										
Populus alba L.	Salicaceae	Pioppo bianco	w	0.03	0.56	Leporatti and Ivancheva, 2003; Guarino et al, 2008	2	X		X	X				X	X
Populus nigra L.	Salicaceae	Pioppo nero	w	0.05	0.67	Leporatti and Corradi, 2001; Leporatti and Ivancheva, 2003; Guarino et al, 2008; Vitalini et al, 2015	4	X	X	X		X			X	X
Populus tremula L.	Salicaceae	Pioppo tremolo	w	0.05	0.89	Leporatti and Ivancheva, 2003; Guarino et al, 2008; Mautone et al, 2019; Petalka et al, 2020	4	X	X	X	X	X	X		X	X
Portulaca oleracea L.	Portulacaceae	Porcellana comune	w	0.16	0.56	Lokar and Poldrini, 1988; Passalacqua et al, 2007; Guarino et al, 2008; Leporatti and Ghedira, 2009; Savo et al, 2011; Di Novella et al, 2013; Di Sanzo et al, 2013; Tuttolomondo et al, 2014a; Fortini et al, 2016; Menale et al, 2016; Gargano et al, 2018; Lucchetti et al, 2019	12	X	X	X	X					X
Potentilla anserina L.	Rosaceae	-	w	0.04	0.67	Leporatti and Ivancheva, 2003; Fontefrancesco and Pieroni, 2020; Petalka et al, 2020	3	X		X	X	X		X		X
Potentilla aurea L.	Rosaceae	Cinquefoglia dorata	w	0.01	0.33	Petalka et al, 2020	1	X			X	X				
Potentilla detommasii Ten.	Rosaceae	-	w	0.01	0.33	Guarino et al, 2008	1	X		X				X		
Potentilla erecta (L.) Raeusch.	Rosaceae	Cinquefoglia tormentilla	w	0.04	0.56	Leporatti and Ivancheva, 2003; Vitalini et al, 2015; Petalka et al, 2020	3	X	X	X	X	X				
Potentilla grandiflora L.	Rosaceae	Cinquefoglia trifogliata	w	0.01	0.11	Petalka et al, 2020	1	X								
Potentilla micrantha Ramond ex DC.	Rosaceae	Cinquefoglia fragolina	w	0.01	0.33	Guarino et al, 2008	1	X		X				X		
Potentilla reptans L.	Rosaceae	Cinquefoglia comune	w	0.14	0.67	Leporatti and Ivancheva, 2003; Pieroni et al, 2004a; Pieroni and Quave, 2005; De Natale and Pollio, 2007; Gonzalez-Tejero et al, 2008; Guarino et al, 2008; Di Novella et al, 2013; Bellia and Pieroni, 2015; Menale et al, 2016; Petalka et al, 2020	10	X		X	X			X	X	X
Potentilla sp.pl.	Rosaceae	-	nd	0.01	0.44	Lokar and Poldrini, 1988	1	X			X			X	X	
Primula auricula L.	Primulaceae	Primula orecchia d'orso	w	0.01	0.22	Petalka et al, 2020	1		X				X			
Primula elatior (L.) Hill	Primulaceae	-	w	0.04	0.78	Lokar and Poldrini, 1988; Bellia and Pieroni, 2015; Petalka et al, 2020	3	X	X		X	X	X	X		X
Primula farinosa L.	Primulaceae	Primula farinosa	w	0.01	0.11	Petalka et al, 2020	1									X
Primula glutinosa Wulfen	Primulaceae	Primula vischiosa	w	0.01	0.33	Petalka et al, 2020	1					X	X	X		
Primula matthioli (L.) K.Richt.	Primulaceae	-	w	0.01	0.33	Petalka et al, 2020	1		X					X		X
Primula sp.pl.	Primulaceae	-	nd	0.03	0.11	Bruschi et al, 2019; Mattalia et al, 2020a	2					X				

Primula veris L.	Primulaceae	Primula odorosa	w	0.08	1.00	Lokar and Poldrini, 1988; Bellia and Pieroni, 2015; Dei Cas et al, 2015; Vitalini et al, 2015; Petalka et al, 2021; Danna et al, 2022	6	X	X	X	X	X	X	X	X	X
Primula vulgaris Huds.	Primulaceae	Primula comune	w	0.12	0.89	Leporatti et al, 1985b; Lokar and Poldrini, 1988; Guarino et al, 2008; Mattalia et al, 2012; Cornara et al, 2014; Tuttolomondo et al, 2014a; Tuttolomondo et al, 2014b; Bellia and Pieroni, 2015; Petalka et al, 2021	9	X	X	X	X	X	X		X	X
Prunella laciniata (L.) L.	Lamiaceae	Prunella gialla	w	0.01	0.22	Guarino et al, 2008	1	X				X				
Prunella vulgaris L.	Lamiaceae	Prunella comune	w	0.01	0.22	Guarino et al, 2008	1	X				X				
Prunus armeniaca L.	Rosaceae	Rusticano	c	0.04	0.33	Scherrer et al, 2005; Guarrera et al, 2015; Menale et al, 2016	3	X		X		X				
Prunus avium (L.) L.	Rosaceae	Ciliegio	w/c	0.27	0.89	Pieroni, 2000; Maccioni et al, 2004; Loi et al, 2005; Scherrer et al, 2005; Menale et al, 2006; Motti et al, 2009; Savo et al, 2011; Di Novella et al, 2013; Cornara et al, 2014; Bellia and Pieroni, 2015; Vitalini et al, 2015; Fortini et al, 2016; Menale et al, 2016; Motti and Motti, 2017; Bruschi et al, 2019; Lucchetti et al, 2019; Mautone et al, 2019; Fontefrancesco and Pieroni, 2020; Petalka et al, 2020; Danna et al, 2022	20	X	X	X	X	X	X	X	X	
Prunus cerasus L.	Rosaceae	Amarena	c	0.14	0.56	Lokar and Poldrini, 1988; Pieroni, 2000; Ballero et al, 2001; Leporatti and Corradi, 2001; Menale et al, 2006; De Natale and Pollio, 2007; Guarino et al, 2008; Idolo et al, 2010; Menale and Muoio, 2014; Menale et al, 2021	10	X		X	X	X				X
Prunus domestica L.	Rosaceae	Prugno	c	0.23	0.56	Lokar and Poldrini, 1988; Uncini Manganelli and Tomei, 1999; Maccioni et al, 2004; Pieroni et al, 2004a; Guarrera et al, 2005; Pieroni and Quave, 2005; Guarino et al, 2008; Maxia et al, 2008; Savo et al, 2011; Montesano et al, 2012; Cornara et al, 2014; Bellia and Pieroni, 2015; Menale et al, 2016; Motti and Motti, 2017; Mattalia et al, 2020a; Mattalia et al, 2020b; Menale et al, 2021	17	X	X	X		X			X	
Prunus dulcis (Mill.) D.A.Webb	Rosaceae	Albicocco	c	0.23	0.89	Leporatti and Corradi, 2001; Palmese et al, 2001; Pieroni et al, 2004a; Pieroni et al, 2004b; Pieroni and Quave, 2005; Scherrer et al, 2005; De Natale and Pollio, 2007; Passalacqua et al, 2007; Guarino et al, 2008; Maxia et al, 2008; Signorini et al, 2009; Idolo et al, 2010; Bellia and Pieroni, 2015; Menale et al, 2016; Motti and Motti, 2017; Lucchetti et al, 2019; Danna et al, 2022	17	X	X	X	X	X	X	X		X
Prunus laurocerasus L.	Rosaceae	Lauroceraso	c	0.01	0.22	Pieroni, 2000	1						X	X		

Prunus persica (L.) Batsch	Rosaceae	Pesco	c	0.09	0.67	Lokar and Poldrini, 1988; Camangi et al, 2003; Passalacqua et al, 2007; Guarino et al, 2008; Leporatti and Ghedira, 2009; Guarrera et al, 2015; Vitalini et al, 2015	7	X	X	X		X	X			X
Prunus spinosa L.	Rosaceae	Prugnolo	w	0.26	0.89	Leporatti et al, 1985b; Lokar and Poldrini, 1988; Pieroni, 2000; Camangi et al, 2003; Leporatti and Ivancheva, 2003; Pieroni et al, 2004a; Loi et al, 2005; Pieroni and Quave, 2005; Scherrer et al, 2005; Guarino et al, 2008; Idolo et al, 2010; Di Novella et al, 2013; Di Sanzo et al, 2013; Cornara et al, 2014; Dei Cas et al, 2015; Vitalini et al, 2015; Fortini et al, 2016; Lucchetti et al, 2019; Petalka et al, 2020	19	X	X	X	X	X	X		X	X
Pseudotsuga menziesii (Mirb.) Franco	Pinaceae	Abete odoroso	w/c	0.01	0.22	Camangi et al, 2003	1		X	X						
Pteridium aquilinum (L.) Kuhn	Dennstaedtiaceae	Felce aquilina	w	0.09	0.67	Pieroni and Quave, 2005; Scherrer et al, 2005; Savo et al, 2011; Di Sanzo et al, 2013; Menale and Muoio, 2014; Tuttolomondo et al, 2014a; Fortini et al, 2016	7		X	X		X		X	X	X
Pulicaria dysenterica (L.) Gaertn.	Compositae	Incensaria comune	w	0.03	0.11	Guarino et al, 2008; Lucchetti et al, 2019	2	X								
Pulicaria odora (L.) Rchb.	Compositae	Incensaria comune	w	0.01	0.22	Tuttolomondo et al, 2014	1		X						X	
Pulmonaria officinalis L.	Boraginaceae	Pulmonaria	w	0.08	0.67	Lokar and Poldrini, 1988; Camangi et al, 2003; Leporatti and Ivancheva, 2003; Maccioni et al, 2004; Cornara et al, 2014; Vitalini et al, 2015	6	X	X	X	X	X	X			
Pulmonaria saccharata Mill.	Boraginaceae	Pulmonaria dolce	w	0.01	0.56	Leporatti et al, 1985b	1		X	X		X		X	X	
Pulmonaria vallarsae A. Kern.	Boraginaceae	-	w	0.01	0.44	Guarino et al, 2008	1	X	X		X	X				
Pulmonaria vallarsae subsp. *apennina* (Cristof. & Puppi) L. Cecchi & Selvi	Boraginaceae	-	w	0.01	0.11	Maruca et al, 2019	1					X				
Pulsatilla montana (Hopp) Rchb.	Ranunculaceae	Pulsatilla	w	0.01	0.22	Lokar and Poldrini, 1988	1		X	X						
Pulsatilla vernalis (L.) Mill.	Ranunculaceae	Pulsatilla primaverile	w	0.01	0.11	Vitalini et al, 2015	1						X			
Punica granatum L.	Lythraceae	Melograno	w/c	0.18	0.67	Bruni et al, 1997; Leporatti and Corradi, 2001; Leporatti and Ivancheva, 2003; Guarino et al, 2008; Leporatti and Ghedira, 2009; Savo et al, 2011; Di Novella et al, 2013; Menale et al, 2016; Lucchetti et al, 2019; Maruca et al, 2019; Mautone et al, 2019; Mattalia et al, 2021; Menale et al, 2021	13	X		X	X	X		X	X	
Pyrus amygdaliformis Vill.	Rosaceae	-	w	0.01	0.11	Passalacqua et al, 2007	1		X							

Pyrus communis L.	Rosaceae	Pero selvatico	c	0.14	0.44	Pieroni, 2000; Pieroni et al, 2004a; Guarrera et al, 2005; Pieroni and Quave, 2005; Scherrer et al, 2005; Cornara et al, 2009; Motti et al, 2009; Savo et al, 2011; Montesano et al, 2012; Motti and Motti, 2017	10	X			X	X		X		
Pyrus pyraster (L.) Burgsd.	Rosaceae	Perastro	w	0.01	0.33	Mattalia et al, 2020b	1	X				X	X			
Pyrus sp.pl.	Rosaceae	-	nd	0.01	0.11	Loi et al, 2005	1	X								
Quercus cerris L.	Fagaceae	Cerro	w	0.08	0.44	Pieroni, 2000; Camangi et al, 2003; Idolo et al, 2010; Di Sanzo et al, 2013; Guarrera et al, 2015; Fortini et al, 2016	6	X	X	X	X					
Quercus frainetto Ten.	Fagaceae	Farnetto	w	0.03	0.22	Guarrera and Lucia, 2007; Passalacqua et al, 2007	2		X			X				
Quercus ilex L.	Fagaceae	Leccio	w	0.08	0.56	Bruni et al, 1997; Loi et al, 2004; Savo et al, 2011; Di Novella et al, 2013; Lucchetti et al, 2019; Mautone et al, 2019	6	X	X	X	X	X				
Quercus petraea (Matt.) Liebl.	Fagaceae	Rovere	w	0.03	0.56	Leporatti and Ivancheva, 2003; Petalka et al, 2020	2	X	X	X		X		X		
Quercus pubescens Willd.	Fagaceae	Roverella	w	0.23	0.67	Leporatti et al, 1985a; Leporatti et al, 1985b; Bruni et al, 1997; Leporatti and Corradi, 2001; Guarrera et al, 2005; Passalacqua et al, 2007; Vitalini et al, 2009; Idolo et al, 2010; Savo et al, 2011; Di Novella et al, 2013; Guarrera et al, 2015; Fortini et al, 2016; Menale et al, 2016; Lucchetti et al, 2019; Maruca et al, 2019; Petalka et al, 2020; Menale et al, 2021	17	X	X	X	X	X				X
Quercus robur L.	Fagaceae	Farnia	w	0.07	0.56	Leporatti and Ivancheva, 2003; Gonzalez-Tejero et al, 2008; Idolo et al, 2010; Mautone et al, 2019; Petalka et al, 2020	5	X	X	X		X		X		
Quercus robur subsp. *brutia* (Ten.) O.Schwarz	Fagaceae	Farnia	w	0.01	0.22	Menale and Muoio, 2014	1	X							X	
Quercus sp.pl.	Fagaceae	-	nd	0.01	0.56	Leporatti and Ghedira, 2009	1	X	X	X	X			X		
Ranunculus acris L.	Ranunculaceae	Ranuncolo	w	0.04	0.33	Camangi et al, 2003; Dei Cas et al, 2015; Petalka et al, 2020	3		X						X	X
Ranunculus bulbosus L.	Ranunculaceae	Ranuncolo	w	0.09	0.56	Pieroni et al, 2004b; Guarino et al, 2008; Cornara et al, 2009; Leporatti and Ghedira, 2009; Signorini et al, 2009; Vitalini et al, 2015; Lucchetti et al, 2019	7	X	X	X	X				X	
Ranunculus glacialis L.	Ranunculaceae	Ranuncolo glaciale	w	0.01	0.33	Vitalini et al, 2015	1	X	X	X						
Ranunculus paludosus Poir.	Ranunculaceae	Ranuncolo di palude	w	0.01	0.22	Guarino et al, 2008	1							X	X	
Ranunculus sceleratus L.	Ranunculaceae	-	w	0.01	0.11	Pieroni et al, 2004b	1								X	
Ranunculus sp.pl.	Ranunculaceae	-	nd	0.03	0.33	Lokar and Poldrini, 1988; Leporatti and Corradi, 2001	2		X	X					X	
Ranunculus thora L.	Ranunculaceae	-	w	0.01	0.22	Vitalini et al, 2009	1			X					X	

Ranunculus velutinus Ten.	Ranunculaceae	Ranuncolo vellutato	w	0.01	0.11	Lucchetti et al, 2019	1								X	
Raphanus raphanistrum L.	Brassicaceae	Ravanello selvatico	w/c	0.07	0.56	Guarino et al, 2008; Maxia et al, 2008; Leporatti and Ghedira, 2009; Ranfa and Bodesmo, 2017; Petalka et al, 2020	5	X			X	X	X	X		
Raphanus raphanistrum subsp. *sativus* (L.) Domin	Brassicaceae	Ravanello	w/c	0.11	0.67	Bruni et al, 1997; Leporatti and Ivancheva, 2003; Loi et al, 2004; Pieroni et al, 2004b; Guarino et al, 2008; Leporatti and Ghedira, 2009; Menale and Muoio, 2014; Menale et al, 2016	8	X	X		X	X		X		X
Rapistrum rugosum (L.) All.	Brassicaceae	Miagro	w	0.01	0.11	Pieroni et al, 2004b	1		X							
Reichardia picroides (L.) Roth	Compositae	Grattalingua	w	0.09	0.78	Pieroni, 2000; Loi et al, 2004; Cornara et al, 2009; Savo et al, 2011; Tuttolomondo et al, 2014a; Geraci et al, 2018; Lucchetti et al, 2019	7	X	X	X	X	X	X			X
Reseda alba L.	Resedaceae	Reseda bianca	w	0.01	0.11	Leporatti and Ghedira, 2009	1		X							
Reseda luteola L.	Resedaceae	Reseda comune	w	0.01	0.22	Leporatti and Ghedira, 2009	1			X	X					
Rhamnus alaternus L.	Rhamnaceae	Ramno	w	0.04	0.22	Ballero et al, 2001; Leporatti and Ghedira, 2009; Leto et al, 2012	3	X	X							
Rhamnus alpina subsp. *fallax* (Boiss.) Maire & Petitm.	Rhamnaceae	Ramno alpino	w	0.01	0.33	Fortini et al, 2016	1	X	X					X		
Rheum officinale Baill.	Polygonaceae	Rabarbaro	w	0.01	0.11	Bruschi et al, 2019	1	X								
Rheum palmatum L.	Polygonaceae	-	w	0.01	0.22	Leporatti and Ivancheva, 2003	1	X		X						
Rheum rhabarbarum L.	Polygonaceae	Rabarbaro	c	0.04	0.33	Dei Cas et al, 2015; Vitalini et al, 2015; Danna et al, 2022	3	X			X			X		
Rhododendron ferrugineum L.	Ericaceae	Rododendro	w	0.05	0.78	Vitalini et al, 2012; Bellia and Pieroni, 2015; Vitalini et al, 2015; Petalka et al, 2020	4	X	X		X	X	X		X	X
Rhododendron hirsutum L.	Ericaceae	Rododendro irsuto	w	0.03	0.33	Lokar and Poldrini, 1988; Fontefrancesco and Pieroni, 2020	2	X		X	X					
Rhus coriaria L.	Anacardiaceae	Sommacco maggiore	w	0.04	0.33	Leto et al, 2012; Tuttolomondo et al, 2014; Gargano et al, 2018	3	X	X	X						
Ribes alpinum L.	Grossulariaceae	Ribes alpino	w	0.01	0.22	Vitalini et al, 2015	1	X				X				
Ribes nigrum L.	Grossulariaceae	Ribes nero	c	0.01	0.44	Leporatti and Ivancheva, 2003	1	X		X	X			X		
Ribes petraeum Wulfen	Grossulariaceae	Ribes rosso	w	0.03	0.44	Vitalini et al, 2015; Petalka et al, 2020	2	X	X			X				X
Ribes rubrum L.	Grossulariaceae	Ribes rosso	w/c	0.04	0.56	Lokar and Poldrini, 1988; Menale et al, 2006; Dei Cas et al, 2015	3	X		X	X	X			X	
Ribes uva-crispa L.	Grossulariaceae	Uva spina	w/c	0.05	0.44	Leporatti et al, 1985b; Menale et al, 2006; Fortini et al, 2016; Mattalia et al, 2021	4	X		X	X					X
Ricinus communis L.	Euphorbiaceae	Ricino	w/c	0.16	0.33	Uncini Manganelli and Tomei, 1999; Ballero et al, 2001; Leporatti and Corradi, 2001; Camangi et al, 2003; Loi et al, 2005; Guarino et al, 2008; Maxia et al, 2008; Leporatti and Ghedira, 2009; Leto et al, 2012; Tuttolomondo et al, 2014b; Menale et al, 2016; Menale et al, 2021	12	X	X		X					

Ridolfia segetum (L.) Moris	Apiaceae	-	w	0.01	0.11	Tuttolomondo et al, 2014	1	X								
Robinia pseudoacacia L.	Fabaceae	Robinia	w	0.12	0.56	Lokar and Poldrini, 1988; Leporatti and Ivancheva, 2003; Pieroni et al, 2004a; Pieroni and Quave, 2005; Guarino et al, 2008; Lucchetti et al, 2019; Mattalia et al, 2019; Mattalia et al, 2020a; Mattalia et al, 2020b	9	X	X	X		X	X			
Rosa alba L.	Rosaceae	Rosa chiara	w	0.01	0.11	Danna et al, 2022	1		X							
Rosa canina L.	Rosaceae	Rosa canina	w	0.51	1.00	Leporatti et al, 1985b; Pieroni, 2000; Leporatti and Corradi, 2001; Camangi et al, 2003; Leporatti and Ivancheva, 2003; Pieroni et al, 2004a; Pieroni et al, 2004b; Loi et al, 2005; Pieroni and Quave, 2005; Menale et al, 2006; Passalacqua et al, 2007; Gonzalez-Tejero et al, 2008; Guarino et al, 2008; Leporatti and Ghedira, 2009; Pieroni and Giusti, 2009; Vitalini et al, 2009; Idolo et al, 2010; Savo et al, 2011; Vitalini et al, 2012; Di Novella et al, 2013; Cornara et al, 2014; Tuttolomondo et al, 2014; Tuttolomondo et al, 2014a; Tuttolomondo et al, 2014b; Bellia and Pieroni, 2015; Dei Cas et al, 2015; Vitalini et al, 2015; Fortini et al, 2016; Bruschi et al, 2019; Lucchetti et al, 2019; Mattalia et al, 2019; Mautone et al, 2019; Bottoni et al, 2020; Fontefrancesco and Pieroni, 2020; Mattalia et al, 2020a; Petalka et al, 2020; Mattalia et al, 2021; Danna et al, 2022	38	X	X	X	X	X	X	X	X	X
Rosa centifolia L.	Rosaceae	-	w	0.03	0.11	Bellia and Pieroni, 2015; Danna et al, 2022	2					X				
Rosa corymbifera Borkh.	Rosaceae	-	w	0.01	0.11	Petalka et al, 2020	1		X							
Rosa damascena Herrm.	Rosaceae	-	w	0.01	0.11	Pieroni et al, 2004b	1		X							
Rosa gallica L.	Rosaceae	Rosa strisciante	w	0.01	0.33	Leporatti and Ghedira, 2009	1	X		X						X
Rosa montana Chaix ex Vill.	Rosaceae	Rosa alpina	w	0.01	0.11	Petalka et al, 2020	1		X							
Rosa pendulina L.	Rosaceae	Rosa alpina	w	0.01	0.33	Petalka et al, 2020	1			X	X		X			
Rosa sempervirens L.	Rosaceae	Rosa di San Giovanni	w	0.05	0.44	Palmese et al, 2001; Maxia et al, 2008; Menale et al, 2016; Menale et al, 2021	4	X	X				X		X	
Rosa sp.pl.	Rosaceae	-	nd	0.08	0.78	Lokar and Poldrini, 1988; Uncini Manganelli and Tomei, 1999; Pieroni et al, 2004a; Idolo et al, 2010; Menale and Muoio, 2014; Menale et al, 2016	6	X	X	X	X	X	X	X		
Rosmarinus officinalis L.	Lamiaceae	Rosmarino	w/c	0.68	1.00	Leporatti et al, 1985a; Leporatti et al, 1985b; Lokar and Poldrini, 1988; Bruni et al, 1997; Uncini Manganelli and Tomei, 1999; Pieroni, 2000; Ballero et al, 2001; Leporatti and Corradi, 2001; Palmese et al, 2001; Camangi et al, 2003; Leporatti and	51	X	X	X	X	X	X	X	X	X

						Ivancheva, 2003; Loi et al, 2004; Maccioni et al, 2004; Pieroni et al, 2004a; Pieroni et al, 2004b; Guarrera et al, 2005; Guarrera et al, 2005a; Loi et al, 2005; Pieroni and Quave, 2005; Scherrer et al, 2005; Passalacqua et al, 2007; Gonzalez-Tejero et al, 2008; Guarino et al, 2008; Maxia et al, 2008; Cornara et al, 2009; Leonti et al, 2009; Leporatti and Ghedira, 2009; Idolo et al, 2010; Savo et al, 2011; Leto et al, 2012; Montesano et al, 2012; Cornara et al, 2014; Menale and Muoio, 2014; Sansanelli and Tassoni, 2014; Tuttolomondo et al, 2014; Tuttolomondo et al, 2014b; Bellia and Pieroni, 2015; Dei Cas et al, 2015; Vitalini et al, 2015; Fortini et al, 2016; Menale et al, 2016; Motti and Motti, 2017; Bruschi et al, 2019; Lucchetti et al, 2019; Mattalia et al, 2019; Mautone et al, 2019; Bottoni et al, 2020; Galuzzo et al, 2021; Mattalia et al, 2021; Menale et al, 2021; Danna et al, 2022										
Rubia peregrina L.	Rubiaceae	Robbia	w	0.01	0.11	Leporatti et al, 1985a	1							X		
Rubia tinctorium L.	Rubiaceae	Robbia colorata	c	0.01	0.33	Leporatti and Ivancheva, 2003	1	X	X		X					
Rubus bifrons Vest	Rosaceae	-	w	0.01	0.44	Petalka et al, 2020	1		X		X	X	X			
Rubus caesius L.	Rosaceae	Rovo bluastro	w	0.03	0.22	Di Novella et al, 2013; Mautone et al, 2019	2	X	X							
Rubus idaeus L.	Rosaceae	Lampone	w	0.19	0.89	Leporatti et al, 1985b; Lokar and Poldrini, 1988; Pieroni, 2000; Leporatti and Ivancheva, 2003; Vitalini et al, 2009; Idolo et al, 2010; Vitalini et al, 2012; Di Novella et al, 2013; Dei Cas et al, 2015; Vitalini et al, 2015; Fortini et al, 2016; Geraci et al, 2018; Mattalia et al, 2019; Petalka et al, 2020	14	X	X	X	X	X	X	X		X
Rubus sp.pl.	Rosaceae	-	nd	0.04	0.67	Leporatti and Corradi, 2001; Leporatti and Ivancheva, 2003; Di Sanzo et al, 2013	3	X	X	X	X	X				X
Rubus ulmifolius Schott	Rosaceae	Rovo	w	0.53	0.89	Leporatti et al, 1985a; Leporatti et al, 1985b; Uncini Manganelli and Tomei, 1999; Pieroni, 2000; Ballero et al, 2001; Camangi et al, 2003; Loi et al, 2004; Pieroni et al, 2004a; Guarrera et al, 2005; Guarrera et al, 2005a; Pieroni and Quave, 2005; Scherrer et al, 2005; Menale et al, 2006; De Natale and Pollio, 2007; Passalacqua et al, 2007; Guarino et al, 2008; Leonti et al, 2009; Leporatti and Ghedira, 2009; Signorini et al, 2009; Idolo et al, 2010; Savo et al, 2011; Leto et al, 2012; Menale and Muoio, 2014; Tuttolomondo et al, 2014; Tuttolomondo et al, 2014a; Tuttolomondo et al, 2014b; Bellia and Pieroni, 2015; Guarrera et al, 2015; Fortini et al, 2016; Menale et al, 2016; Motti and Motti, 2017; Gargano et al, 2018; Lucchetti et al, 2019; Maruca et al, 2019; Mattalia et al, 2019; Fontefrancesco and	39	X	X	X	X	X	X	X		X

						Pieroni, 2020; Mattalia et al, 2020a; Mattalia et al, 2020b; Menale et al, 2021										
Rubus vulgaris Weihe & Nees	Rosaceae	Rovo	w	0.08	0.44	Lokar and Poldrini, 1988; Pieroni et al, 2004b; Loi et al, 2005; Leonti et al, 2009; Di Novella et al, 2013; Vitalini et al, 2015	6	X	X	X		X				
Rumex acetosa L.	Polygonaceae	Romice	w	0.20	1.00	Lokar and Poldrini, 1988; Bruni et al, 1997; Pieroni, 2000; Leporatti and Ivancheva, 2003; Menale et al, 2006; Gonzalez-Tejero et al, 2008; Guarino et al, 2008; Leporatti and Ghedira, 2009; Vitalini et al, 2012; Cornara et al, 2014; Bellia and Pieroni, 2015; Dei Cas et al, 2015; Vitalini et al, 2015; Fontefrancesco and Pieroni, 2020; Petalka et al, 2020	15	X	X	X	X	X	X	X	X	X
Rumex acetosella L.	Polygonaceae	Romice minore	w	0.04	0.44	Bruni et al, 1997; Loi et al, 2004; Mattalia et al, 2019	3	X		X	X					X
Rumex alpinus L.	Polygonaceae	Rabarbaro di monte	w	0.12	0.67	Pieroni and Giusti, 2009; Mattalia et al, 2012; Vitalini et al, 2012; Bellia and Pieroni, 2015; Dei Cas et al, 2015; Vitalini et al, 2015; Fontefrancesco and Pieroni, 2020; Petalka et al, 2020; Danna et al, 2022	9	X	X		X	X		X	X	
Rumex bucephalophorus L.	Polygonaceae	-	w	0.01	0.22	Loi et al, 2004	1	X		X						
Rumex conglomeratus Murray	Polygonaceae	-	w	0.01	0.11	Scherrer et al, 2005	1	X								
Rumex crispus L.	Polygonaceae	Romice screspo	w	0.20	0.56	Bruni et al, 1997; Uncini Manganelli and Tomei, 1999; Pieroni, 2000; Leporatti and Corradi, 2001; Loi et al, 2004; Pieroni et al, 2004b; Guarrera et al, 2005; Menale et al, 2006; Cornara et al, 2009; Motti et al, 2009; Di Novella et al, 2013; Tuttolomondo et al, 2014; Guarrera et al, 2015; Mattalia et al, 2019; Petalka et al, 2020	15	X	X	X	X	X				
Rumex obtusifolius L.	Polygonaceae	-	w	0.05	0.56	Loi et al, 2005; Cornara et al, 2009; Lucchetti et al, 2019; Mattalia et al, 2020a	4	X	X	X	X			X		
Rumex pulcher L.	Polygonaceae	Romice foglia di lira	w	0.04	0.33	Guarino et al, 2008; Tuttolomondo et al, 2014; Lucchetti et al, 2019	3	X	X					X		
Rumex sanguineus L.	Polygonaceae	Romice rosso	w	0.03	0.33	Cornara et al, 2009; Maruca et al, 2019	2	X	X		X					
Rumex scutatus L.	Polygonaceae	Romice a scudo	w	0.03	0.22	Guarino et al, 2008; Petalka et al, 2020	2		X			X				
Rumex sp.pl.	Polygonaceae	-	nd	0.03	0.22	Palmese et al, 2001; Menale et al, 2016	2		X			X				
Rumex tuberosus L.	Polygonaceae	Romice tuberoso	w	0.01	0.11	Guarino et al, 2008	1					X				
Ruscus aculeatus L.	Asparagaceae	Pungitopo	w	0.27	1.00	Lokar and Poldrini, 1988; Bruni et al, 1997; Ballero et al, 2001; Leporatti and Ivancheva, 2003; Loi et al, 2004; Guarrera et al, 2005; Loi et al, 2005; Pieroni and Quave, 2005; Pieroni et al, 2005; Scherrer et al, 2005; Guarino et al, 2008; Leonti et al, 2009; Savo et al, 2011; Di Sanzo et al, 2013; Tuttolomondo et	20	X	X	X	X	X	X	X	X	X

						al, 2014; Tuttolomondo et al, 2014a; Guarrera et al, 2015; Fortini et al, 2016; Motti and Motti, 2017; Petalka et al, 2020										
Ruta angustifolia Pers.	Rutaceae	Ruta comune	w	0.03	0.56	Cornara et al, 2009; Leporatti and Ghedira, 2009	2	X	X		X		X	X		
Ruta chalepensis L.	Rutaceae	Ruta d'Aleppo	w	0.24	1.00	Bruni et al, 1997; Pieroni, 2000; Palmese et al, 2001; Maccioni et al, 2004; Guarrera et al, 2005; Passalacqua et al, 2007; Maxia et al, 2008; Cornara et al, 2009; Leonti et al, 2009; Leporatti and Ghedira, 2009; Savo et al, 2011; Leto et al, 2012; Cornara et al, 2014; Tuttolomondo et al, 2014; Menale et al, 2016; Motti and Motti, 2017; Gargano et al, 2018; Menale et al, 2021	18	X	X	X	X	X	X	X	X	X
Ruta graveolens L.	Rutaceae	Ruta comune	w/c	0.46	0.89	Lokar and Poldrini, 1988; Bruni et al, 1997; Uncini Manganelli and Tomei, 1999; Ballero et al, 2001; Leporatti and Corradi, 2001; Leporatti and Ivancheva, 2003; Pieroni et al, 2004a; Pieroni et al, 2004b; Guarrera et al, 2005; Pieroni and Quave, 2005; Scherrer et al, 2005; Menale et al, 2006; Guarrera and Lucia, 2007; De Natale and Pollio, 2007; Passalacqua et al, 2007; Gonzalez-Tejero et al, 2008; Guarino et al, 2008; Cornara et al, 2009; Leporatti and Ghedira, 2009; Motti et al, 2009; Vitalini et al, 2009; Montesano et al, 2012; Di Novella et al, 2013; Di Sanzo et al, 2013; Menale and Muoio, 2014; Tuttolomondo et al, 2014b; Vitalini et al, 2015; Fortini et al, 2016; Bruschi et al, 2019; Lucchetti et al, 2019; Mattalia et al, 2019; Mautone et al, 2019; Mattalia et al, 2020a; Mattalia et al, 2020b	34	X	X	X	X	X	X	X	X	
Ruta montana (L.) L.	Rutaceae	Ruta montana	w	0.01	0.33	Leporatti and Ghedira, 2009	1			X	X			X		
Ruta sp.pl.	Rutaceae	-	nd	0.01	0.11	Guarrera et al, 2015	1	X								
Salix alba L.	Salicaceae	Salice bianco	w	0.16	0.89	Lokar and Poldrini, 1988; Leporatti and Corradi, 2001; Camangi et al, 2003; Leporatti and Ivancheva, 2003; Guarrera et al, 2005; Guarino et al, 2008; Idolo et al, 2010; Bellia and Pieroni, 2015; Guarrera et al, 2015; Vitalini et al, 2015; Mautone et al, 2019; Petalka et al, 2020	12	X	X	X	X	X	X		X	X
Salix babylonica L.	Salicaceae	Salice piangente	c	0.01	0.11	Danna et al, 2022	1			X						
Salix caprea L.	Salicaceae	Silicone	w	0.03	0.44	Menale et al, 2016; Petalka et al, 2020	2		X				X	X		X
Salix fragilis L.	Salicaceae	Salice fragile	w	0.01	0.22	Cornara et al, 2014	1				X					X
Salix pentandra L.	Salicaceae	Salice rosso	w	0.01	0.22	Petalka et al, 2020	1							X		X
Salix purpurea L.	Salicaceae	Salice rosso	w	0.04	0.67	Guarino et al, 2008; Mautone et al, 2019; Petalka et al, 2020	3		X	X			X	X	X	X
Salix serpyllifolia Scop.	Salicaceae	Salice strisciante	w	0.01	0.22	Petalka et al, 2020	1		X							X

Salix sp.pl.	Salicaceae	-	w	0.01	0.11	Gonzalez-Tejero et al, 2008	1		X							
Salsola kali L.	Amaranthaceae	Agretti	w	0.01	0.11	Leporatti and Ghedira, 2009	1				X					
Salsola soda L.	Amaranthaceae	Agretti	w/c	0.01	0.11	Lucchetti et al, 2019	1			X						
Salvia argentea L.	Lamiaceae	-	w	0.03	0.11	Pieroni et al, 2004a; Pieroni and Quave, 2005	2							X		
Salvia glutinosa L.	Lamiaceae	Salvia gialla	w	0.03	0.33	Pieroni et al, 2004b; Petalka et al, 2020	2	X	X			X				
Salvia officinalis L.	Lamiaceae	Salvia	w/c	0.61	1.00	Leporatti et al, 1985a; Lokar and Poldrini, 1988; Uncini Manganelli and Tomei, 1999; Pieroni, 2000; Ballero et al, 2001; Leporatti and Corradi, 2001; Camangi et al, 2003; Leporatti and Ivancheva, 2003; Loi et al, 2004; Maccioni et al, 2004; Pieroni et al, 2004a; Pieroni et al, 2004b; Guarrera et al, 2005; Guarrera et al, 2005a; Loi et al, 2005; Pieroni and Quave, 2005; Scherrer et al, 2005; Passalacqua et al, 2007; Gonzalez-Tejero et al, 2008; Guarino et al, 2008; Maxia et al, 2008; Cornara et al, 2009; Savo et al, 2011; Leto et al, 2012; Montesano et al, 2012; Cornara et al, 2014; Menale and Muoio, 2014; Tuttolomondo et al, 2014; Tuttolomondo et al, 2014b; Bellia and Pieroni, 2015; Dei Cas et al, 2015; Guarrera et al, 2015; Vitalini et al, 2015; Fortini et al, 2016; Motti and Motti, 2017; Gargano et al, 2018; Bruschi et al, 2019; Lucchetti et al, 2019; Maruca et al, 2019; Mautone et al, 2019; Fontefrancesco and Pieroni, 2020; Mattalia et al, 2020b; Galuzzo et al, 2021; Mattalia et al, 2021; Danna et al, 2022	45	X	X	X	X	X	X	X	X	X
Salvia pratensis L.	Lamiaceae	Salvia dei prati	w	0.08	0.56	Leporatti et al, 1985b; Camangi et al, 2003; Vitalini et al, 2009; Mattalia et al, 2012; Vitalini et al, 2012; Sansanelli and Tassoni, 2014; Vitalini et al, 2015	7	X	X	X	X	X				
Salvia pratensis subsp. *haematodes* (L.) Arcang.	Lamiaceae	Salvia dei prati	w	0.01	0.22	Guarino et al, 2008	1	X	X							
Salvia sclarea L.	Lamiaceae	Salvia moscatella	w	0.04	0.11	Leporatti et al, 1985b; Bruni et al, 1997; Tuttolomondo et al, 2014a	3	X								
Salvia sp.pl.	Lamiaceae	-	nd	0.04	0.78	Idolo et al, 2010; Mattalia et al, 2019; Mattalia et al, 2020a	3	X	X	X	X	X	X	X		
Salvia verbenaca L.	Lamiaceae	Salvia minore	w	0.08	0.67	Guarrera et al, 2005; Cornara et al, 2009; Tuttolomondo et al, 2014; Menale et al, 2016; Lucchetti et al, 2019; Menale et al, 2021	6	X	X		X	X	X	X		
Sambucus ebulus L.	Adoxaceae	Sambuco lebbio	w	0.09	0.89	Leporatti and Ivancheva, 2003; Pieroni et al, 2004a; Pieroni and Quave, 2005; Guarino et al, 2008; Leporatti and Ghedira, 2009; Di Sanzo et al, 2013; Petalka et al, 2020	7	X	X	X	X	X	X		X	X
Sambucus nigra L.	Adoxaceae	Sambuco	w	0.82	1.00	Leporatti et al, 1985b; Lokar and Poldrini, 1988; Bruni et al, 1997; Uncini Manganelli and Tomei,	62	X	X	X	X	X	X	X	X	X

						1999; Pieroni, 2000; Ballero et al, 2001; Leporatti and Corradi, 2001; Palmese et al, 2001; Camangi et al, 2003; Leporatti and Ivancheva, 2003; Loi et al, 2004; Maccioni et al, 2004; Pieroni et al, 2004a; Pieroni et al, 2004b; Guarrera et al, 2005; Guarrera et al, 2005a; Pieroni and Quave, 2005; Scherrer et al, 2005; Menale et al, 2006; De Natale and Pollio, 2007; Guarrera and Lucia, 2007; Passalacqua et al, 2007; Gonzalez-Tejero et al, 2008; Guarino et al, 2008; Cornara et al, 2009; Leonti et al, 2009; Leporatti and Ghedira, 2009; Signorini et al, 2009; Vitalini et al, 2009; Idolo et al, 2010; Savo et al, 2011; Leto et al, 2012; Mattalia et al, 2012; Montesano et al, 2012; Vitalini et al, 2012; Di Novella et al, 2013; Di Sanzo et al, 2013; Cornara et al, 2014; Menale and Muoio, 2014; Sansanelli and Tassoni, 2014; Tuttolomondo et al, 2014; Tuttolomondo et al, 2014a; Tuttolomondo et al, 2014b; Bellia and Pieroni, 2015; Dei Cas et al, 2015; Vitalini et al, 2015; Fortini et al, 2016; Menale et al, 2016; Motti and Motti, 2017; Sansanelli et al, 2017; Bruschi et al, 2019; Lucchetti et al, 2019; Maruca et al, 2019; Mattalia et al, 2019; Mautone et al, 2019; Fontefrancesco and Pieroni, 2020; Mattalia et al, 2020a; Mattalia et al, 2020b; Petalka et al, 2020; Mattalia et al, 2021; Menale et al, 2021; Danna et al, 2022										
Sambucus racemosa L.	Adoxaceae	Sambuco rosso	w	0.04	0.78	Vitalini et al, 2015; Fontefrancesco and Pieroni, 2020; Petalka et al, 2020	3	X	X	X	X	X	X			X
Samolus valerandi L.	Primulaceae	Lino d’acqua	w	0.01	0.33	Leporatti and Ghedira, 2009	1	X	X							X
Sanguisorba minor Scop.	Rosaceae	Sanguisorba minore	w	0.08	0.56	Bruni et al, 1997; Maccioni et al, 2004; Guarino et al, 2008; Signorini et al, 2009; Lucchetti et al, 2019; Petalka et al, 2020	6	X	X		X	X		X		
Sanguisorba minor subsp. *balearica* (Bourg. ex Nyman) Mu¤oz Garm. & C.Navarro	Rosaceae	Sanguisorba minore	w	0.01	0.22	Cornara et al, 2009	1			X	X					
Sanguisorba officinalis L.	Rosaceae	Sanguisorba	w	0.03	0.22	Leporatti and Ivancheva, 2003; Mautone et al, 2019	2	X				X				
Sanicula europaea L.	Apiaceae	Erba fragolina	w	0.01	0.22	Petalka et al, 2020	1		X							X
Santolina chamaecyparissus L.	Compositae	Crespolina cipressina	w	0.04	0.11	Pieroni et al, 2004a; Pieroni and Quave, 2005; Di Sanzo et al, 2013	3					X				
Santolina etrusca (Lacaita) Marchi & D'Amato	Compositae	Crespolina etrusca	w	0.01	0.11	Guarrera et al, 2005	1	X								

Santolina insularis (Gennari ex Fiori) Arrigoni	Compositae	Crespolina Maggiore	w	0.01	0.44	Loi et al, 2004	1		X	X		X	X			
Santolina ligustica Arrigoni	Compositae	Crespolina ligure	w	0.01	0.11	Cornara et al, 2009	1	X								
Santolina neapolitana Jord. & Fourr.	Compositae	-	w	0.01	0.11	Savo et al, 2011	1					X				
Saponaria ocymoides L.	Caryophyllaceae	Saponaria rosa	w	0.01	0.11	Vitalini et al, 2015	1									X
Saponaria officinalis L.	Caryophyllaceae	Saponaria comune	w	0.12	0.89	Leporatti et al, 1985b; Lokar and Poldrini, 1988; Ballero et al, 2001; Leporatti and Ivancheva, 2003; Loi et al, 2004; Guarino et al, 2008; Di Novella et al, 2013; Fortini et al, 2016; Petalka et al, 2020	9	X	X	X	X	X	X		X	X
Satureja hortensis L.	Lamiaceae	Santoreggia domestica	w	0.01	0.33	Leporatti and Ivancheva, 2003	1	X		X		X				
Satureja montana L.	Lamiaceae	Santoreggia montana	w	0.11	0.56	Leporatti et al, 1985b; Pieroni, 2000; Leporatti and Ivancheva, 2003; Pieroni et al, 2004b; Cornara et al, 2009; Bellia and Pieroni, 2015; Fortini et al, 2016; Lucchetti et al, 2019	8	X	X	X	X	X				
Satureja montana subsp. *variegata* (Host) P.W.Ball	Lamiaceae	Santoreggia montana	w	0.01	0.33	Lokar and Poldrini, 1988	1	X		X		X				
Scabiosa columbaria L.	Caprifoliaceae	Vedovina selvatica	w	0.01	0.22	Guarino et al, 2008	1		X			X				
Scandix pecten-veneris L.	Apiaceae	Acicula comune	w	0.01	0.33	Guarino et al, 2008	1	X		X	X					
Scilla bifolia L.	Asparagaceae	Scilla	w	0.01	0.33	Guarino et al, 2008	1				X	X		X		
Scolopendrium officinale DC.	Aspleniaceae	Lingua cervina	w	0.01	0.44	Leporatti and Ivancheva, 2003	1	X	X		X	X				
Scrophularia canina L.	Scrophulariaceae	Scrofularia comune	w	0.08	0.56	Pieroni et al, 2004a; Pieroni and Quave, 2005; Guarrera and Lucia, 2007; Passalacqua et al, 2007; Guarino et al, 2008; Idolo et al, 2010	6	X	X	X				X	X	
Scrophularia nodosa L.	Scrophulariaceae	Scrofularia nodosa	w	0.04	0.56	Guarino et al, 2008; Petalka et al, 2020; Danna et al, 2022	3	X	X	X			X	X		
Scrophularia trifoliata L.	Scrophulariaceae	Scrofularia a tre foglie	w	0.04	0.22	Bruni et al, 1997; Loi et al, 2004; Loi et al, 2005	3		X		X					
Secale cereale L.	Poaceae	Segale	c	0.04	0.44	Cornara et al, 2014; Dei Cas et al, 2015; Vitalini et al, 2015	3	X	X		X	X				
Sedum acre L.	Crassulaceae	Borracina gialla	w	0.01	0.22	Leporatti and Ivancheva, 2003	1		X					X		
Sedum album L.	Crassulaceae	Borracina bianca	w	0.03	0.11	Pieroni and Giusti, 2009; Danna et al, 2022	2		X							
Sedum atratum L.	Crassulaceae	Borracina verde	w	0.01	0.22	Petalka et al, 2020	1		X					X		
Sedum caeruleum L.	Crassulaceae	Borracina azzurra	w	0.01	0.22	Loi et al, 2004	1		X						X	
Sedum maximum (L.) Suter	Crassulaceae	Borracina maggiore	w	0.07	0.44	Lokar and Poldrini, 1988; Guarino et al, 2008; Vitalini et al, 2015; Menale et al, 2021; Danna et al, 2022	5	X	X					X	X	

Sedum roseum (L.) Scop.	Crassulaceae	-	w	0.01	0.33	Petalka et al, 2020	1		X				X	X		
Sedum rupestre L.	Crassulaceae	Borracina dele rupi	w	0.03	0.11	Pieroni et al, 2004a; Pieroni and Quave, 2005	2				X					
Sedum sp.pl.	Crassulaceae	-	nd	0.01	0.11	Ballero et al, 2001	1		X							
Sedum telephium L.	Crassulaceae	-	c	0.14	0.44	Uncini Manganelli and Tomei, 1999; Leporatti and Corradi, 2001; Maccioni et al, 2004; Pieroni et al, 2004a; Pieroni and Quave, 2005; Menale et al, 2006; Di Novella et al, 2013; Vitalini et al, 2015; Fontefrancesco and Pieroni, 2020; Petalka et al, 2020	10		X	X		X		X		
Sempervivum arachnoideum L.	Crassulaceae	Semprevivo ragnateloso	w	0.01	0.22	Leporatti et al, 1985b	1		X		X					
Sempervivum montanum L.	Crassulaceae	Semprevivo montano	w	0.05	0.89	Vitalini et al, 2012; Bellia and Pieroni, 2015; Vitalini et al, 2015; Petalka et al, 2020	4	X	X	X	X	X	X	X		X
Sempervivum sp.pl.	Crassulaceae	-	nd	0.01	0.11	Idolo et al, 2010	1		X							
Sempervivum tectorum L.	Crassulaceae	Semprevivo dei tetti	w	0.11	0.78	Lokar and Poldrini, 1988; Camangi et al, 2003; Guarrera et al, 2005; Guarino et al, 2008; Di Sanzo et al, 2013; Fortini et al, 2016; Mattalia et al, 2020a; Petalka et al, 2020	8	X	X	X	X		X		X	X
Senecio delphinifolius Vahl.	Compositae	-	w	0.01	0.11	Tuttolomondo et al, 2014	1						X			
Senecio vulgaris L.	Compositae	Senecio comune	w	0.09	0.44	Camangi et al, 2003; Leporatti and Ivancheva, 2003; Pieroni et al, 2004a; Pieroni and Quave, 2005; Guarino et al, 2008; Leporatti and Ghedira, 2009; Galuzzo et al, 2021	7	X	X		X		X			
Sicyos angulatus L.	Cucurbitaceae	Zucca matta	w	0.01	0.11	Menale et al, 2021	1	X								
Sideritis syriaca L.	Lamiaceae	-	w	0.03	0.22	Leto et al, 2012; Di Sanzo et al, 2013	2		X					X		
Silene acaulis (L.) Jacq.	Caryophyllaceae	Silene a cuscinetto	w	0.01	0.22	Petalka et al, 2020	1		X					X		
Silene flos-cuculi (L.) Greuter & Burdet	Caryophyllaceae	For di cuculo	w	0.01	0.22	Leto et al, 2012	1	X					X			
Silene italica (L.) Pers.	Caryophyllaceae	Silene comune	w	0.01	0.11	Guarrera and Lucia, 2007	1					X				
Silene latifolia Poir.	Caryophyllaceae	-	w	0.01	0.22	Cornara et al, 2014	1	X		X						
Silene vulgaris (Moench) Garcke	Caryophyllaceae	Silene comune	w	0.05	0.33	Pieroni, 2000; Tuttolomondo et al, 2014b; Bruschi et al, 2019; Petalka et al, 2020	4	X	X			X				
Silybum marianum (L.) Gaertn.	Compositae	Cardo mariano	w	0.24	0.78	Camangi et al, 2003; Leporatti and Ivancheva, 2003; Pieroni et al, 2004a; Pieroni and Quave, 2005; Passalacqua et al, 2007; Guarino et al, 2008; Leporatti and Ghedira, 2009; Idolo et al, 2010; Mattalia et al, 2012; Tuttolomondo et al, 2014b; Bellia and Pieroni, 2015; Fortini et al, 2016; Menale et al, 2016; Motti and Motti, 2017; Geraci et al, 2018; Bruschi et al, 2019; Fontefrancesco and Pieroni, 2020; Mattalia et al, 2020b	18	X	X	X	X	X	X		X	

Sinapis alba L.	Brassicaceae	Senape bianca	w	0.07	0.44	Leporatti and Ivancheva, 2003; Guarrera et al, 2005; Guarino et al, 2008; Leporatti and Ghedira, 2009; Lucchetti et al, 2019	5	X				X			X	X
Sinapis arvensis L.	Brassicaceae	Senape selvatica	w	0.03	0.22	Leporatti and Ghedira, 2009; Tuttolomondo et al, 2014b	2					X			X	
Sisymbrium officinale (L.) Scop.	Brassicaceae	Erba cornacchia	w	0.04	0.33	Lokar and Poldrini, 1988; Bruni et al, 1997; Vitalini et al, 2015	3			X	X	X				
Smilax aspera L.	Smilacaceae	Smilace	w	0.16	1.00	Bruni et al, 1997; Uncini Manganelli and Tomei, 1999; Ballero et al, 2001; Palmese et al, 2001; Loi et al, 2004; Loi et al, 2005; Guarino et al, 2008; Maxia et al, 2008; Leonti et al, 2009; Leto et al, 2012; Tuttolomondo et al, 2014; Menale et al, 2021	12	X	X	X	X	X	X	X	X	X
Solanum americanum Mill.	Solanaceae	-	w	0.24	0.78	Leporatti et al, 1985b; Lokar and Poldrini, 1988; Pieroni, 2000; Palmese et al, 2001; Leporatti and Ivancheva, 2003; Loi et al, 2004; Pieroni et al, 2004a; Pieroni and Quave, 2005; De Natale and Pollio, 2007; Guarino et al, 2008; Leporatti and Ghedira, 2009; Leto et al, 2012; Di Novella et al, 2013; Tuttolomondo et al, 2014; Tuttolomondo et al, 2014a; Menale et al, 2016; Fontefrancesco and Pieroni, 2020	18	X	X	X		X	X	X	X	
Solanum decipiens Opiz	Solanaceae	-	w	0.01	0.11	Menale and Muoio, 2014	1	X								
Solanum dulcamara L.	Solanaceae	Morella rampicante	w	0.15	0.78	Lokar and Poldrini, 1988; Leporatti and Ivancheva, 2003; Guarino et al, 2008; Idolo et al, 2010; Leto et al, 2012; Tuttolomondo et al, 2014a; Tuttolomondo et al, 2014b; Vitalini et al, 2015; Menale et al, 2016; Fontefrancesco and Pieroni, 2020; Petalka et al, 2020	11	X	X	X	X	X	X			X
Solanum linnaeanum Hepper & P.-M.L.Jaeger	Solanaceae	-	w	0.01	0.44	Palmese et al, 2001	1	X	X	X					X	
Solanum lycopersicum L.	Solanaceae	Pomodoro	c	0.05	0.44	Maxia et al, 2008; Savo et al, 2011; Motti and Motti, 2017; Menale et al, 2021	4	X	X	X	X					
Solanum melongena L.	Solanaceae	Melanzana	c	0.04	0.44	Di Sanzo et al, 2013; Menale et al, 2016; Mautone et al, 2019	3	X	X	X						X
Solanum tuberosum L.	Solanaceae	Patata	c	0.46	0.78	Uncini Manganelli and Tomei, 1999; Pieroni, 2000; Leporatti and Corradi, 2001; Palmese et al, 2001; Camangi et al, 2003; Leporatti and Ivancheva, 2003; Maccioni et al, 2004; Pieroni et al, 2004b; Guarrera et al, 2005; Guarrera et al, 2005a; Pieroni and Quave, 2005; Scherrer et al, 2005; Menale et al, 2006; Passalacqua et al, 2007; Gonzalez-Tejero et al, 2008; Guarino et al, 2008; Maxia et al, 2008; Cornara et al, 2009; Motti et al, 2009; Idolo et al,	34	X	X	X	X	X	X		X	

						2010; Savo et al, 2011; Cornara et al, 2014; Menale and Muoio, 2014; Tuttolomondo et al, 2014b; Dei Cas et al, 2015; Guarrera et al, 2015; Vitalini et al, 2015; Fortini et al, 2016; Menale et al, 2016; Motti and Motti, 2017; Bruschi et al, 2019; Lucchetti et al, 2019; Mautone et al, 2019; Danna et al, 2022										
Soldanella alpina L.	Primulaceae	Soldanella	w	0.01	0.11	Petalka et al, 2020	1							X		
Solidago virgaurea L.	Compositae	Verga d'oro	w	0.07	0.78	Leporatti et al, 1985b; Lokar and Poldrini, 1988; Leporatti and Ivancheva, 2003; Guarino et al, 2008; Petalka et al, 2020	5	X	X	X	X	X	X	X		
Sonchus arvensis L.	Compositae	Grespino comune	w	0.03	0.22	Guarrera et al, 2005; Sansanelli and Tassoni, 2014; Fortini et al, 2016	3	X		X						
Sonchus asper (L.) Hill	Compositae	Grespino spinoso	w	0.15	0.67	Pieroni, 2000; Guarrera et al, 2005; Pieroni and Quave, 2005; Savo et al, 2011; Tuttolomondo et al, 2014; Dei Cas et al, 2015; Fortini et al, 2016; Ranfa and Bodesmo, 2017; Geraci et al, 2018; Lucchetti et al, 2019; Menale et al, 2021	12	X	X	X	X	X		X		
Sonchus oleraceus (L.) L.	Compositae	Grespino comune	w	0.30	0.78	Bruni et al, 1997; Uncini Manganelli and Tomei, 1999; Loi et al, 2004; Pieroni et al, 2004a; Guarrera et al, 2005; Guarrera et al, 2005a; Pieroni and Quave, 2005; Pieroni et al, 2005; Menale et al, 2006; Guarrera and Lucia, 2007; Guarino et al, 2008; Cornara et al, 2009; Leporatti and Ghedira, 2009; Savo et al, 2011; Leto et al, 2012; Tuttolomondo et al, 2014; Tuttolomondo et al, 2014b; Fortini et al, 2016; Motti and Motti, 2017; Geraci et al, 2018; Lucchetti et al, 2019; Mattalia et al, 2020a	22	X	X	X	X	X		X		X
Sonchus tenerrimus L.	Compositae	-	w	0.09	0.56	Guarrera et al, 2005; Guarrera et al, 2005a; Savo et al, 2011; Tuttolomondo et al, 2014; Fortini et al, 2016; Motti and Motti, 2017; Geraci et al, 2018	7	X	X	X	X	X				
Sorbus aria (L.) Crantz	Rosaceae	Sorbo montano	w	0.01	0.22	Leporatti et al, 1985b	1		X	X						
Sorbus aucuparia L.	Rosaceae	Sorbo degli uccellatori	w	0.03	0.67	Leporatti and Ivancheva, 2003; Petalka et al, 2020	2	X	X		X	X	X			X
Sorbus domestica L.	Rosaceae	Sorbo domestico	w	0.20	0.56	Pieroni, 2000; Leporatti and Corradi, 2001; Leporatti and Ivancheva, 2003; Pieroni et al, 2004a; Pieroni and Quave, 2005; Pieroni et al, 2005; Guarino et al, 2008; Di Novella et al, 2013; Menale and Muoio, 2014; Guarrera et al, 2015; Menale et al, 2016; Motti and Motti, 2017; Lucchetti et al, 2019; Mautone et al, 2019; Menale et al, 2021	15	X	X	X		X		X		
Spartium junceum L.	Fabaceae	Ginestra odorosa	w	0.22	0.78	Leporatti et al, 1985b; Uncini Manganelli and Tomei, 1999; Leporatti and Corradi, 2001; Pieroni et al, 2004a; Pieroni et al, 2004b; Pieroni and Quave, 2005; Scherrer et al, 2005; Passalacqua et al, 2007;	16	X	X	X	X		X	X		X

						Guarino et al, 2008; Savo et al, 2011; Leto et al, 2012; Montesano et al, 2012; Di Sanzo et al, 2013; Mautone et al, 2019; Mattalia et al, 2020b; Menale et al, 2021										
Spinacia oleracea L.	Amaranthaceae	Spinacio	c	0.03	0.11	Bruni et al, 1997; Leporatti and Ghedira, 2009	2	X								
Stachys annua (L.) L.	Lamiaceae	Stregona annua	w	0.01	0.11	Lucchetti et al, 2019	1						X			
Stachys glutinosa L.	Lamiaceae	Stregona spinosa	w	0.01	0.11	Loi et al, 2004	1					X				
Stachys officinalis (L.) Trevis.	Lamiaceae	Stregona comune	w	0.08	0.78	Lokar and Poldrini, 1988; Leporatti and Ivancheva, 2003; Guarino et al, 2008; Di Sanzo et al, 2013; Cornara et al, 2014; Petalka et al, 2020	6	X	X	X	X	X	X	X		
Stachys recta L.	Lamiaceae	-	w	0.03	0.33	Cornara et al, 2009; Cornara et al, 2014	2	X		X	X					
Stachys sp.pl.	Lamiaceae	-	nd	0.01	0.11	Pieroni et al, 2004b	1						X			
Stachys sylvatica L.	Lamiaceae	Stregona dei boschi	w	0.01	0.22	Petalka et al, 2020	1		X							X
Stellaria media (L.) Vill.	Caryophyllaceae	Centocchio	w	0.08	0.78	Leporatti and Ivancheva, 2003; Guarino et al, 2008; Di Novella et al, 2013; Dei Cas et al, 2015; Vitalini et al, 2015; Petalka et al, 2020	6	X	X	X	X	X			X	X
Stevia rebaudiana (Bertoni) Bertoni	Compositae	Stevia	c	0.01	0.11	Bruschi et al, 2019	1			X						
Sulla coronaria (L.) Medik.	Fabaceae	Sulla	w	0.01	0.22	Ranfa and Bodesmo, 2017	1	X						X		
Symphytum officinale L.	Boraginaceae	Consolida maggiore	w	0.11	0.78	Lokar and Poldrini, 1988; Leporatti and Ivancheva, 2003; Pieroni et al, 2004b; De Natale and Pollio, 2007; Guarino et al, 2008; Bellia and Pieroni, 2015; Mattalia et al, 2020a; Petalka et al, 2020	8	X	X	X			X	X	X	X
Symphytum tuberosum L.	Boraginaceae	Consolida maggiore	w	0.03	0.00	Gonzalez-Tejero et al, 2008; Mautone et al, 2019	2		X							
Syringa vulgaris L.	Oleaceae	Lillà	c	0.01	0.33	Leporatti and Ivancheva, 2003	1	X	X	X						
Syzygium aromaticum (L.) Merr. & L.M.Perry	Myrtaceae	-	c	0.03	0.11	Pieroni et al, 2004b; Bruschi et al, 2019	2	X								
Tamarix africana Poir.	Tamaricaceae	Tamerice	w	0.01	0.11	Tuttolomondo et al, 2014	1		X							
Tamarix gallica L.	Tamaricaceae	Tamerice	w	0.03	0.44	Leporatti and Ghedira, 2009; Montesano et al, 2012	2	X	x	X	X					
Tanacetum balsamita L.	Compositae	Erba amara	w	0.05	0.56	Pieroni et al, 2004b; Guarino et al, 2008; Vitalini et al, 2015; Mautone et al, 2019	4	X	X		X	X	X			
Tanacetum balsamitoides Sch.Bip.	Compositae	Erba amara	w	0.01	0.22	Gonzalez-Tejero et al, 2008	1	X						X		
Tanacetum parthenium (L.) Sch.Bip.	Compositae	Erba amara	w	0.11	0.78	Lokar and Poldrini, 1988; Uncini Manganelli and Tomei, 1999; Guarino et al, 2008; Cornara et al, 2014; Menale and Muoio, 2014; Bruschi et al, 2019; Lucchetti et al, 2019; Mattalia et al, 2020a	8	X	X	X	X	X	X		X	
Tanacetum vulgare L.	Compositae	Erba amara selvatica	w	0.12	0.56	Leporatti and Ivancheva, 2003; Menale et al, 2006; Pieroni and Giusti, 2009; Cornara et al, 2014; Tuttolomondo et al, 2014b; Bellia and Pieroni, 2015;	9	X	X	X	X			X		

						Vitalini et al, 2015; Petalka et al, 2020; Danna et al, 2022									
Taraxacum campylodes G.E.Haglund	Compositae	Tarassaco	w	0.57	0.89	Leporatti et al, 1985b; Lokar and Poldrini, 1988; Uncini Manganelli and Tomei, 1999; Pieroni, 2000; Leporatti and Corradi, 2001; Camangi et al, 2003; Leporatti and Ivancheva, 2003; Loi et al, 2004; Maccioni et al, 2004; Pieroni et al, 2004b; Menale et al, 2006; Passalacqua et al, 2007; Gonzalez-Tejero et al, 2008; Guarino et al, 2008; Cornara et al, 2009; Signorini et al, 2009; Vitalini et al, 2009; Idolo et al, 2010; Mattalia et al, 2012; Vitalini et al, 2012; Di Sanzo et al, 2013; Cornara et al, 2014; Menale and Muoio, 2014; Sansanelli and Tassoni, 2014; Bellia and Pieroni, 2015; Dei Cas et al, 2015; Vitalini et al, 2015; Fortini et al, 2016; Menale et al, 2016; Motti and Motti, 2017; Geraci et al, 2018; Bruschi et al, 2019; Lucchetti et al, 2019; Maruca et al, 2019; Mattalia et al, 2019; Fontefrancesco and Pieroni, 2020; Mattalia et al, 2020a; Motti et al, 2020; Petalka et al, 2020; Galuzzo et al, 2021; Mattalia et al, 2021; Menale et al, 2021; Danna et al, 2022	43	X	X	X	X	X	X	X		X
Taraxacum sp.pl.	Compositae	-	nd	0.01	0.22	Bottoni et al, 2020	1			X	X					
Taxus baccata L.	Taxaceae	Tasso	w/c	0.03	0.22	Bruni et al, 1997; Menale et al, 2006	2		X		X					
Teucrium capitatum L.	Lamiaceae	Camedrio capitato	w	0.01	0.22	Leporatti and Ghedira, 2009	1		X	X						
Teucrium chamaedrys L.	Lamiaceae	Camedrio	w	0.18	0.67	Pieroni, 2000; Camangi et al, 2003; Leporatti and Ivancheva, 2003; Maccioni et al, 2004; Pieroni et al, 2004a; Pieroni and Quave, 2005; Guarino et al, 2008; Cornara et al, 2009; Savo et al, 2011; Di Sanzo et al, 2013; Cornara et al, 2014; Bellia and Pieroni, 2015; Danna et al, 2022	13	X	X	X	X	X		X		
Teucrium flavum L.	Lamiaceae	Camedrio doppio	w	0.01	0.11	Gargano et al, 2018	1								X	
Teucrium fruticans L.	Lamiaceae	-	w	0.01	0.11	Tuttolomondo et al, 2014a	1	X								
Teucrium marum L.	Lamiaceae	-	w	0.03	0.22	Bruni et al, 1997; Uncini Manganelli and Tomei, 1999	2					X	X			
Teucrium montanum L.	Lamiaceae	Camedrio montano	w	0.01	0.22	Guarino et al, 2008	1	X								X
Teucrium polium L.	Lamiaceae	Camedrio femmina	w	0.04	0.33	Bruni et al, 1997; Maccioni et al, 2004; Fortini et al, 2016	3	X	X			X				
Teucrium scordium L.	Lamiaceae	-	w	0.03	0.22	Tuttolomondo et al, 2014; Tuttolomondo et al, 2014b	2		X			X				
Thalictrum aquilegiifolium L.	Ranunculaceae	Pigamo	w	0.01	0.11	Guarino et al, 2008	1						X			
Thalictrum flavum L.	Ranunculaceae	Pigamo giallo	w	0.01	0.22	Guarino et al, 2008	1	X			X					
Thapsia garganica L.	Apiaceae	-	w	0.04	0.33	Bruni et al, 1997; Guarino et al, 2008; Leporatti and Ghedira, 2009	3						X		X	X

Thlaspi rotundifolium (L.) Gaudin	Brassicaceae	-	w	0.01	0.11	Petalka et al, 2020	1				X					
Thuja occidentalis L.	Cupressaceae	Tunia	c	0.01	0.22	Leporatti and Ivancheva, 2003	1		X		X					
Thymbra capitata (L.) Cav.	Lamiaceae	Timo arbustivo	w	0.07	0.44	Loi et al, 2005; Leporatti and Ghedira, 2009; Tuttolomondo et al, 2014; Tuttolomondo et al, 2014b; Gargano et al, 2018	5	X		X		X	X			
Thymus herba-barona Loisel.	Lamiaceae	Timo del barone	w	0.03	0.67	Bruni et al, 1997; Loi et al, 2004	2	X		X	X	X		X	X	
Thymus longicaulis C.Presl	Lamiaceae	-	w	0.07	0.22	Scherrer et al, 2005; Savo et al, 2011; Menale and Muoio, 2014; Fortini et al, 2016; Menale et al, 2016	5	X				X				
Thymus praecox subsp. *polytrichus* (A.Kern. ex Borbás) Jalas	Lamiaceae	Timo	w	0.01	0.67	Vitalini et al, 2015	1	X	X	X	X	X			X	
Thymus pulegioides L.	Lamiaceae	Timo	w	0.15	1.00	Uncini Manganelli and Tomei, 1999; Pieroni, 2000; Menale et al, 2006; Cornara et al, 2009; Pieroni and Giusti, 2009; Vitalini et al, 2009; Vitalini et al, 2012; Vitalini et al, 2015; Fortini et al, 2016; Petalka et al, 2020; Danna et al, 2022	11	X	X	X	X	X	X	X	X	X
Thymus serpyllum L.	Lamiaceae	Timo serpillo	w	0.14	0.78	Gonzalez-Tejero et al, 2008; Guarino et al, 2008; Mattalia et al, 2012; Di Novella et al, 2013; Bellia and Pieroni, 2015; Bellia and Pieroni, 2015; Dei Cas et al, 2015; Bruschi et al, 2019; Mattalia et al, 2019; Fontefrancesco and Pieroni, 2020	10	X	X	X	X	X	X		X	
Thymus sp.pl.	Lamiaceae	-	nd	0.07	0.44	Lokar and Poldrini, 1988; Leporatti and Ivancheva, 2003; Bottoni et al, 2020; Mattalia et al, 2020a; Mattalia et al, 2021	5	X		X		X		X		
Thymus vulgaris L.	Lamiaceae	Timo comune	w/c	0.11	0.78	Maccioni et al, 2004; Cornara et al, 2009; Idolo et al, 2010; Leto et al, 2012; Cornara et al, 2014; Lucchetti et al, 2019; Mautone et al, 2019; Galuzzo et al, 2021	8	X	X	X	X	X	X		X	
Thymus zygis L.	Lamiaceae	-	w	0.01	0.11	Tuttolomondo et al, 2014a	1					X				
Tilia americana L.	Malvaceae	Tiglio americano	c	0.03	0.44	Guarrera et al, 2005; Tuttolomondo et al, 2014	2		X	X		X	X			
Tilia cordata Mill.	Malvaceae	Tiglio	w	0.26	0.78	Leporatti et al, 1985b; Uncini Manganelli and Tomei, 1999; Camangi et al, 2003; Leporatti and Ivancheva, 2003; Maccioni et al, 2004; Pieroni et al, 2004a; Pieroni et al, 2004b; Pieroni and Quave, 2005; Menale et al, 2006; Mattalia et al, 2012; Di Sanzo et al, 2013; Bellia and Pieroni, 2015; Lucchetti et al, 2019; Mattalia et al, 2019; Fontefrancesco and Pieroni, 2020; Mattalia et al, 2020a; Mattalia et al, 2020b; Petalka et al, 2020; Mattalia et al, 2021	19	X	X	X	X	X	X	X		
Tilia platyphyllos Scop.	Malvaceae	Tiglio nostrano	w	0.23	0.78	Pieroni, 2000; Leporatti and Corradi, 2001; Leporatti and Ivancheva, 2003; Gonzalez-Tejero et al, 2008; Guarino et al, 2008; Vitalini et al, 2009;	17	X	X	X	X	X	X	X		

						Idolo et al, 2010; Savo et al, 2011; Vitalini et al, 2012; Cornara et al, 2014; Vitalini et al, 2015; Fortini et al, 2016; Menale et al, 2016; Maruca et al, 2019; Mautone et al, 2019; Petalka et al, 2020; Danna et al, 2022										
Tilia sp.pl.	Malvaceae	-	nd	0.05	0.67	Lokar and Poldrini, 1988; Guarrera et al, 2015; Motti and Motti, 2017; Bruschi et al, 2019	4	X	X	X		X	X	X		
Tordylium apulum L.	Apiaceae	-	w	0.01	0.11	Ranfa and Bodesmo, 2017	1		X							
Tragopogon dubius Scop.	Compositae	Barba di becco	w	0.01	0.11	Petalka et al, 2020	1		X							
Tragopogon porrifolius L.	Compositae	Barba di becco viola	w	0.03	0.44	Geraci et al, 2018; Galuzzo et al, 2021	2	X		X	X					X
Tragopogon pratensis L.	Compositae	Barba di becco comune	w	0.05	0.44	Guarrera et al, 2005; Mattalia et al, 2012; Ranfa and Bodesmo, 2017; Danna et al, 2022	4		X		X	X				X
Tribulus terrestris L.	Zygophyllaceae	Tribolo	w	0.01	0.11	Bruni et al, 1997	1	X								
Trifolium campestre Schreb.	Fabaceae	Trifoglio campestre	w	0.01	0.33	Lokar and Poldrini, 1988	1		X			X		X		
Trifolium medium L.	Fabaceae	Trifoglio	w	0.01	0.22	Vitalini et al, 2015	1				X	X				
Trifolium pallidum Waldst. & Kit.	Fabaceae	Trifoglio	w	0.01	0.22	Guarino et al, 2008	1	X			X					
Trifolium phleoides Willd.	Fabaceae	Trifoglio	w	0.01	0.11	Tuttolomondo et al, 2014a	1				X					
Trifolium pratense L.	Fabaceae	Trifoglio	w	0.12	0.67	Leporatti et al, 1985b; Leporatti and Corradi, 2001; Leporatti and Ivancheva, 2003; Guarino et al, 2008; Vitalini et al, 2015; Geraci et al, 2018; Lucchetti et al, 2019; Petalka et al, 2020; Danna et al, 2022	9	X	X	X	X	X				X
Trifolium repens L.	Fabaceae	Trifoglio bianco	w	0.05	0.56	Vitalini et al, 2015; Lucchetti et al, 2019; Petalka et al, 2020; Danna et al, 2022	4		X	X	X	X			X	
Trigonella caerulea (L.) Ser.	Fabaceae	Meliloto	w	0.01	0.11	Bellia and Pieroni, 2015	1	X								
Trigonella foenum-graecum L.	Fabaceae	Meliloto greco	w	0.08	0.89	Bruni et al, 1997; Leporatti and Ivancheva, 2003; Guarino et al, 2008; Leporatti and Ghedira, 2009; Menale et al, 2016; Geraci et al, 2018	6	X	X	X	X	X		X	X	X
Triticum aestivum L.	Poaceae	Grano	c	0.23	0.78	Uncini Manganelli and Tomei, 1999; Pieroni, 2000; Palmese et al, 2001; Camangi et al, 2003; Maccioni et al, 2004; Pieroni et al, 2004b; Guarrera et al, 2005; Pieroni and Quave, 2005; Scherrer et al, 2005; Gonzalez-Tejero et al, 2008; Guarino et al, 2008; Cornara et al, 2009; Idolo et al, 2010; Cornara et al, 2014; Menale and Muoio, 2014; Fortini et al, 2016; Menale et al, 2016	17	X	X	X	X	X	X		X	
Triticum dicoccon (Schrank) Schübl.	Poaceae	Grano	c	0.01	0.11	Pieroni, 2000	1			X						
Triticum durum Desf.	Poaceae	Grano duro	c	0.04	0.33	Pieroni et al, 2004a; Pieroni and Quave, 2005; Maxia et al, 2008	3	X		X		X				

Triticum monococcum L.	Poaceae	Grano	c	0.01	0.11	Menale et al, 2006	1		X							
Triticum sp.pl.	Poaceae	-	nd	0.03	0.78	Ballero et al, 2001; Loi et al, 2005	2	X	X	X	X	X	X		X	
Triticum turgidum L.	Poaceae	Grano	c	0.01	0.22	Lucchetti et al, 2019	1		X						X	
Tropaeolum majus L.	Tropaeolaceae	Nasturzio	c	0.01	0.11	Danna et al, 2022	1	X								
Tussilago farfara L.	Compositae	Tossillaggine	w	0.39	0.89	Lokar and Poldrini, 1988; Leporatti and Ivancheva, 2003; Pieroni et al, 2004a; Guarrera et al, 2005; Pieroni and Quave, 2005; Scherrer et al, 2005; Menale et al, 2006; Gonzalez-Tejero et al, 2008; Guarino et al, 2008; Pieroni and Giusti, 2009; Vitalini et al, 2009; Idolo et al, 2010; Leto et al, 2012; Vitalini et al, 2012; Di Novella et al, 2013; Di Sanzo et al, 2013; Cornara et al, 2014; Tuttolomondo et al, 2014; Tuttolomondo et al, 2014b; Bellia and Pieroni, 2015; Dei Cas et al, 2015; Vitalini et al, 2015; Fortini et al, 2016; Menale et al, 2016; Ranfa and Bodesmo, 2017; Maruca et al, 2019; Mattalia et al, 2020a; Petalka et al, 2020; Danna et al, 2022	29	X	X	X	X	X	X	X		X
Typha angustifolia L.	Typhaceae	Lisca minore	w	0.03	0.22	Tuttolomondo et al, 2014; Tuttolomondo et al, 2014b	2	X	X							
Typha latifolia L.	Typhaceae	Lisca maggiore	w	0.01	0.22	Motti et al, 2009	1		X					X		
Ulmus minor Mill.	Ulmaceae	Olmo	w	0.19	0.78	Leporatti et al, 1985b; Lokar and Poldrini, 1988; Camangi et al, 2003; Leporatti and Ivancheva, 2003; Pieroni et al, 2004a; Pieroni et al, 2004b; Guarrera et al, 2005; Pieroni and Quave, 2005; Menale et al, 2006; Gonzalez-Tejero et al, 2008; Guarino et al, 2008; Savo et al, 2011; Guarrera et al, 2015; Menale et al, 2016	14	X	X	X	X	X		X	X	
Ulmus sp.pl.	Ulmaceae	-	nd	0.01	0.33	Idolo et al, 2010; Mattalia et al, 2019	1	X	X					X		
Umbilicus horizontalis (Guss.) DC.	Crassulaceae	-	w	0.09	0.33	Palmese et al, 2001; Passalacqua et al, 2007; Guarino et al, 2008; Signorini et al, 2009; Leto et al, 2012; Tuttolomondo et al, 2014; Motti and Motti, 2017	7	X	X	X						
Umbilicus rupestris (Salisb.) Dandy	Crassulaceae	Ombelico di Venere	w	0.24	0.56	Bruni et al, 1997; Uncini Manganelli and Tomei, 1999; Ballero et al, 2001; Palmese et al, 2001; Loi et al, 2004; Maccioni et al, 2004; Pieroni et al, 2004a; Guarrera et al, 2005; Loi et al, 2005; Pieroni and Quave, 2005; Gonzalez-Tejero et al, 2008; Guarino et al, 2008; Cornara et al, 2009; Leto et al, 2012; Di Novella et al, 2013; Di Sanzo et al, 2013; Tuttolomondo et al, 2014b; Maruca et al, 2019	18	X	X	X	X	X				
Urospermum dalechampii (L.) Scop. ex F.W.Schmidt	Compositae	Boccione maggiore	w	0.04	0.33	Cornara et al, 2009; Tuttolomondo et al, 2014; Geraci et al, 2018	3	X		X	X					

Urtica atrovirens Req. ex Loisel.	Urticaceae	Ortica verde	w	0.01	0.11	Loi et al, 2005	1	X								
Urtica dioica L.	Urticaceae	Ortica comune	w	0.76	1.00	Leporatti et al, 1985b; Lokar and Poldrini, 1988; Bruni et al, 1997; Uncini Manganelli and Tomei, 1999; Pieroni, 2000; Leporatti and Corradi, 2001; Camangi et al, 2003; Leporatti and Ivancheva, 2003; Loi et al, 2004; Maccioni et al, 2004; Pieroni et al, 2004b; Guarrera et al, 2005a; Loi et al, 2005; Pieroni and Quave, 2005; Scherrer et al, 2005; De Natale and Pollio, 2007; Guarrera and Lucia, 2007; Passalacqua et al, 2007; Gonzalez-Tejero et al, 2008; Guarino et al, 2008; Maxia et al, 2008; Cornara et al, 2009; Pieroni and Giusti, 2009; Signorini et al, 2009; Vitalini et al, 2009; Leto et al, 2012; Mattalia et al, 2012; Montesano et al, 2012; Vitalini et al, 2012; Di Novella et al, 2013; Di Sanzo et al, 2013; Cornara et al, 2014; Menale and Muoio, 2014; Tuttolomondo et al, 2014; Tuttolomondo et al, 2014b; Bellia and Pieroni, 2015; Dei Cas et al, 2015; Vitalini et al, 2015; Fortini et al, 2016; Menale et al, 2016; Geraci et al, 2018; Bruschi et al, 2019; Bruschi et al, 2019; Lucchetti et al, 2019; Maruca et al, 2019; Mautone et al, 2019; Bottoni et al, 2020; Fontefrancesco and Pieroni, 2020; Mattalia et al, 2020a; Mattalia et al, 2020b; Motti et al, 2020; Petalka et al, 2020; Mattalia et al, 2021; Menale et al, 2021; Danna et al, 2022	56	X	X	X	X	X	X	X	X	X
Urtica membranacea Poir. ex Savigny	Urticaceae	Ortica membranosa	w	0.15	0.78	Palmese et al, 2001; Menale et al, 2006; Passalacqua et al, 2007; Leonti et al, 2009; Signorini et al, 2009; Tuttolomondo et al, 2014; Fortini et al, 2016; Menale et al, 2016; Motti and Motti, 2017; Geraci et al, 2018; Motti et al, 2020	11	X	X	X	X	X		X	X	
Urtica pilulifera L.	Urticaceae	-	w	0.03	0.33	Tuttolomondo et al, 2014a; Geraci et al, 2018	2	X	X		X					
Urtica rupestris Guss.	Urticaceae	Ortica minore	w	0.01	0.33	Tuttolomondo et al, 2014a	1	X			X				X	
Urtica sp.pl.	Urticaceae	-	nd	0.07	1.00	Ballero et al, 2001; Pieroni et al, 2004a; Leporatti and Ghedira, 2009; Idolo et al, 2010; Savo et al, 2011; Sansanelli and Tassoni, 2014	6	X	X	X	X	X	X	X	X	X
Urtica urens L.	Urticaceae	Ortica minore	w	0.14	1.00	Pieroni, 2000; Palmese et al, 2001; Menale and Muoio, 2014; Tuttolomondo et al, 2014; Vitalini et al, 2015; Gargano et al, 2018; Geraci et al, 2018; Mautone et al, 2019; Motti et al, 2020; Petalka et al, 2020	10	X	X	X	X	X	X	X	X	X
Vaccinium myrtillus L.	Ericaceae	Mirtillo nero	w	0.19	1.00	Leporatti et al, 1985b; Lokar and Poldrini, 1988; Pieroni, 2000; Leporatti and Ivancheva, 2003; Vitalini et al, 2012; Cornara et al, 2014; Dei Cas et al, 2015; Vitalini et al, 2015; Fortini et al, 2016;	14	X	X	X	X	X	X	X	X	X

						Bruschi et al, 2019; Mattalia et al, 2019; Fontefrancesco and Pieroni, 2020; Petalka et al, 2020; Danna et al, 2022										
Vaccinium vitis-idaea L.	Ericaceae	Mirtillo rosso	w	0.09	0.78	Lokar and Poldrini, 1988; Leporatti and Ivancheva, 2003; Vitalini et al, 2012; Dei Cas et al, 2015; Bruschi et al, 2019; Petalka et al, 2020; Danna et al, 2022	7	X		X	X		X	X	X	X
Valeriana celtica L.	Caprifoliaceae	Valeriana europea	w	0.01	0.22	Danna et al, 2022	1	X					X			
Valeriana montana L.	Caprifoliaceae	Valeriana montana	w	0.01	0.44	Petalka et al, 2020	1				X	X		X		X
Valeriana officinalis L.	Caprifoliaceae	Valeriana comune	w/c	0.22	0.89	Lokar and Poldrini, 1988; Leporatti and Ivancheva, 2003; Menale et al, 2006; De Natale and Pollio, 2007; Guarino et al, 2008; Di Novella et al, 2013; Cornara et al, 2014; Menale and Muoio, 2014; Tuttolomondo et al, 2014b; Tuttolomondo et al, 2014b; Vitalini et al, 2015; Fortini et al, 2016; Bruschi et al, 2019; Lucchetti et al, 2019; Petalka et al, 2020; Mattalia et al, 2021; Danna et al, 2022	16	X	X	X	X	X	X	X	X	
Valeriana sp.pl.	Caprifoliaceae	-	nd	0.01	0.11	Idolo et al, 2010	1						X			
Valeriana tripteris L.	Caprifoliaceae	-	w	0.01	0.11	Guarino et al, 2008	1						X			
Valerianella carinata Loisel.	Caprifoliaceae	-	w	0.01	0.22	Pieroni, 2000	1	X		X						
Valerianella locusta (L.) Laterr.	Caprifoliaceae	Gallinella comune	w	0.03	0.22	Maccioni et al, 2004; Guarino et al, 2008	2			X						X
Veratrum album L.	Melanthiaceae	Veratro	w	0.05	0.56	Leporatti and Ivancheva, 2003; Guarino et al, 2008; Vitalini et al, 2012; Petalka et al, 2020	4		X	X			X		X	X
Veratrum lobelianum Bernh.	Melanthiaceae	Veratro	w	0.01	0.11	Cornara et al, 2014	1							X		
Veratrum nigrum L.	Melanthiaceae	Veratro scuro	w	0.01	0.33	Guarino et al, 2008	1						X		X	X
Verbascum chaixii Vill.	Scrophulariaceae	-	w	0.01	0.44	Lokar and Poldrini, 1988	1	X	X	X		X				
Verbascum densiflorum Bertol.	Scrophulariaceae	Verbasco	w	0.03	0.33	Leporatti and Ivancheva, 2003; Petalka et al, 2020	2		X			X	X			
Verbascum macrurum Ten.	Scrophulariaceae	-	w	0.01	0.11	Guarino et al, 2008	1					X				
Verbascum nigrum L.	Scrophulariaceae	Verbasco nero	w	0.01	0.11	Pieroni and Giusti, 2009	1					X				
Verbascum phlomoides L.	Scrophulariaceae	Verbasco porporino	w	0.05	0.44	Leporatti et al, 1985b; Leporatti and Ivancheva, 2003; Passalacqua et al, 2007; Petalka et al, 2020	4		X	X		X	X			
Verbascum pulverulentum Vill.	Scrophulariaceae	Verbasco candelabro	w	0.01	0.11	Bruni et al, 1997	1		X							
Verbascum sinuatum L.	Scrophulariaceae	Verbasco sinuoso	w	0.12	0.44	Uncini Manganelli and Tomei, 1999; Leporatti and Ivancheva, 2003; Guarino et al, 2008; Leporatti and Ghedira, 2009; Leto et al, 2012; Tuttolomondo et al, 2014; Tuttolomondo et al, 2014a; Motti and Motti, 2017; Gargano et al, 2018	9	X	X	X		X				

Verbascum sp.pl.	Scrophulariaceae	-	nd	0.01	0.22	Idolo et al, 2010	1		X						X	
Verbascum thapsus L.	Scrophulariaceae	Tasso barbasso	w	0.28	0.78	Uncini Manganelli and Tomei, 1999; Pieroni, 2000; Camangi et al, 2003; Leporatti and Ivancheva, 2003; Maccioni et al, 2004; Pieroni and Quave, 2005; Passalacqua et al, 2007; Gonzalez-Tejero et al, 2008; Guarino et al, 2008; Mattalia et al, 2012; Di Novella et al, 2013; Di Sanzo et al, 2013; Cornara et al, 2014; Tuttolomondo et al, 2014b; Bellia and Pieroni, 2015; Dei Cas et al, 2015; Vitalini et al, 2015; Fortini et al, 2016; Menale et al, 2016; Petalka et al, 2020; Danna et al, 2022	21	X	X	X	X	X	X		X	
Verbena officinalis L.	Verbenaceae	Verbena comune	w	0.27	1.00	Uncini Manganelli and Tomei, 1999; Leporatti and Corradi, 2001; Camangi et al, 2003; Leporatti and Ivancheva, 2003; Guarrera et al, 2005; Loi et al, 2005; Menale et al, 2006; De Natale and Pollio, 2007; Gonzalez-Tejero et al, 2008; Guarino et al, 2008; Leporatti and Ghedira, 2009; Vitalini et al, 2009; Leto et al, 2012; Di Sanzo et al, 2013; Tuttolomondo et al, 2014; Bellia and Pieroni, 2015; Geraci et al, 2018; Lucchetti et al, 2019; Petalka et al, 2020; Danna et al, 2022	20	X	X	X	X	X	X	X	X	X
Veronica allionii Vill.	Plantaginaceae	-	w	0.01	0.11	Bellia and Pieroni, 2015	1				X					
Veronica alpina L.	Plantaginaceae	Veronica alpina	w	0.01	0.44	Petalka et al, 2020	1					X	X	X		X
Veronica beccabunga L.	Plantaginaceae	-	w	0.07	0.44	Pieroni et al, 2004a; Pieroni and Quave, 2005; Pieroni et al, 2005; Guarino et al, 2008; Vitalini et al, 2015	5				X	X		X		X
Veronica chamaedrys L.	Plantaginaceae	Veronica comune	w	0.04	0.78	Lokar and Poldrini, 1988; Cornara et al, 2014; Petalka et al, 2020	3	X	X	X	X	X	X	X		
Veronica cymbalaria Bodard	Plantaginaceae	Veronica	w	0.01	0.11	De Natale and Pollio, 2007	1	X								
Veronica fruticans Jacq.	Plantaginaceae	-	w	0.01	0.11	Danna et al, 2022	1				X					
Veronica hederifolia L.	Plantaginaceae	Veronica foglie di edera	w	0.01	0.22	Leporatti and Corradi, 2001	1	X	X							
Veronica officinalis L.	Plantaginaceae	Veronica medicinale	w	0.04	0.67	Leporatti and Ivancheva, 2003; Guarino et al, 2008; Petalka et al, 2020	3	X		X	X	X	X	X		
Veronica persica Poir.	Plantaginaceae	Veronica comune	w	0.01	0.22	Leporatti and Corradi, 2001	1	X	X							
Viburnum opulus L.	Adoxaceae	Palla di neve	w/c	0.01	0.11	Leporatti and Ivancheva, 2003	1				X					
Viburnum tinus L.	Adoxaceae	Laurotino	w/c	0.03	0.33	Loi et al, 2004; Guarino et al, 2008	2	X		X		X				
Vicia faba L.	Fabaceae	Fava	w	0.16	0.89	Palmese et al, 2001; Camangi et al, 2003; Pieroni et al, 2004b; Guarrera et al, 2005; Guarrera and Lucia, 2007; Passalacqua et al, 2007; Guarino et al, 2008; Maxia et al, 2008; Savo et al, 2011; Menale et al, 2016; Lucchetti et al, 2019; Menale et al, 2021	12	X	X	X	X		X	X	X	X
Vicia hirsuta (L.) Gray	Fabaceae	Vecia pelosa	w	0.01	0.11	Tuttolomondo et al, 2014	1		X							

Vicia sativa L.	Fabaceae	Vecia	w	0.03	0.22	Guarino et al, 2008; Lucchetti et al, 2019	2	X	X							
Vinca difformis subsp. *sardoa* Stearn	Apocynaceae	-	w	0.01	0.11	Loi et al, 2005	1	X								
Vinca major L.	Apocynaceae	Pervinca	w/c	0.03	0.44	De Natale and Pollio, 2007; Leto et al, 2012	2	X		X	X	X				
Vinca minor L.	Apocynaceae	Pervinca minore	w/c	0.03	0.67	Leporatti and Ivancheva, 2003; Vitalini et al, 2015	2	X	X	X	X			X		X
Vinca sp.pl.	Apocynaceae	-	nd	0.01	0.78	Lokar and Poldrini, 1988	1	X	X	X	X		X	X		X
Vincetoxicum hirundinaria Medik.	Apocynaceae	Vincetossico	w	0.03	0.56	Lokar and Poldrini, 1988; Mautone et al, 2019	2	X		X		X		X	X	
Viola alba Besser	Violaceae	Viola bianca	w	0.04	0.11	Tuttolomondo et al, 2014a; Tuttolomondo et al, 2014b; Lucchetti et al, 2019	3					X				
Viola alba subsp. *dehnhardtii* (Ten.) W.Becker	Violaceae	Viola bianca	w	0.01	0.33	Guarino et al, 2008	1		X			X			X	
Viola arvensis Murray	Violaceae	-	w	0.03	0.33	Guarino et al, 2008; Menale et al, 2016	2		X			X			X	
Viola biflora L.	Violaceae	Viola gialla	w	0.03	0.33	Vitalini et al, 2012; Petalka et al, 2020	2	X		X			X			
Viola calcarata L.	Violaceae	-	w	0.04	0.33	Bellia and Pieroni, 2015; Fontefrancesco and Pieroni, 2020; Danna et al, 2022	3	X		X		X				
Viola eugeniae Parl.	Violaceae	-	w	0.01	0.11	Fortini et al, 2016	1			X						
Viola odorata L.	Violaceae	Viola comune	w	0.16	0.78	Leporatti and Ivancheva, 2003; Pieroni et al, 2004b; De Natale and Pollio, 2007; Guarino et al, 2008; Pieroni and Giusti, 2009; Idolo et al, 2010; Mattalia et al, 2012; Di Novella et al, 2013; Fortini et al, 2016; Mattalia et al, 2019; Mautone et al, 2019; Petalka et al, 2020	12	X	X	X	X	X	X	X		
Viola pseudogracilis Strobl	Violaceae	-	w	0.01	0.33	Guarino et al, 2008	1		X			X			X	
Viola reichenbachiana Jord. ex Boreau	Violaceae	-	w	0.04	0.56	Leporatti et al, 1985b; Guarino et al, 2008; Fortini et al, 2016	3	X	X		X	X			X	
Viola sp.pl.	Violaceae	-	nd	0.05	0.67	Lokar and Poldrini, 1988; Guarrera et al, 2005; Passalacqua et al, 2007; Bruschi et al, 2019	4	X	X	X	X	X		X		
Viola tricolor L.	Violaceae	Viola a tre colori	w	0.07	0.78	Leporatti and Ivancheva, 2003; Bellia and Pieroni, 2015; Vitalini et al, 2015; Petalka et al, 2020; Danna et al, 2022	5	X	X	X	X	X			X	X
Viscum album L.	Santalaceae	Vischio	w	0.11	0.67	Leporatti and Corradi, 2001; Leporatti and Ivancheva, 2003; Menale et al, 2006; Passalacqua et al, 2007; Guarino et al, 2008; Leto et al, 2012; Tuttolomondo et al, 2014b; Petalka et al, 2020	8	X	X		X		X	X	X	
Vitex agnus-castus L.	Lamiaceae	Agnocasto	c	0.03	0.33	Guarino et al, 2008; Geraci et al, 2018	2	X			X		X			
Vitis vinifera L.	Vitaceae	Vite	w/c	0.38	1.00	Uncini Manganelli and Tomei, 1999; Pieroni, 2000; Ballero et al, 2001; Leporatti and Corradi, 2001; Palmese et al, 2001; Camangi et al, 2003; Maccioni et al, 2004; Pieroni et al, 2004a; Pieroni et al, 2004b; Guarrera et al, 2005; Guarrera et al, 2005a; Loi et al, 2005; Pieroni and Quave, 2005; Passalacqua et al, 2007; Gonzalez-Tejero et al, 2008; Guarino et al,	28	X	X	X	X	X	X	X	X	X

						2008; Maxia et al, 2008; Leporatti and Ghedira, 2009; Pieroni and Giusti, 2009; Savo et al, 2011; Cornara et al, 2014; Fortini et al, 2016; Menale et al, 2016; Motti and Motti, 2017; Lucchetti et al, 2019; Mautone et al, 2019; Menale et al, 2021; Danna et al, 2022										
Xanthium spinosum L.	Compositae	Nappola spinosa	w	0.04	0.33	Palmese et al, 2001; De Natale and Pollio, 2007; Guarino et al, 2008	3	X		X			X			
Xanthium strumarium L.	Compositae	Nappola	w	0.01	0.33	Leporatti and Ghedira, 2009	1			X	X	X				
Zea mays L.	Poaceae	Mais	c	0.34	0.78	Leporatti et al, 1985b; Bruni et al, 1997; Pieroni, 2000; Leporatti and Corradi, 2001; Camangi et al, 2003; Leporatti and Ivancheva, 2003; Pieroni et al, 2004a; Pieroni et al, 2004b; Maccioni et al, 2004; Pieroni and Quave, 2005; Menale et al, 2006; Gonzalez-Tejero et al, 2008; Guarino et al, 2008; Leporatti and Ghedira, 2009; Motti et al, 2009; Di Sanzo et al, 2013; Cornara et al, 2014; Menale and Muoio, 2014; Tuttolomondo et al, 2014b; Dei Cas et al, 2015; Vitalini et al, 2015; Menale et al, 2016; Motti and Motti, 2017; Mautone et al, 2019; Menale et al, 2021	25	X	X	X	X	X		X		X
Zingiber officinale Roscoe	Zingiberaceae	Zenzero	c	0.01	0.33	Bruschi et al, 2019	1	X		X		X				
Ziziphus jujuba Mill.	Rhamnaceae	Giuggiolo	c	0.08	0.33	Pieroni et al, 2004a; Loi et al, 2005; Pieroni and Quave, 2005; Mattalia et al, 2020a; Mattalia et al, 2020b; Menale et al, 2021	6					X	X		X	
